# Supplementary material for: HypoRiPPAtlas as an Atlas of hypothetical natural products for mass spectrometry database search
Source: Nat Commun. 2023 Jul 14;14:4219. doi: 10.1038/s41467-023-39905-4 (PMC10349150; doi:10.1038/s41467-023-39905-4)
Supplement: Supplementary file 1 — Supplementary Information [file 41467_2023_39905_MOESM1_ESM.pdf]

Supplementary Information for

HypoRiPPAtlas as an Atlas of hypothetical natural products for mass spectrometry database search

Yi-Yuan Lee[1,2,+], **Mustafa Guler**[1,+], **Desnor N. Chigumba**[3], **Shen Wang**[1], **Neel Mittal**[1], **Cameron Miller**[1], **Benjamin Krummenacher**[1], **Haodong Liu**[1], **Liu Cao**[1], **Aditya Kannan**[1], **Keshav Narayan**[1], **Samuel T Slocum**[4], **Bryan L Roth**[4], **Alexey Gurevich**[5,6], **Bahar Behsaz**[1], **Roland D. Kersten**[3], and **Hosein Mohimani**[1,*]

[1]Carnegie Mellon University, Pittsburgh, 15213, United States of America
[2]Cornell University, Ithaca, 14850, United States of America
[3]Department of Medicinal Chemistry, University of Michigan, Ann Arbor, USA
[4]Department of Pharmacology, University of North Carolina, Chapel Hill, USA
[5]Helmholtz Institute for Pharmaceutical Research Saarland (HIPS), Helmholtz Centre for Infection Research, Saarbrücken, Germany
[6]Department of Computer Science, Saarland University, Saarbrücken, Germany
[+]These authors contributed equally
[*]Corresponding author: hoseinm@andrew.cmu.edu

Supplementary Notes

S1. Materials. All chemicals were purchased from Fisher Scientific, unless otherwise noted. Synthetic gene *EpuBURP-truncated* was purchased from GenScript, Inc. Solvents for liquid chromatography high-resolution mass spectrometry were Optima® LC-MS grade (Fisher Scientific). High-resolution mass spectrometry analysis was performed on a Thermo ESI-Q-Exactive Orbitrap mass spectrometer coupled to a Thermo Vanquish UHPLC system. NMR analysis was performed on a Bruker Ascend 800 MHz NMR spectrometer equipped with a 5mm Triple resonance inverse detection TCI cryoprobe. Preparative and semipreparative HPLC was performed on a Shimadzu LC-20AP liquid chromatograph equipped with a SPD-20A UV/VIS detector and a FRC-10A fraction collector. *Elaeagnus pungens* was purchased from Wilson Bros Gardens (GA, USA) as adult plants. *Streptomyces rimosus* subsp. *rimosus* NRRL B-2660 was obtained from the NRRL Agricultural Research Service Culture Collection. Botanical garden plant samples were collected from the Matthaei Botanical Garden at the University of Michigan, Ann Arbor, in August 2019 and August 2020 [1]. *Glycine max* seeds for cultivation were purchased from Outsidepride™. *Arachis hypogaea* seeds for cultivation were purchased from KAFA company. *Lycium barbarum* root was purchased from 9GreenBox™. *Cercis canadensis* leaf and stem tissue was collected at 428 Church Street, Ann Arbor, MI 48109. *Solanum melongena* seeds for cultivation were purchased from Seedz.

S2. Plant transcriptomics. Plant transcriptomic raw-datasets (Supplementary Table 2) were downloaded from the NCBI Sequence Read Archive and assembled de novo by SPAdes (v3.13) [2].

S3. Metabolomics. *N. benthamiana, A. hypogaea, G. max, S. melongena, M. truncatula* were grown in SunGro Sunshine® Mix 4 soil with added vermiculite (Plantain Inc.) and added Osmocote fertilizer under plant growth lights with a 16 h light/8 h dark cycle for three months. *Streptomyces rimosus* subsp. *rimosus* NRRL B-2660 was grown from glycerol stock on ISP2-agar (Yeast extract 4 g, Malt extract 10 g, Glucose 4 g, Agar 20 g, 1 L deionized water, pH adjusted to 7.3) for 7 days at 28 ℃. For liquid cultivation, a 5 mL ISP2 starter culture was inoculated from S. *rimosus* NRRL B-2660 spores from the ISP2-agar plate and incubated at 28 ℃ and 220 rpm for 24 h. Then a 50 mL ISP2 liquid culture was inoculated with 1 mL of the starter culture and incubated for 7 days at 28 ℃ and 220 rpm. Subsequently, the culture was centrifuged at 3400 g for 15 min and the supernatant was extracted twice with 50 mL n-butanol. The n-butanol extracts were combined, dried in vacuo and resuspended in 2 mL methanol for liquid chromatography mass spectrometry (LC-MS) analysis. For solid growth medium cultivation, a 15 mL ISP2-agar plate was inoculated on its surface with a spore suspension in sterile water from the initial S. *rimosus* agar plate and incubated for 7 days at 28 ℃. The S. *rimosus* colonies were then extracted with 100 mL n-butanol shaking for 1 h at 37 ℃. The n-butanol extracts were centrifuged at 4000 g for 15 min, the supernatant was dried in vacuo and resuspended in 2 mL methanol for liquid chromatography mass spectrometry (LC-MS) analysis.

Plant metabolomic samples were prepared as described before [1]. Briefly, 0.2 g fresh plant material was collected from aerial tissues, frozen and ground with a MP Biomedicals FastPrep24-5G Tissuelyser with a Coolprep cryogenic adapter in 2 mL MP Biomedicals tubes with 2.3 mm Zirconia beads (Fisher Scientific). Ground plant material was extracted with 3 mL methanol for 1 h at 37 ℃ in a 7 mL glass vial. Crude methanol extracts were dried under nitrogen gas in a separate glass vial. Dried plant methanol extracts were resuspended in water (3 mL) and partitioned twice with hexane (3 mL), twice with ethyl acetate (3 mL), and once with n-butanol (3 mL). The n-butanol fraction was dried by vacuum centrifugation in a Thermo Scientific SPD140P1 speedvac and resuspended in 2 mL methanol for LC-MS analysis. For extraction of transgenic *N. benthamiana* leaves, 0.2 g infiltrated leaves were collected, ground as described above and extracted with 1 mL 80% methanol at 60 ℃ in the 2 mL tubes in a water bath. Then, the extracts were centrifuged for 5 min at 16000 g.

All microbial and plant extracts were then filtered by Whatman syringeless filters (0.2 μm) and were subjected to LC-MS/MS analysis with the following parameters: Injection volume 5 μL, LC – Phenomenex Kinetex®2.6 μm C18 reverse phase 100 Å 150 x 3 mm LC column, LC gradient: solvent A – 0.1% formic

acid, solvent B – acetonitrile (0.1% formic acid), 0 min: 10% B, 5 min: 60% B, 5.1 min: 95% B, 6 min: 95% B, 6.1 min: 10% B, 9.9 min: 10% B, 0.5 mL/min, MS – positive ion mode, Full MS: Resolution 70000, mass range 400-1200 m/z, dd-MS^2 (data-dependent MS/MS): resolution 17500, AGC target 1e5, loop count 5, isolation width 1.0 m/z, collision energy 25 eV, dynamic exclusion 0.5 s. For MS/MS analysis of Lasso-1795 peptide, the LC-MS/MS analysis had the following MS parameters: TargetSIM-DDA: MS^1 - isolation width 4 m/z with target mass 898.4712 Da (z = 2), dd-MS^2: HCD 25 eV, MS^2 - isolation width 0.4 m/z, AGC target: 1e5. LC-MS data was analyzed with QualBrowser in the Thermo Xcalibur software package (version 4.3.73.11, ThermoScientific).

S4. Transient gene expression in *Nicotiana benthamiana*. Synthetic gene *EpuBURP-truncated-5xLPIIY* (*N. benthamiana* codon-optimized with Gibson clonin 5'-end adapter: tgcccaaattcgcgaccggt, 3'-end adapter: ctcgaggcctttaactctgg) was cloned into pEAQ-HT after restriction digest with AgeI and XhoI via Gibson cloning [3], verified by Sanger sequencing and transiently expressed as pEAQ-HT-constructs in *N. benthamiana* leaves (4-6 week-old plants) via *Agrobacterium tumefaciens* LBA4404 syringe infiltration [4] as previously described [5].

```
>EpuBURP
ATGGAACTTCGTCTTTTGTTCCTGTTCTTCTTAGTTATTCTTCACCTTGCTGGTGTTTCCTCCCAATTTGATGATAGTC
ATAATCTGCATAATGAAGGGGAGGAGGCTTTTCCTAACGTCGATAAGCTCCCAACCCAGCCCGCCGGAGGTGTCCCGGA
GAAGTTCGGGAAAGACTTGTTAAATCACATGGCCCAAAGTGGTCTTTTCAGTGGTGGGGTTAGTGAAAAATTTGGGAAA
AACCTGTTGAACCACATGGCTCAACCCGACATCTTACCAATAATCTACGGCGGTGGGGCTCATGAAAATTTTGGGAAGG
ACCTGTTGAATCACATGGCTCAGCCCGACCTGCTGCCAATCATTTATGGAGGAGGGGCCCCCGAGAAAATAGAGAAAGA
CCTCCTGAATCATAAAGCCCAAAGTGATCTTTTCTCCGGTGGCGCATCTGAGAAATTCGGCAAGGATCTCCTGAATCAT
ATGGCACAGCCTGATATGCTCCCGATTATATATGGTGGGGGTGCCCACGAGAATTTTGGCAAAGATCTTCTCAACCATA
TGGCCCAACCCGATTTATTGCCAATAATTTACGGTGGGGGTGCCCCAATGAAAGACAGTAAATTAATGAAGGGTAACGT
GGCATCCTTCTTTCTTGAAAATGACCTCCTCTTAGGGAAAACGATGAAGCTGCATTTCACAAATCCCATCACGGGAGCC
AAGTTTTTACCTCTCGACATCGCCAAATCCATTCCCTTTGCCTCCAATAAGTTACCTGAGATACTCAATAGATTCAATA
TCGAACCGAAATCTAGCGACGTTGAGATAGTCAAACAAACGATCAGCTTGTGCGAAGGTCAGAAATCCATCGAAACGGA
GCACAAATACTGCGCTACTAGTCTTGAATCTCTGATCGATTTCTCACGAAGCAAACTCGGAGAAGATATTAAGATATAC
AGCACTGAGGTGGATGAAGAAATTAAGCAAGATTACAAGATCATCAAAGAAAGCATCGTTAAGCTTGGCGACAAAAGCG
TCGTGTGTCATAAATTAAATTATATGTATGCTGTATACTATTGTCATCACGTACACGCTACTAAGGTATATATGGCTAC
GCTGGAGGGCGAAAACGGCGTCAAAGAGAACGCTATCGCCGCCTGCCATGCTGACACTAAGGGTTGGAATCCAAAACAC
CTCGCCTTTCAACTTCTCAACATCAAGCCAGGCACGGATACAATTTGCCACTTTCTCAGTTCTGATACTCTTGTACTCG
TGCCTCAGAAGGACAAGGACGTAAGCTACTGActcgaggcctttaactctgg
```

S5. Peptide isolation and structure elucidation. *Elaeagnus pungens* stem and roots (4 kg fresh weight) were cut into small pieces, ground in a food processor and extracted in 8 L methanol for 16 h at 37 ℃ and 140 rpm. Crude methanol extracts were filtered with a silica filter and dried in vacuo. Dried methanol extract was resuspended in 1 L deionized water, partitioned twice with 1 L hexane, partitioned twice with 1 L ethyl acetate, and extracted twice with 1 L n-butanol. n-butanol fractions were combined, dried in vacuo, and resuspended in 40 mL 10% methanol. Resuspended *E. pungens* extract was separated by flash-column liquid chromatography on a Sephadex LH20 solid phase with 10%, 20% and 40% methanol (500 mL each with increasing methanol concentration) as a mobile phase. LC fractions were analyzed by LC-MS analysis for target mass of elaeagnin ($[M+H]^+$ 616.3704) with the following LC-MS parameters: Injection volume 2.5 μL, LC – Phenomenex Kinetex® 2.6 μm C18 reverse phase 100 Å 50 x 3 mm LC column, LC gradient: solvent A – 0.1% formic acid, solvent B – acetonitrile (0.1% formic acid), 0 min: 5% B, 2.5 min: min: 95% B, 3.0 min: 95% B, 3.1 min: 5% B, 5.0 min: 5% B, 0.5 mL/min, MS – positive ion mode, Full MS: resolution 35000, mass range 400-1250 m/z, dd-MS^2: resolution 17500, loop count 5, collision energy 25 eV, dynamic exclusion 0.5 s. LC fractions with target peptide were combined, dried in vacuo and resuspended in 10% acetonitrile (0.1% trifluoroacetic acid (TFA)). Resuspended peptide fractions were separated twice by preparative HPLC with the following settings: Phenomenex Kinetex® 5 μm C18 100 Å LC Column 150

x 21.2 mm, LC gradients: solvent A – 0.1% TFA, solvent B – acetonitrile (0.1% TFA), 1. separation: 0 min: 10% B, 1 min: 10% B, 36 min: 50% B, 39 min: 95% B, 42 min: 95% B, 42.5 min: 10% B, 60.1 min: 10% B. 2. separation: 0 min: 20% B, 1 min: 20% B, 36 min: 40% B, 39 min: 95% B, 42 min: 95% B, 42.5 min: 20% B, 60.1 min: 20% B. Preparative HPLC fractions were analyzed for elaeagnin as described above after 1:30-dilution in LC-MS-grade water. Preparative HPLC fractions containing elaeagnin were combined, dried in vacuo and resuspended in 25% acetonitrile (0.1% TFA). Resuspended elaeagnin was subjected to two rounds of semipreparative HPLC separation with the following settings: Kinetex® 5 μm C18 100 Å LC Column 250 x 10.0 mm, LC gradients: solvent A – 0.1% TFA, solvent B – acetonitrile (0.1% TFA), 0 min: 28% B, 1 min: 28% B, 21 min: 35% B, 22 min: 95% B, 24.5 min: 95%, 25 min: 28% B, 45 min: 28% B. Semipreparative HPLC fractions were analyzed for elaeagnin as described above. Final elaeagnin fractions were combined and dried in vacuo to yield elaeagnin as a white powder (3 mg).

S6. Structure elucidation of elaeagnin. Elaeagnin was analyzed by 1D and 2D NMR in MeOD-d4 (Supplementary Figure 16, Supplementary Table 2) in a corresponding Shigemi NMR tube (Wilson Glass). NMR data analysis was done with Bruker Topspin (version 4.0.8). The spin systems of all amino acids of the predicted core peptide LPIIY were determined by COSY/TOCSY/HMBC. The core peptide sequence LPIIY was partially confirmed by HMBC (Leu1-Pro2 and Ile4-Tyr5) and further inferred based on the detection of an analyte with the calculated mass of a monocyclic LPIIY peptide after transient expression of truncated precursor peptide EpuBURP which only included the core peptide LPIIY (Supplementary Figure 18). The macrocyclization was defined as an ether bond between Pro2-Cβ to Tyr5-C4 by (a) a proline with a single Hβ, (b) a $\delta(^{13}C)$ at Pro-Cβ of 79.2 ppm which is similar to other $\delta(^{13}C)$ of macrocyclic sites via C-O-bonds [1], (c) NOE correlations of Pro-Hβ (δ 5.36 ppm, strong NOE signal) and Pro-Hα (δ 4.25 ppm, weak NOE signal) to Tyr-H3/5 (δ 6.69 ppm). No HMBC signal was detected for the correlation of Pro-Hβ with Tyr5-C4 which is in agreement with a similar plant peptide with a Pro-Cβ-O-Tyr-C4-crosslink called rhopeptin A, which is not biosynthetically characterized [6]. The stereochemistry of elaeagnin amino acids was determined by Marfey's analysis and chiral HPLC of hydrolyzed amino acids as follows: 0.3 mg of elaeagnin were stirred in 1 mL 6 N HCl at 110 ℃ for 16 h. The hydrolysate was dried in vacuo and resuspended in 2 mL water three times, applied to a Strata C8 SPE column (Phenomenex, 100 mg), washed with 1.2 mL water and eluted with 2 mL 50% acetonitrile. The eluate was dried in vacuo, resuspended in 100 μL water and added with 200 μL Marfey's reagent (Thermo Scientific, 1% (w/v) acetone) and with 40 μL sodium bicarbonate (1 M) before incubation for 1 h at 40 ℃. The reaction was quenched with 20 μL 2 N HCl, diluted 1:1 with 50% acetonitrile and subjected to LC-MS analysis as for plant and microbial extract metabolomic analysis with the mass range: 100-800 m/z. Retention times for Marfey's analysis were as follows: Leu: 5.93 min (std: L-Leu, 5.92 min, D-Leu, 6.34 min), Pro: 4.84 min (std: L-Pro, 4.84 min, D-Pro, 4.95 min), Ile: 5.84 min (std: L-Ile, 5.83 min, D-Ile, 6.28 min), Tyr: 5.04 min (std: L-Tyr, 5.03 min, D-Tyr, 5.18 min). The stereochemistry of the macrocyclic bond was determined as Pro-Cβ-(S) by comparison of the Pro-$J_{\alpha\beta}$ coupling constant of 4.6 Hz in comparison to similar L-Pro-Cβ-O-Tyr-crosslinks [7].

Supplementary Figures

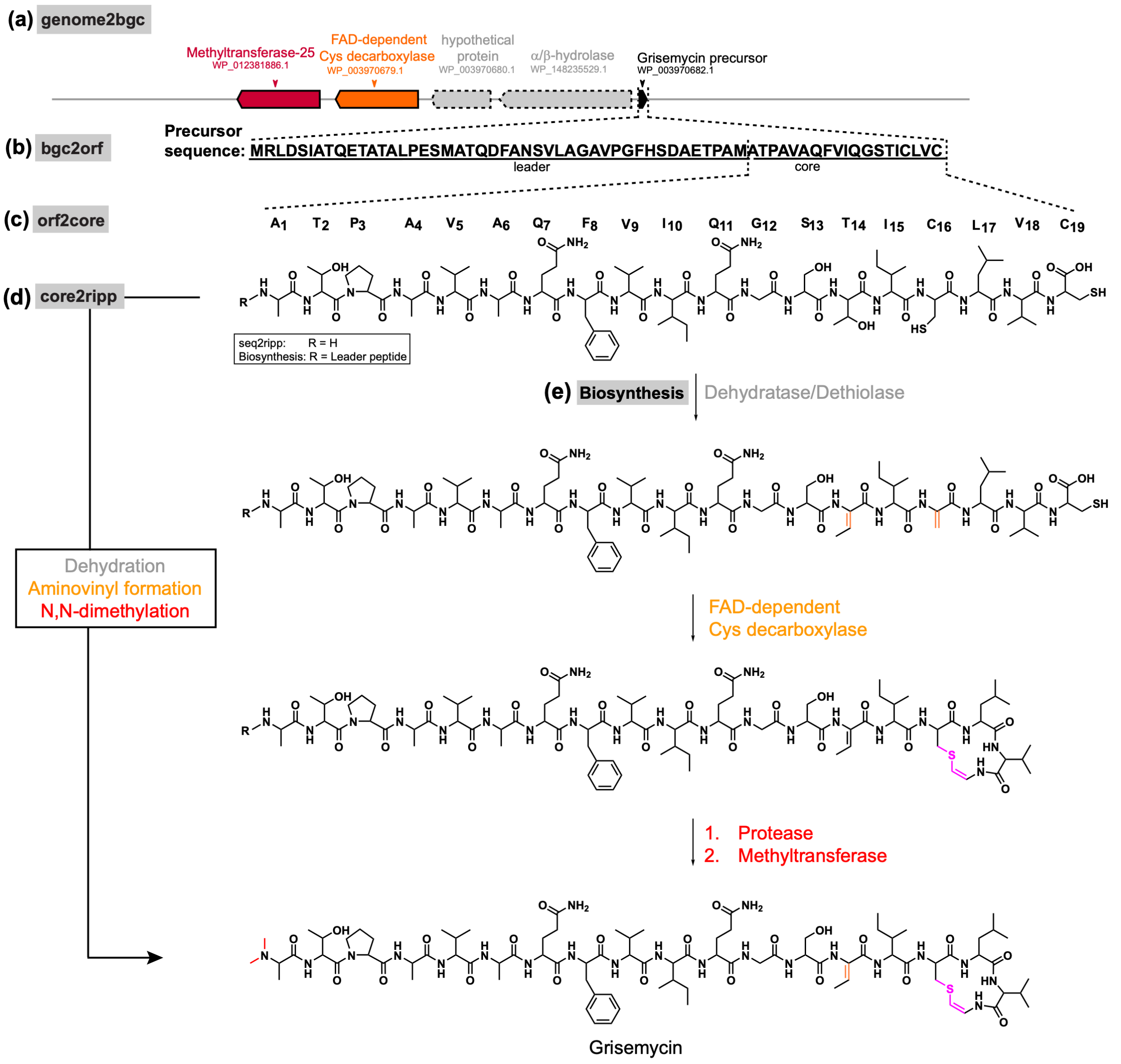

Supplementary Figure 1: Identification of grisemycin by seq2ripp. (a) Grisemycin BGC is identified through its modification enzymes based on BGC-derived Pfams. (b) Short ORFs are detected within the BGC as candidate structural ORFs including the grisemycin precursor gene. (c) Fragments of the structural ORFs are extracted as candidate precursor peptides including the grisemycin precursor peptide. (d) Depending on the tailoring enzymes in the BGC, corresponding modifications are (optionally) applied (highlighted in orange/ grey/red) to the core peptides to form hypothetical molecules by seq2ripp in a single step including the grisemycin structure. (e) seq2ripp prediction is informed by biosynthetic knowledge of linaridins.

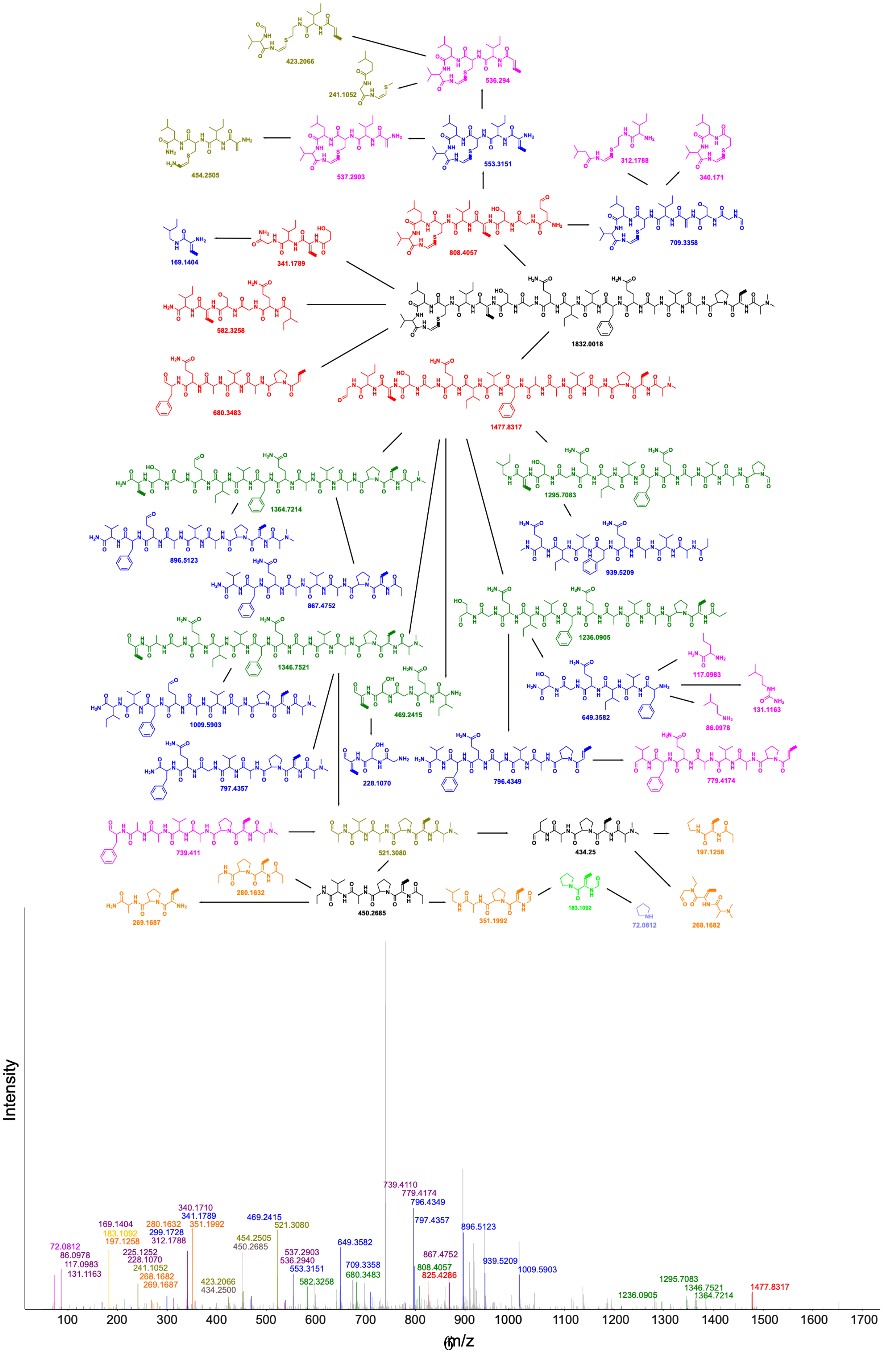

Supplementary Figure 2: Annotation of grisemycin tandem mass spectrum based on Dereplicator+ model.

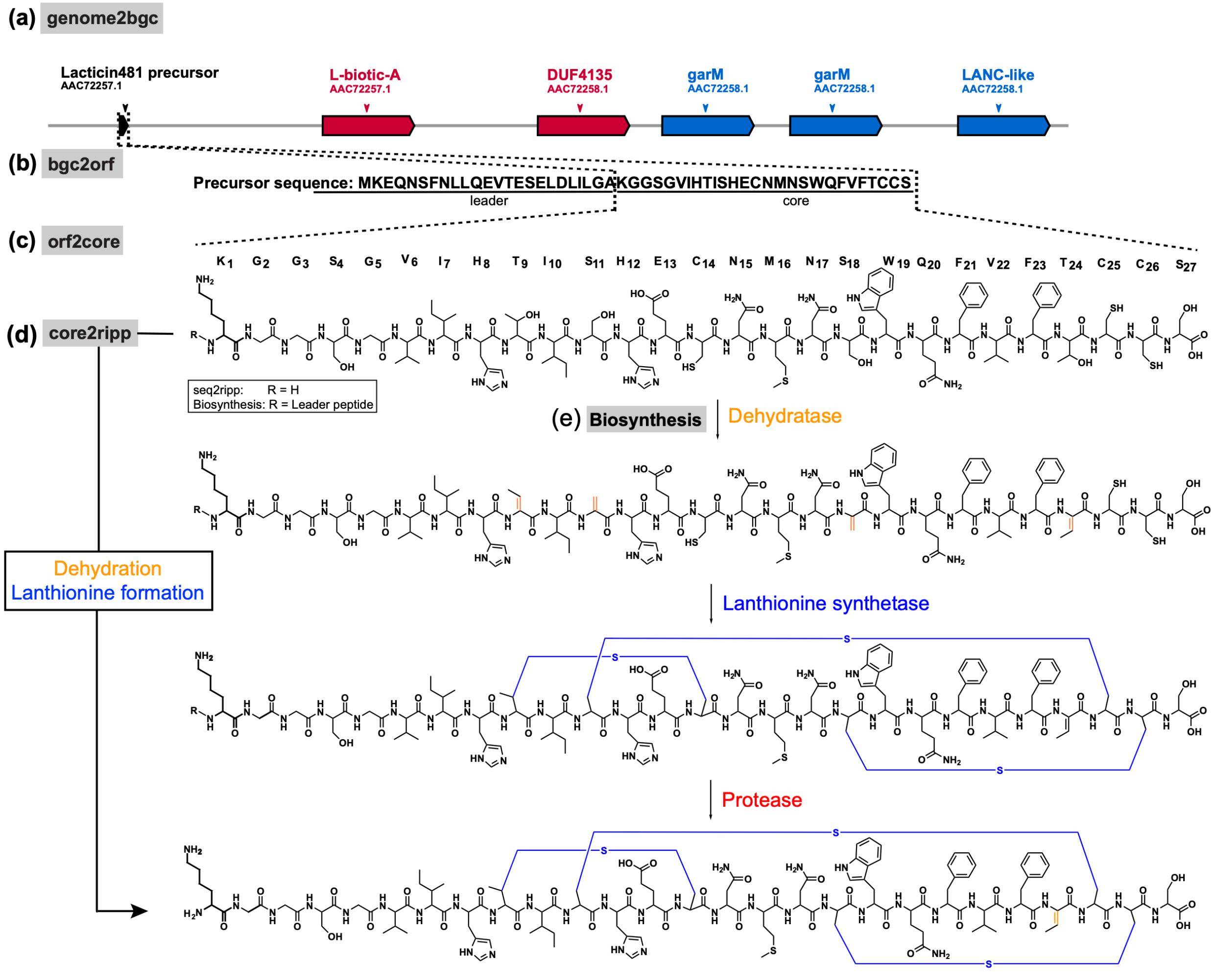

Supplementary Figure 3: Identification of lacticin 481 by seq2ripp. (a) Lacticin 481 BGC is identified through its modification enzymes based on BGC-derived Pfams. (b) Short ORFs are detected within the BGC as candidate structural ORFs including the lacticin 481 precursor gene. (c) Fragments of the structural ORFs are extracted as candidate precursor peptides including the lacticin 481 precursor peptide. (d) Depending on the tailoring enzymes in the BGC, corresponding modifications are (optionally) applied (highlighted in orange/ blue) to the core peptides to form hypothetical molecules by seq2ripp in a single step including the lacticin 481 structure. (e) seq2ripp prediction is informed by biosynthetic knowledge of lanthipeptides.

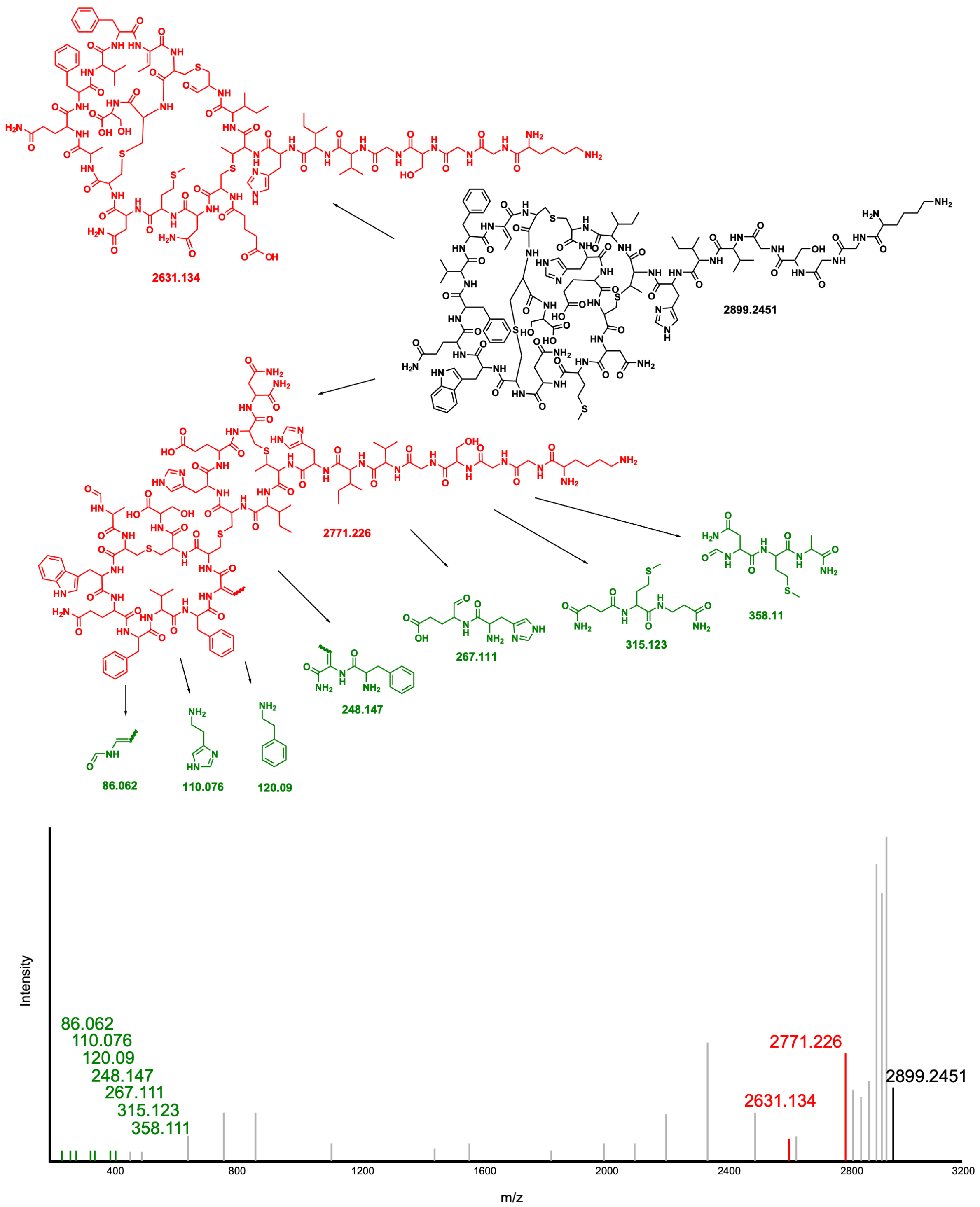

Supplementary Figure 4: Annotation of lacticin 481 tandem mass spectrum based on Dereplicator+ model.

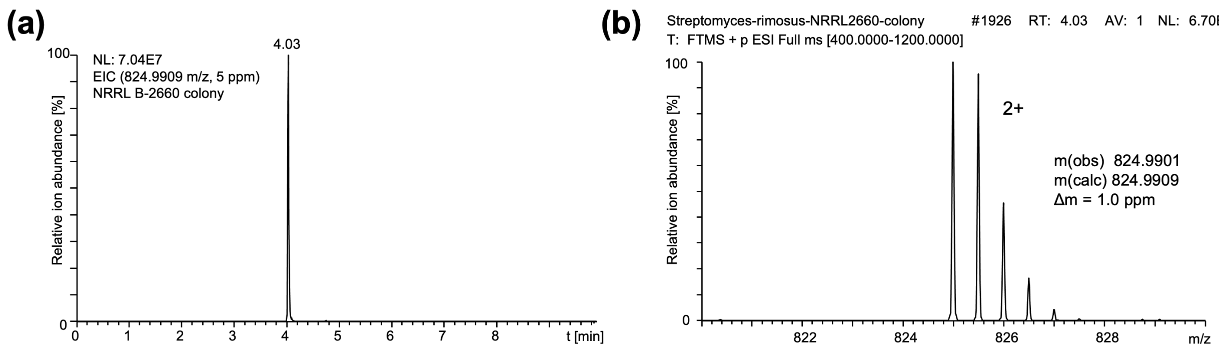

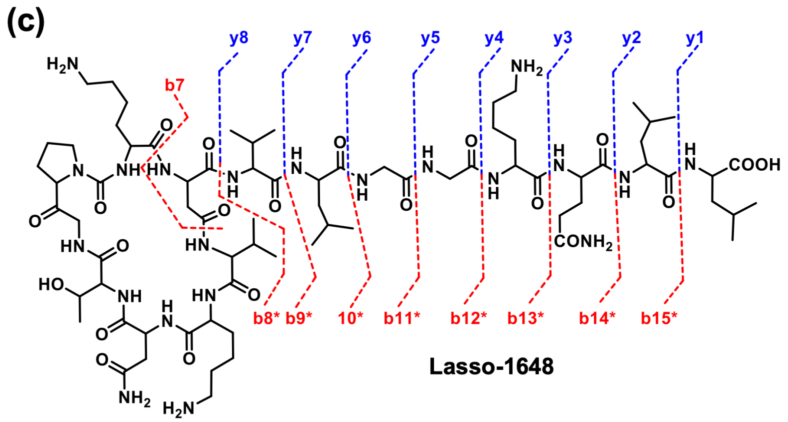

Fragment (z)	m(obs)	m(calc)	Δm [ppm]
y1 (1+)	132.1020	132.1021	1.1
y2 (1+)	245.1860	245.1861	0.4
y3 (1+)	373.2446	373.2441	1.3
y4 (1+)	501.3396	501.3414	3.7
y5 (1+)	558.3610	558.3615	0.9
y6 (1+)	615.3825	615.3830	0.9
b7 (1+)	725.4315	725.4305	1.4
y7 (1+)	728.4665	728.4688	3.1
b8* (1+)	822.4484	822.4468	2
y8 (1+)	827.5350	827.5350	0
b9* (1+)	921.5157	921.5153	0.4
b10* (1+)	1034.5996	1034.5993	0.3
b11* (1+)	1091.6227	1091.6208	1.7
b12* (1+)	1148.6404	1148.6422	1.6
b13* (1+)	1276.7358	1276.7372	1.1
b14* (1+)	1404.7950	1404.7958	0.6
b15* (2+)	1517.8829	1517.8798	2
b16* (2+)	1630.9657	1630.9639	1.1

* = $-H_2O$ (18.0106 Da)

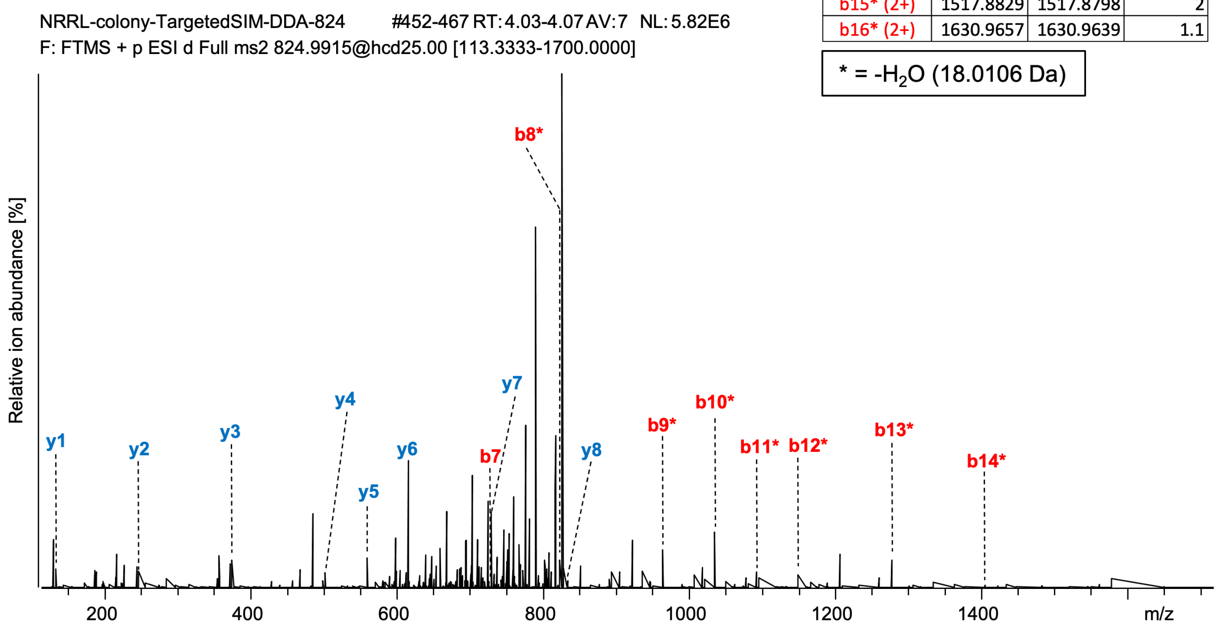

Supplementary Figure 5: Characterization of lassopeptide Lasso-1648 from *Streptomyces rimosus* subsp. *rimosus* NRRL-2660. (a) LCMS analysis of colony extract from *Streptomyces rimosus* subsp. *rimosus* NRRL B-2660 after 6 day growth at 28 ℃ . (b) Detected MS signal of lassopeptide Lasso-1648 from *Streptomyces rimosus* subsp. *rimosus* NRRL B-2660. (c) MS/MS analysis of Lasso-1648.

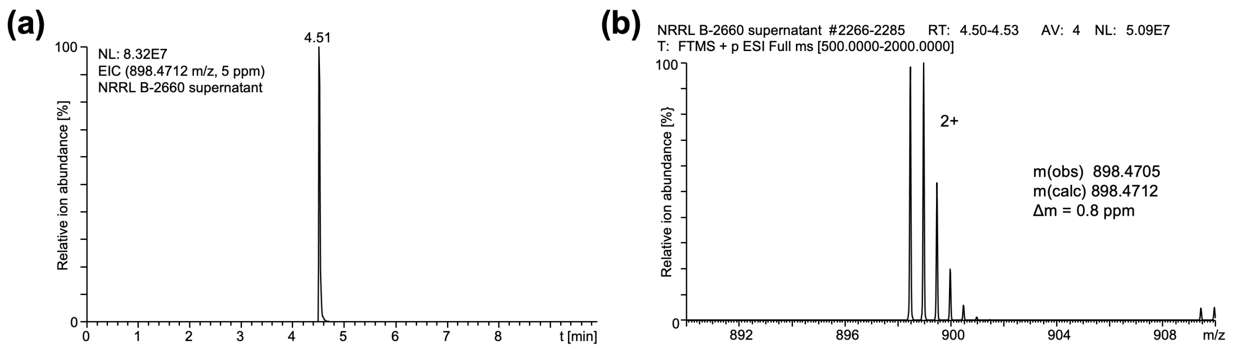

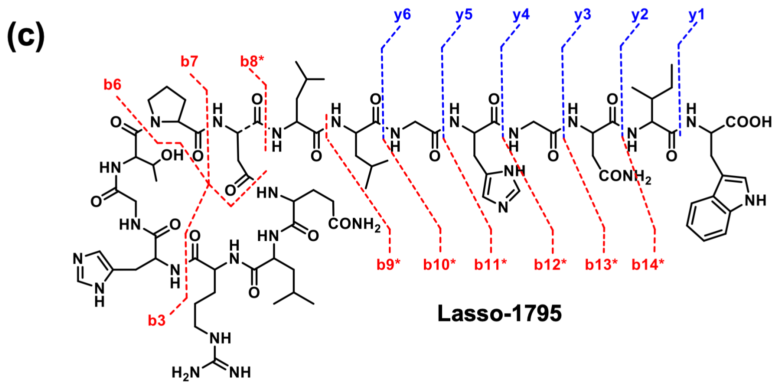

Fragment (z)	m(obs)	m(calc)	Δm [ppm]
y1 (1+)	205.0972	205.0973	-0.5
y2 (1+)	318.18126	318.1815	-0.8
b3 (1+)	398.25107	398.2515	-1.1
y3 (1+)	432.22419	432.2241	0.2
y4 (1+)	489.24565	489.2460	-0.7
y5 (1+)	626.30456	626.3051	-0.9
y6 (1+)	683.3260	683.3276	-2.3
b6 (1+)	693.37913	693.3788	0.5
b7 (1+)	790.43189	790.4355	-4.6
b8* (1+)	887.44823	887.4477	0.6
b9* (1+)	1000.5323	1000.5322	0.1
b10* (1+)	1113.6164	1113.6181	-1.6
b11* (1+)	1170.6378	1170.6390	-1
b12* (1+)	1307.6967	1307.6967	0
b13* (2+)	1364.7182	1364.7155	2
b14* (2+)	1478.7611	1478.7619	-0.5
b16* (2+)	1777.9245	1777.9259	-0.8

* = $-H_2O$ (18.0106 Da)

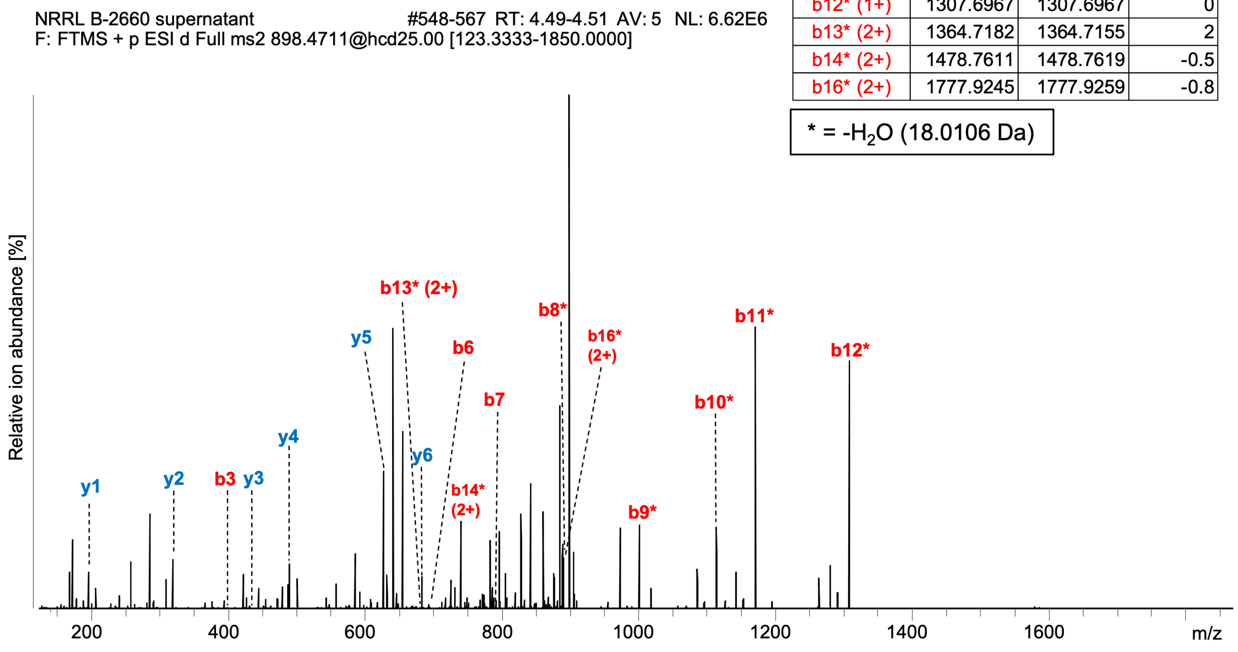

Supplementary Figure 6: Characterization of lassopeptide Lasso-1795 from *Streptomyces rimosus* subsp. *rimosus* NRRL-2660. (a) LCMS analysis of supernatant extract from S*treptomyces rimosus* subsp. *rimosus* NRRL B-2660 after 6 day growth at 28 ℃ . (b) Detected MS signal of lassopeptide Lasso-1795 from *Streptomyces rimosus* subsp. *rimosus* NRRL B-2660. (c) MS/MS analysis of Lasso-1795.

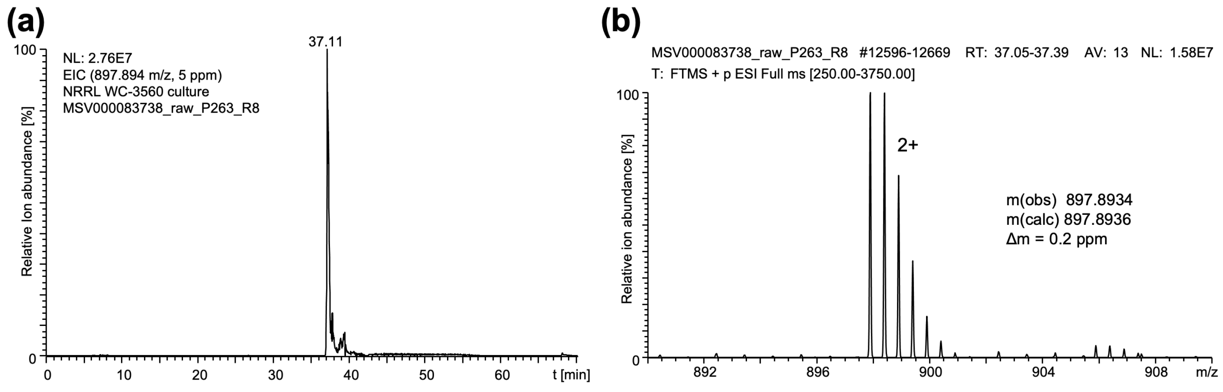

(c)

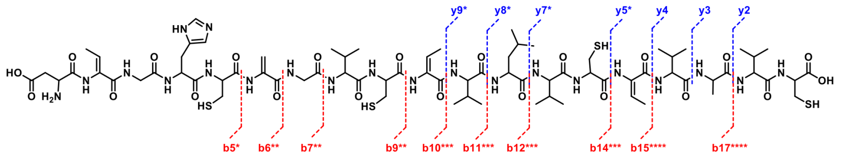

Fragment	m(obs)	m(calc)	Δm [ppm]
y2 (1+)	221.0947	221.0955	-3.6
y3 (1+)	292.1313	292.1326	-4.5
y4 (1+)	391.1996	391.2010	-3.6
y5* (1+)	474.2372	474.2381	-1.9
b5* (1+)	496.1595	496.1609	-2.8
b6** (1+)	565.1804	565.1823	-3.4
b7** (1+)	622.2009	622.2038	-4.6
y7* (1+)	676.3132	676.3157	-3.7
y8* (1+)	789.3970	789.3998	-3.5
b9** (1+)	824.2783	824.2814	-3.7
y9* (1+)	888.4647	888.4682	-3.9
b10*** (1+)	907.3149	907.3185	-3.9
b11*** (1+)	1006.3833	1006.3869	-3.6
b12*** (1+)	1119.4689	1119.4710	-1.8
b14*** (1+)	1321.5461	1321.5486	-1.9
b15**** (1+)	1404.5817	1404.5856	-2.8
b17**** (2+)	1574.6912	1574.6864	3.1

* = $-H_2O$ (18.0106 Da)

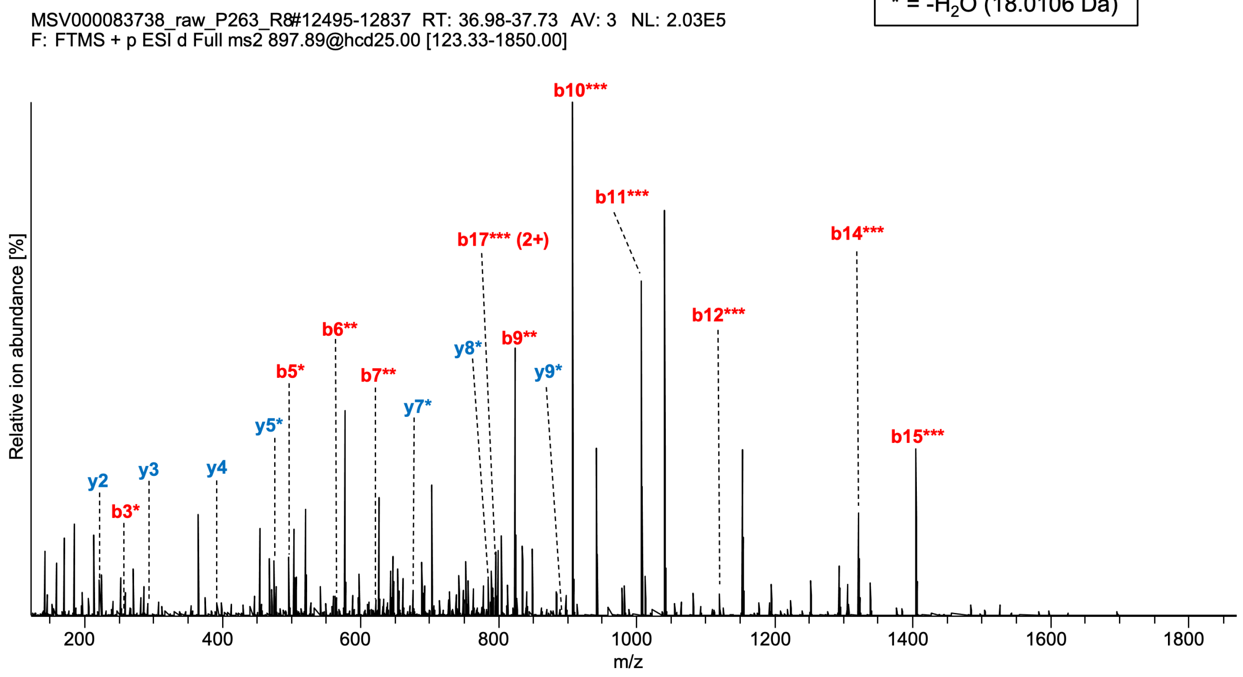

Supplementary Figure 7: Characterization of lanthipeptide Lanthi-1794 from *Streptomyces rimosus* subsp. *rimosus* WC-3904. (a) LCMS analysis of extract from *Streptomyces rimosus* subsp. *rimosus* WC-3904 (MassIVE: MSV000083738_raw_P263_R8). (b) Detected MS signal of lanthipeptide Lanthi-1794 from *Streptomyces rimosus* subsp. *rimosus* WC-3904. (c) MS/MS analysis of Lanthi-1794.

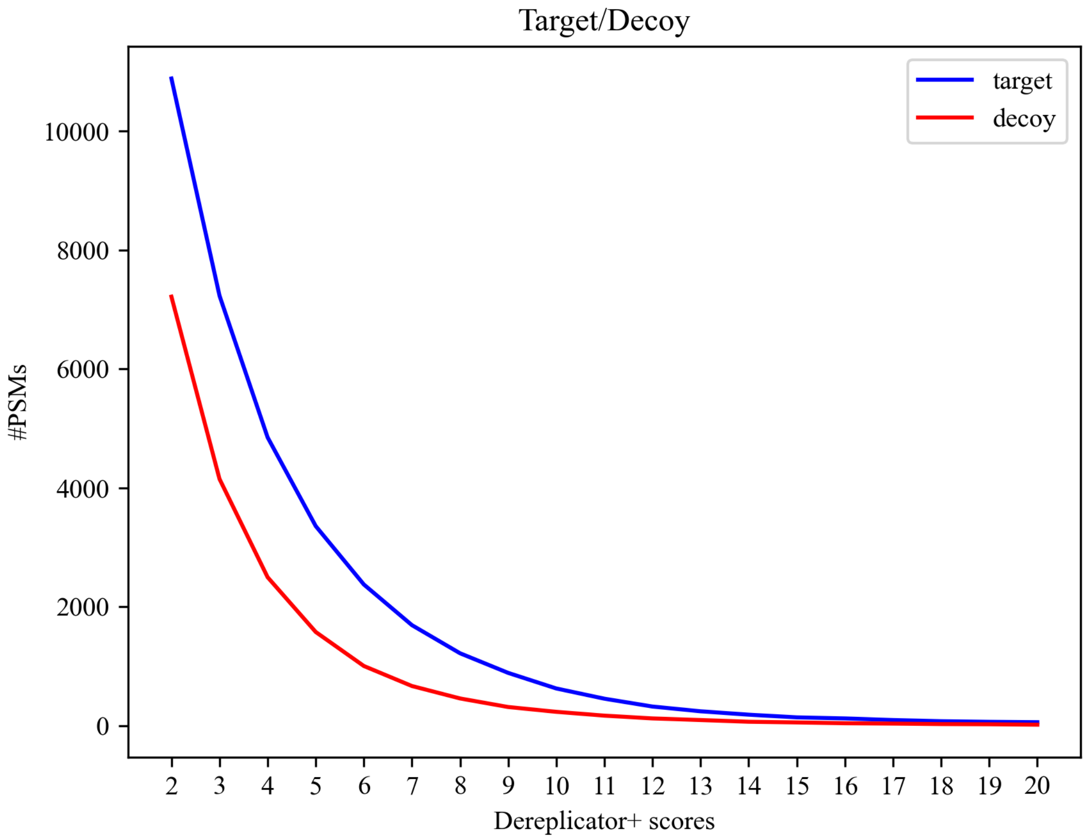

Supplementary Figure 8: Number of peptide-spectrum matches (PSMs) identified at different score thresholds in target and decoy databases, in Dereplicator+ search of RiPPs predicted from PoDP database using seq2ripp, against corresponding mass spectra.

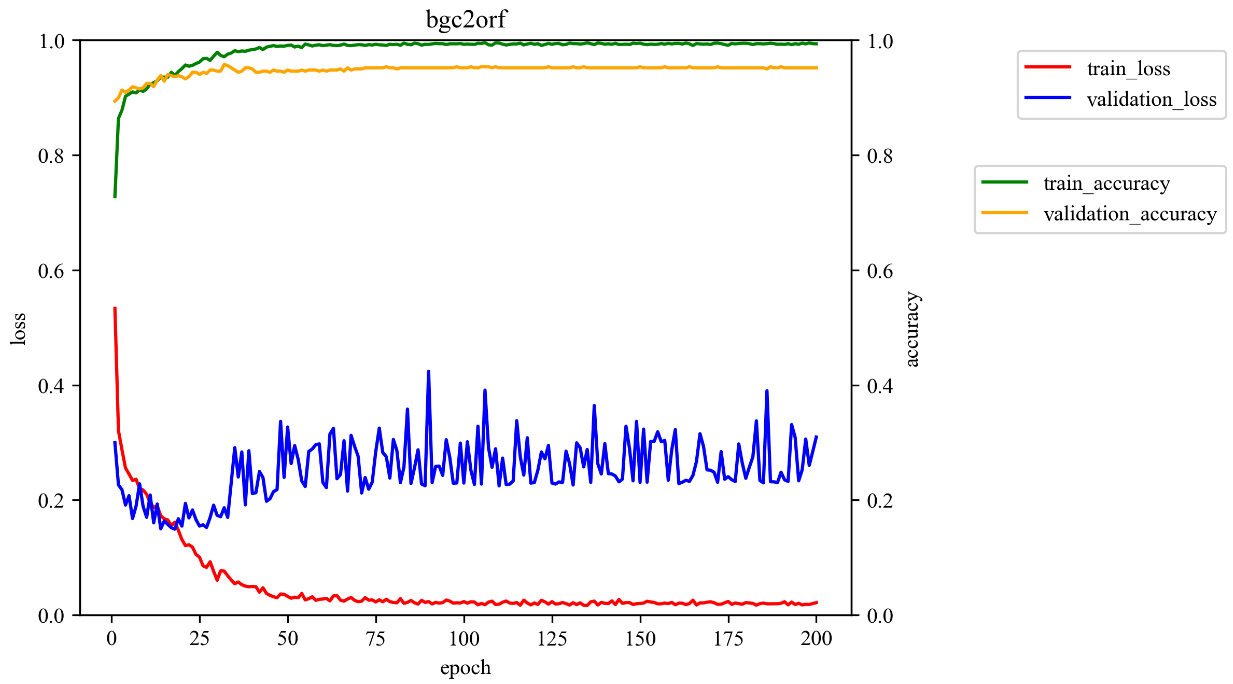

Supplementary Figure 9: Loss/accuracy of bgc2orf. The model in 50th epoch is selected for prediction. After training, the accuracy of correct classification for positive and negative test data are 98.45% and 99.61%, respectively. The test data is unseen by the model during training.

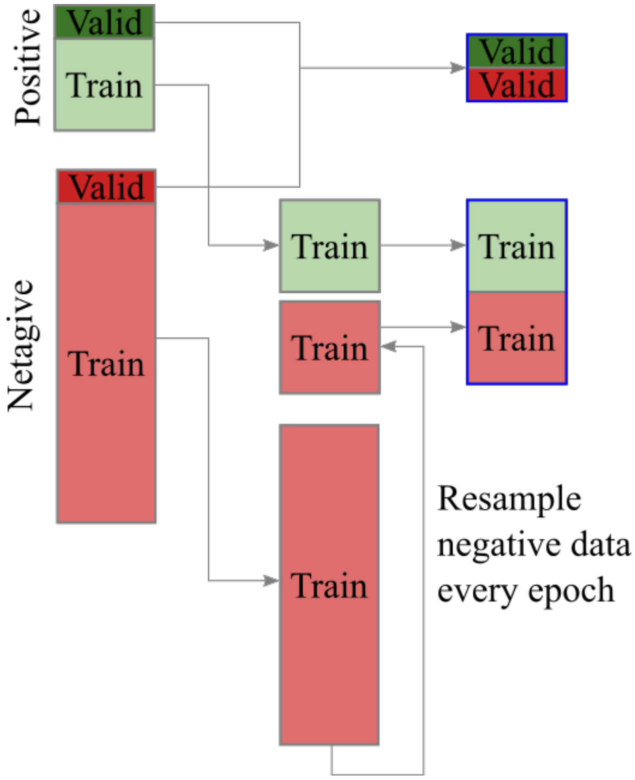

Supplementary Figure 10: Bgc2orf training data sampling. The negative data is seven times more than the positive data (2,726 positive and 19,224 negative). To avoid the model skew to learn the negative dataset, the same amount of negative data as the positive data is re-sampled from the negative data pool every epoch. The validation data is separated before re-sampling and unseen by the model during the training process.

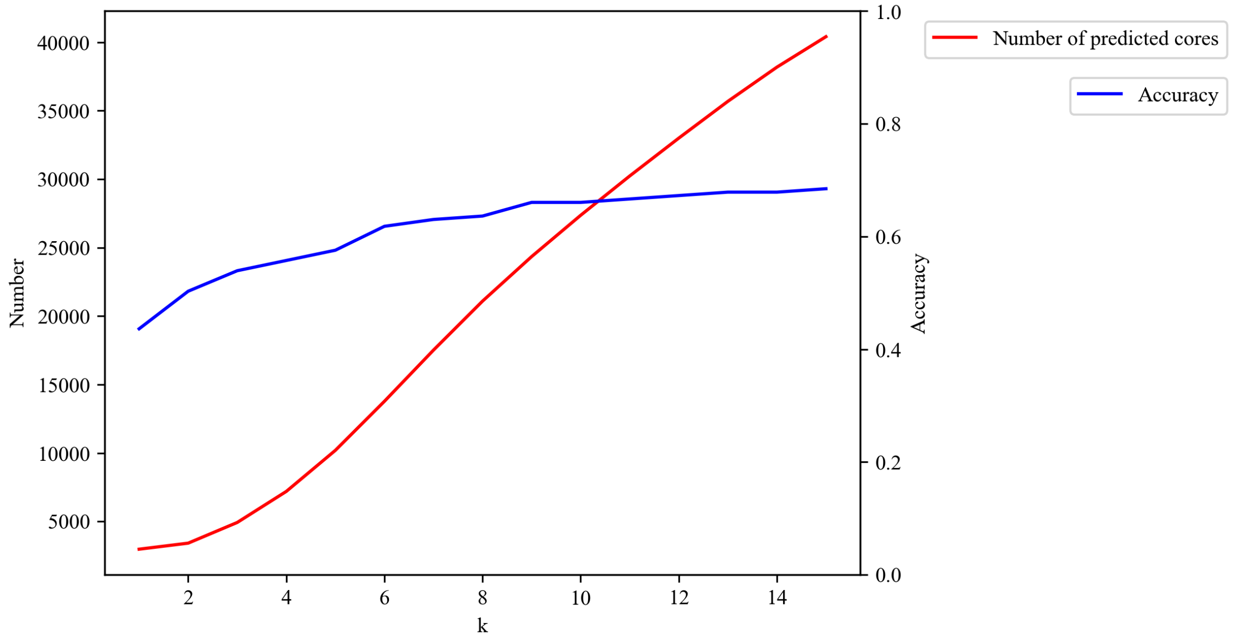

Supplementary Figure 11: Selecting k in the repeat-finder module. X-axis shows k, and Y-axis shows accuracy of prediction (right), and the number of predicted cores per BGC (left), for RiPP BGCs from the MIBiG repository.

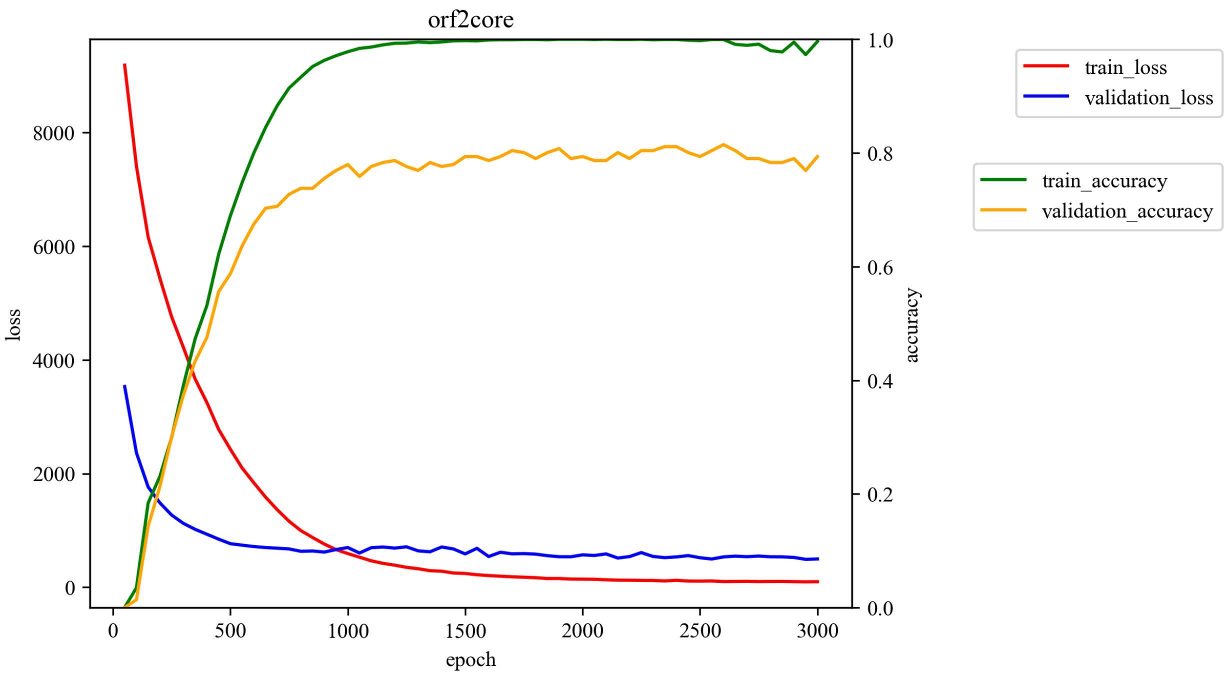

(a)

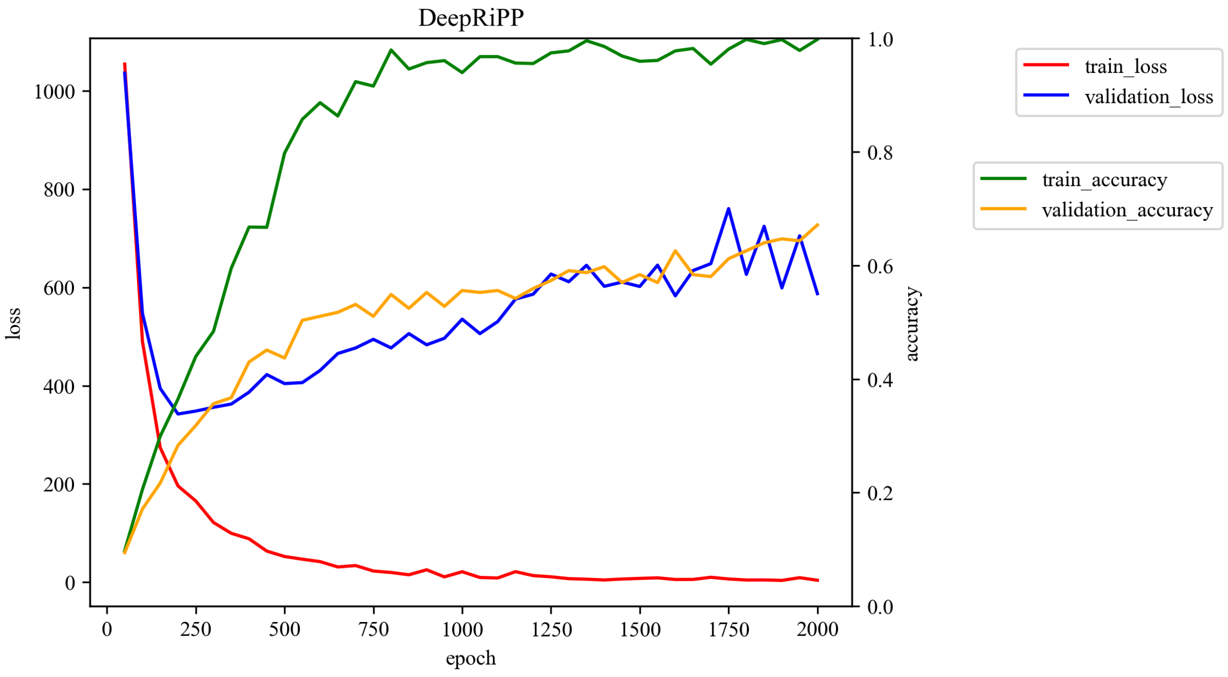

(b)

Supplementary Figure 12: Training loss comparison between orf2core and DeepRiPP. (a) The loss/accuracy during the training of orf2core. The validation accuracy converges to 80%. The model in 1,500 epoch is selected for the prediction. (b) The loss/accuracy during the training of DeepRiPP. The validation accuracy is between 30% to 40% before the model starts overfitting.

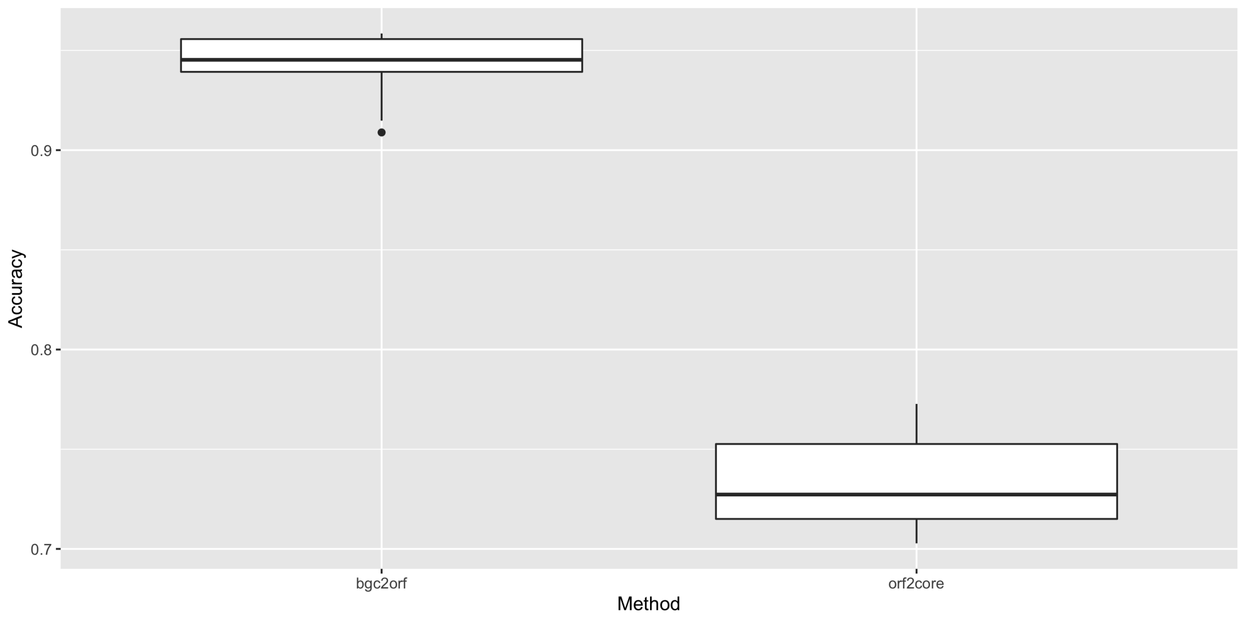

Supplementary Figure 13: Test accuracy distribution of 10-fold cross-validation (CV) in box plot. The box plots illustrate test accuracy distribution for the bgc2orf and orf2core models. The box plots illustrate maximum, third quartile, median, first quartile, and minimum of the data from top to bottom. The results in both 10-fold CV show consistent test accuracies. (n=10)

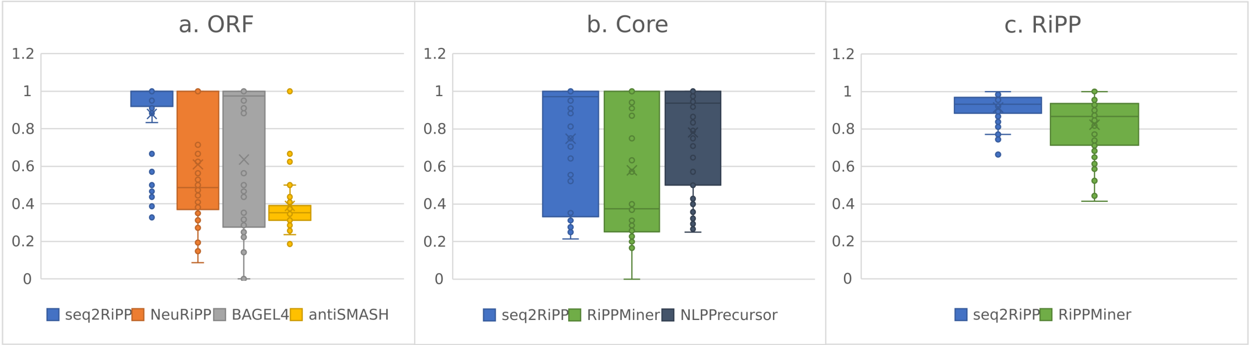

Supplementary Figure 14: A comparison of success rates across different genome mining tools. For N=84 ground truth RiPPs, box and whisker plots represent each tool's success in correctly predicting (a) ORF, (b) core, and (c) RiPP outputs. Each point corresponds to the performance of a tool for a single reference BGC of 84 ground truth RiPPs. The success rate of ORF and core is calculated with Levenshtein distance, and the success rate of RiPP structure is calculated with Tanimoto similarity. The bounds of the box represent the first and third quantiles. The horizontal line and the cross in each box represent the medium and the average. The whiskers represent the 5% and 95% of the score distribution to show the outliers in each task and tool.

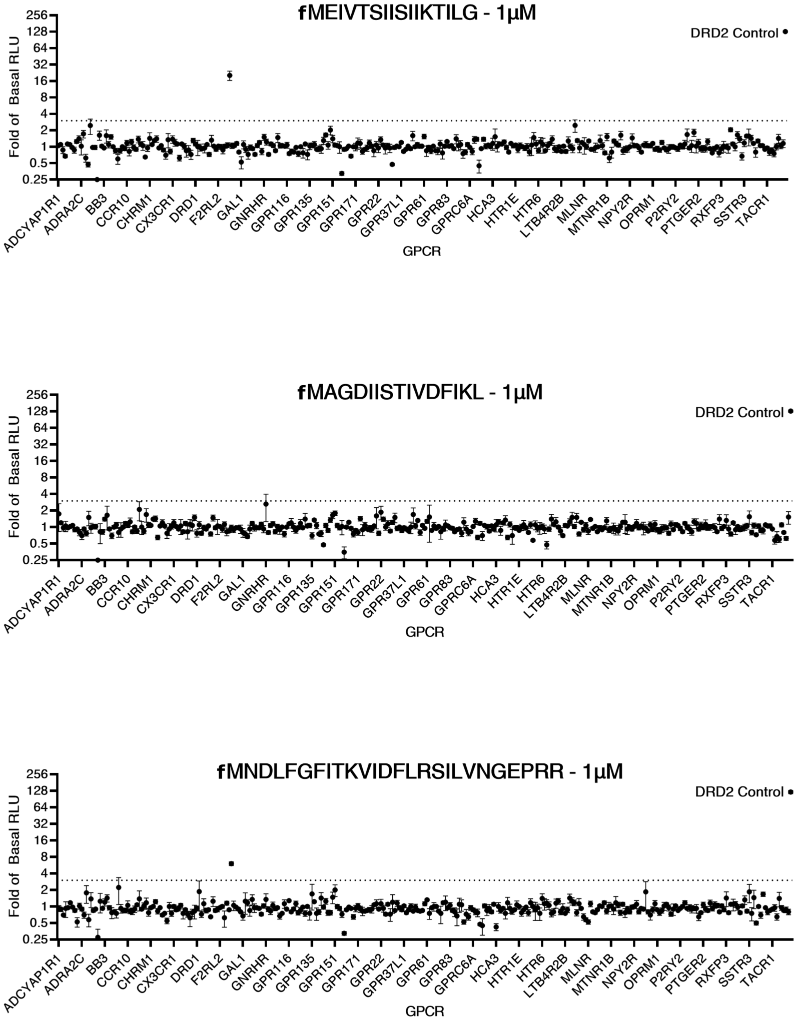

(a)

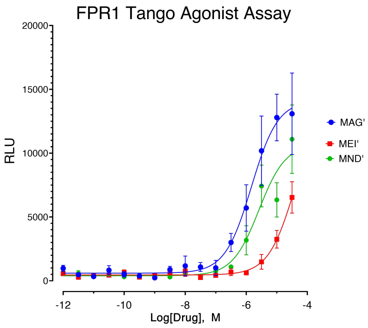

(b)

Supplementary Figure 15: Test bioactivity through PRESTO-Tango GPCRome. (a) PRESTO-Tango GPCRome data obtained using a treatment of 1 μM peptide. 318 human GPCRs were tested and are sorted alphabetically along the X-axis. Prefix "f" indicates the N-methylation. Data are shown as the mean $\pm$ coefficient of variation of replicates. (n=4) (b) FPR1 activity was verified by generating concentration-response curves in PRESTO-Tango. Data are shown as mean $\pm$ standard error means for replicates (n=4).

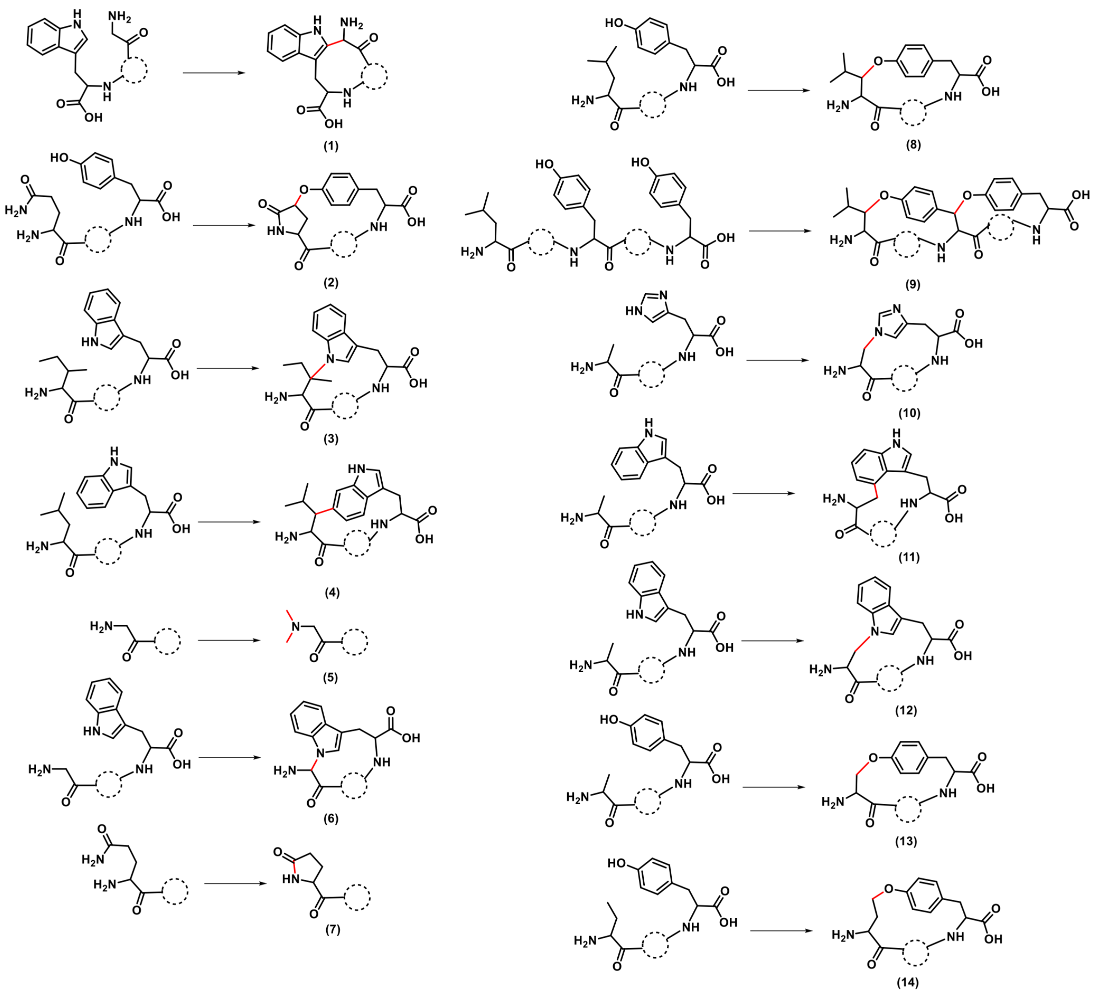

Supplementary Figure 16: The list of plant RiPP modifications used in this study. (1)-(9) are known modifications compiled from Chigumba *et al.* 2022 [1]. The modification sites are highlighted in red. (10)-(14) are novel modifications proposed in this study.

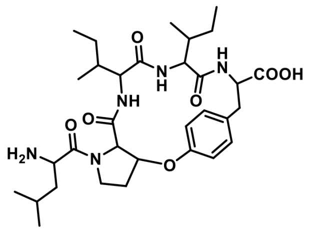

LPIIY (Elaeagnin)

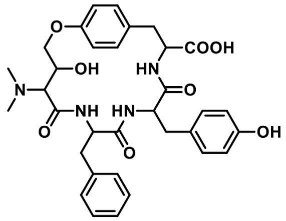

TFYY (Skr-618)

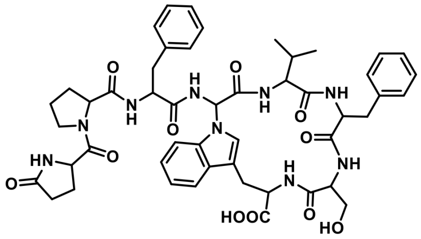

QPFGVFSW (Jdi-931)

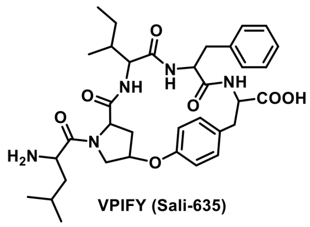

VPIFY (Sali-635)

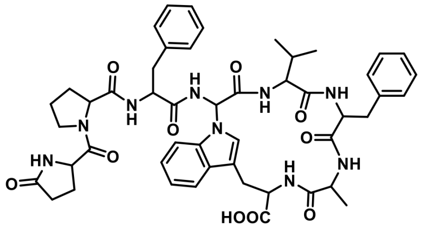

QPFGVFAW (Jdi-931)

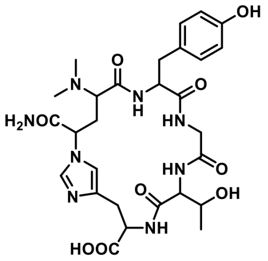

QYGTH (Mtr-630)

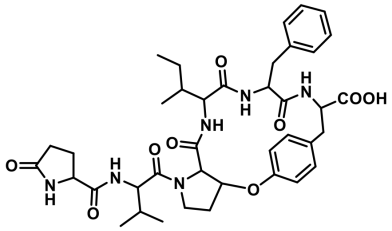

QVPIFY (Sali-746)

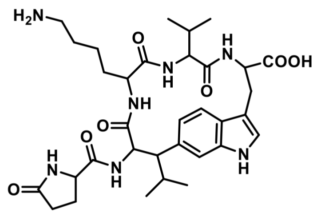

QLKVW (Cca-653)

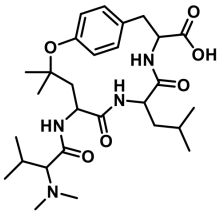

FLLY (Skr-552)

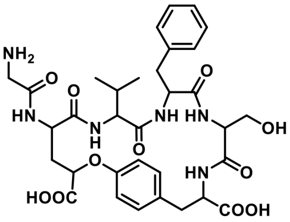

GEVTSY (Skr-652)

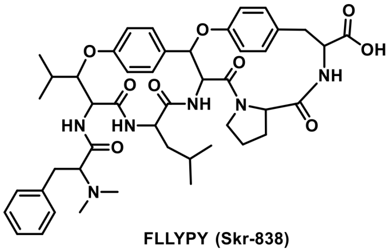

FLLYPY (Skr-838)

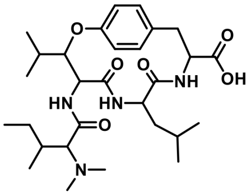

ILLY (Skr-546)

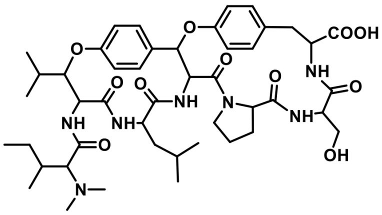

ILLYPSY (Skr-863)

Supplementary Figure 17: Novel plant RiPPs predicted by plant-seq2ripp (Supplementary Table 3).

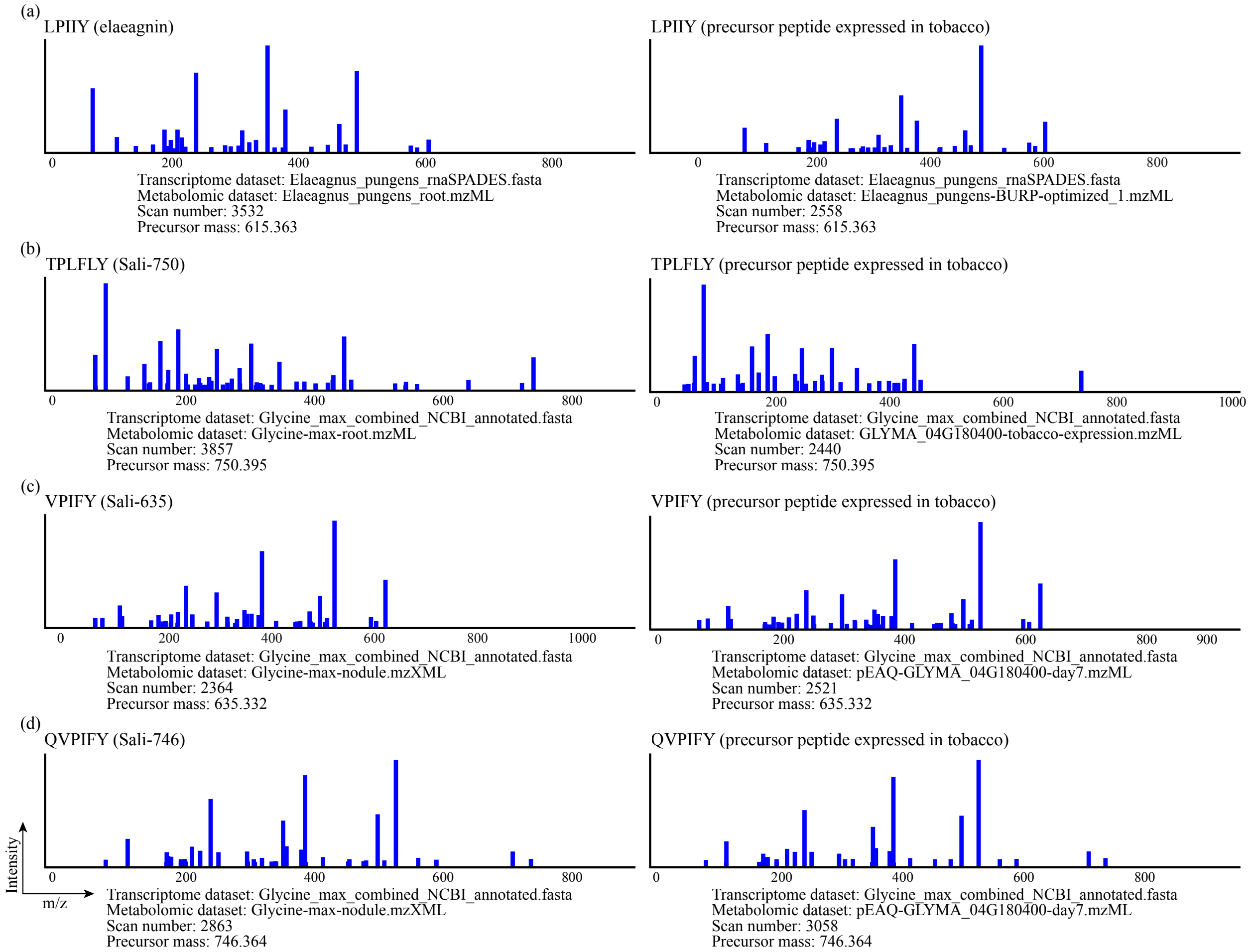

Supplementary Figure 18: Tandem mass spectra of novel plant RiPPs. Tandem mass spectra of four peptides predicted by Seq2RiPP (including elaeagnin) are shown on the left. The predicted core sequences are on the top. For each peptide, the predicted precursor peptide was expressed in tobacco. Then mass spectra were collected from the heterologously expressed precursor peptide, and searched for the presence of the predicted molecules. In each case, a fragment spectrum corresponding to the predicted peptide was found in heterologously expressed samples, shown on the right panel. Included by each spectrum is its transcriptome dataset, metabolomic dataset, scan number, and precursor mass.

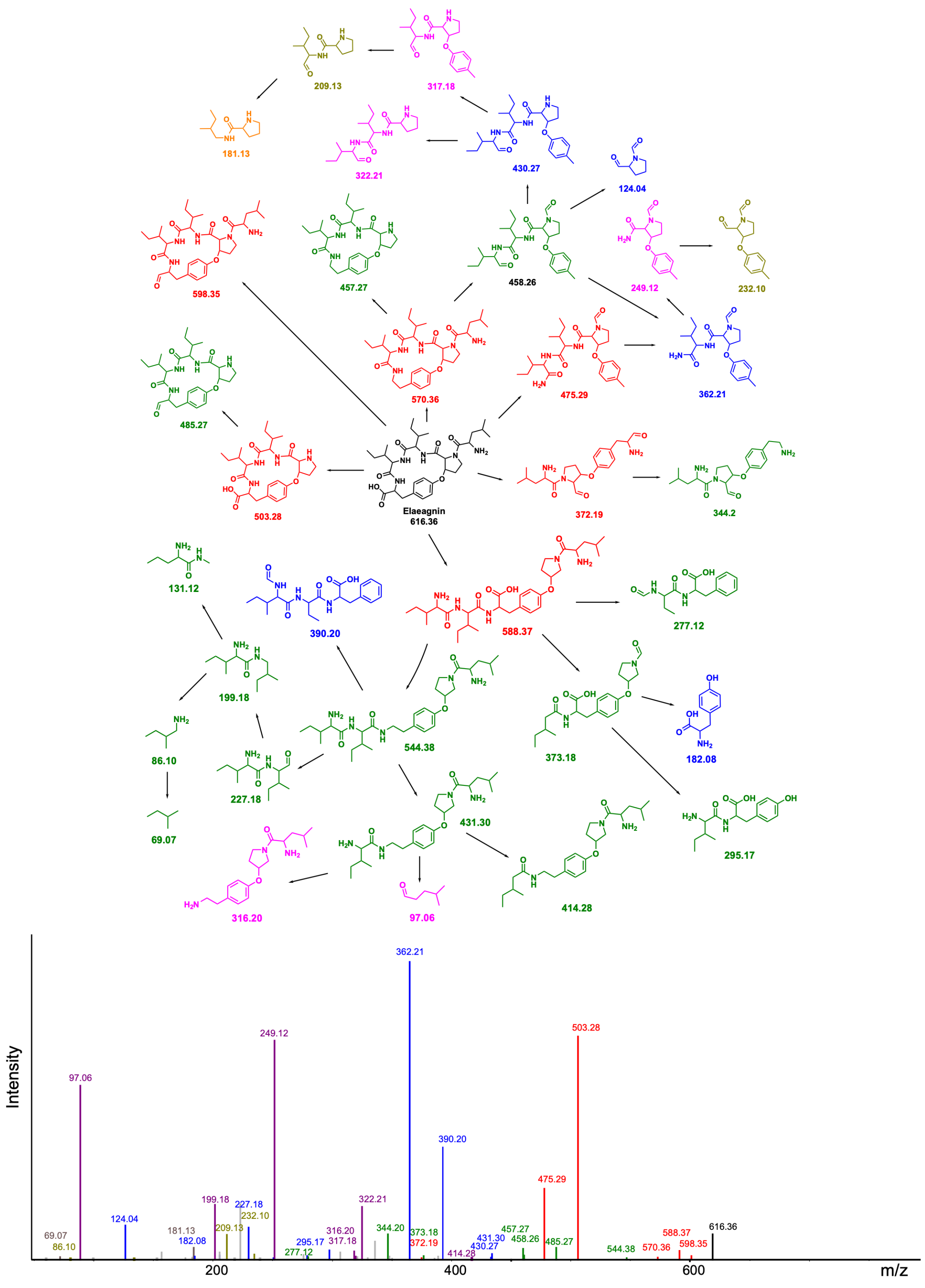

Supplementary Figure 19: Annotation of elaeagnin tandem mass spectrum from Elaeagnus pungens root using Dereplicator+ model.

(a)

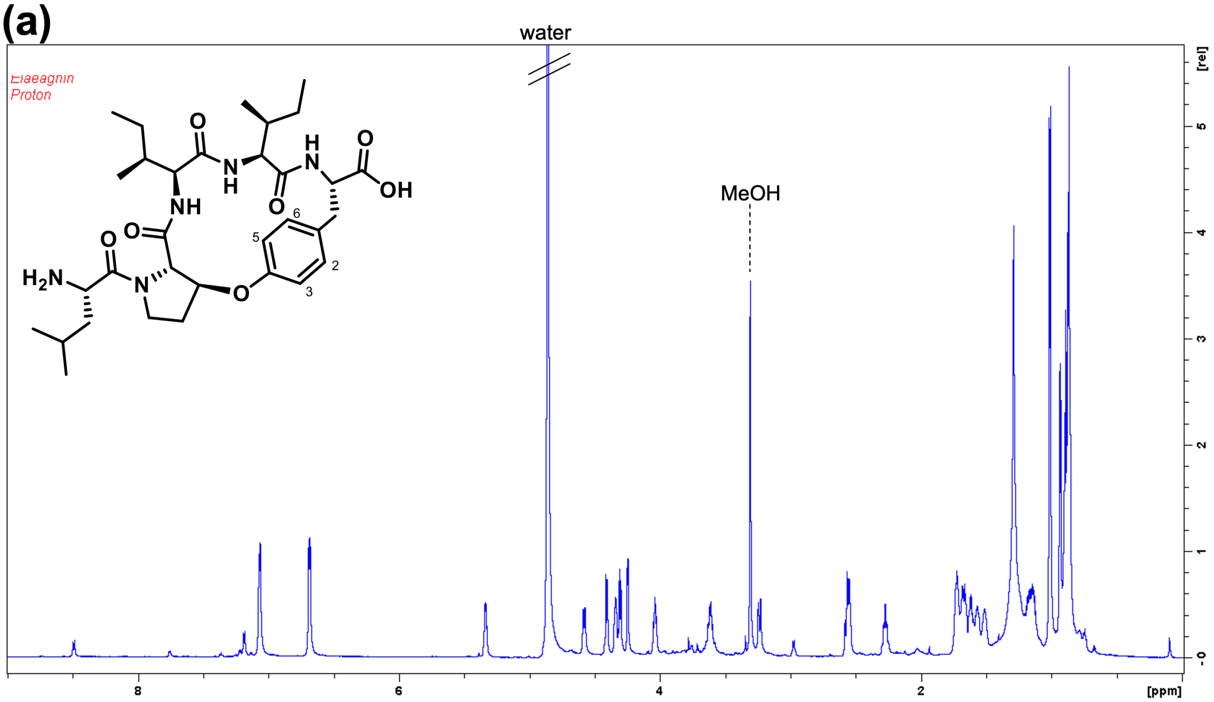
water
Elaeagnin
Proton
MeOH
[rel]
[ppm]
H₂N
NH
OH
6
5
2
3

(b)

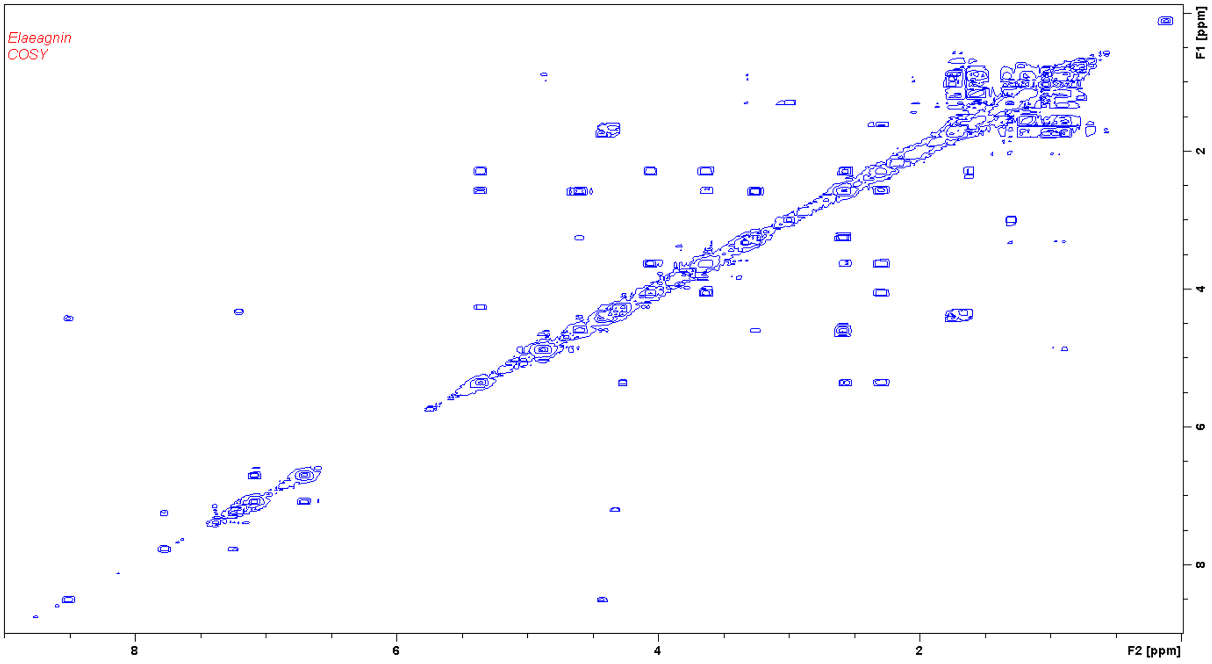
Elaeagnin
COSY
F1 [ppm]
F2 [ppm]

(c)

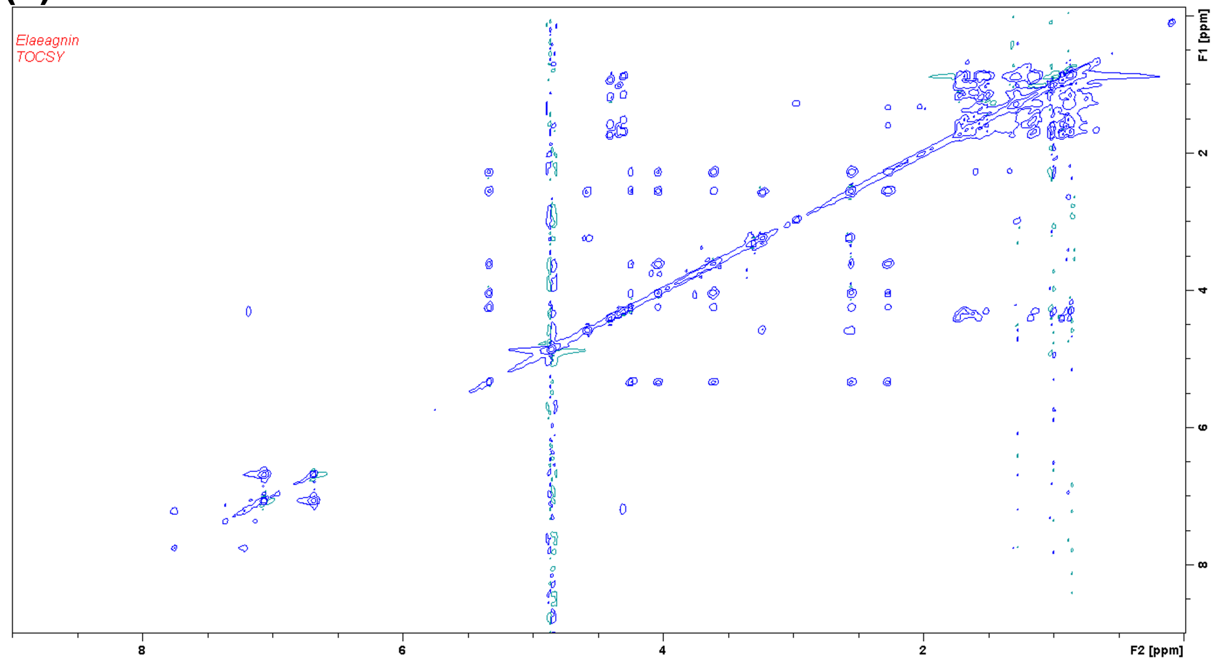
Elaeagnin
TOCSY
F1 [ppm]
F2 [ppm]
8
6
4
2

(d)

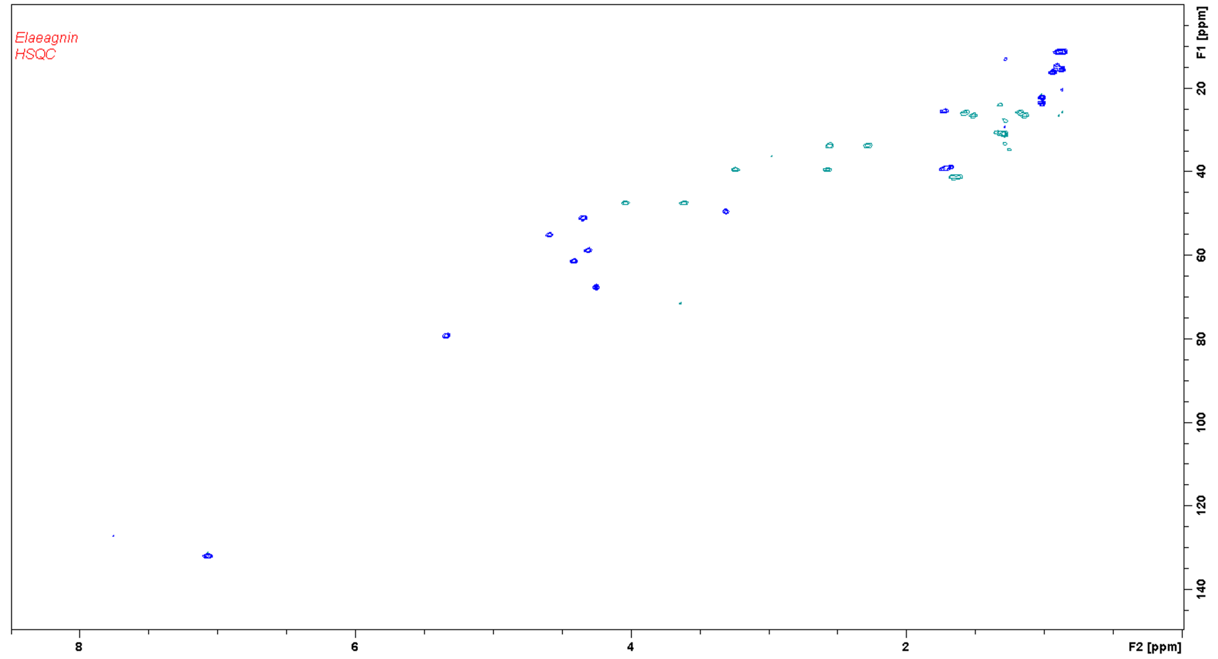
Elaeagnin
HSQC
F1 [ppm]
F2 [ppm]
20
40
60
80
100
120
140
8
6
4
2

(e)

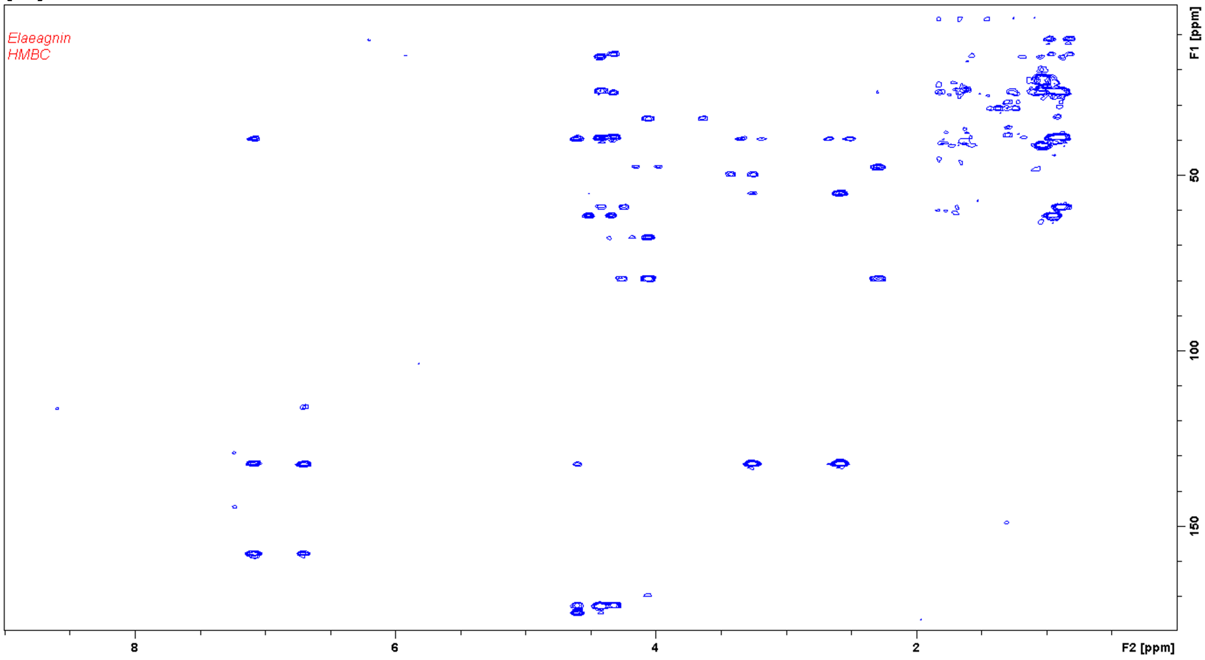
Elaeagnin
HMBC
F1 [ppm]
50
100
150
8
6
4
2
F2 [ppm]

(f)

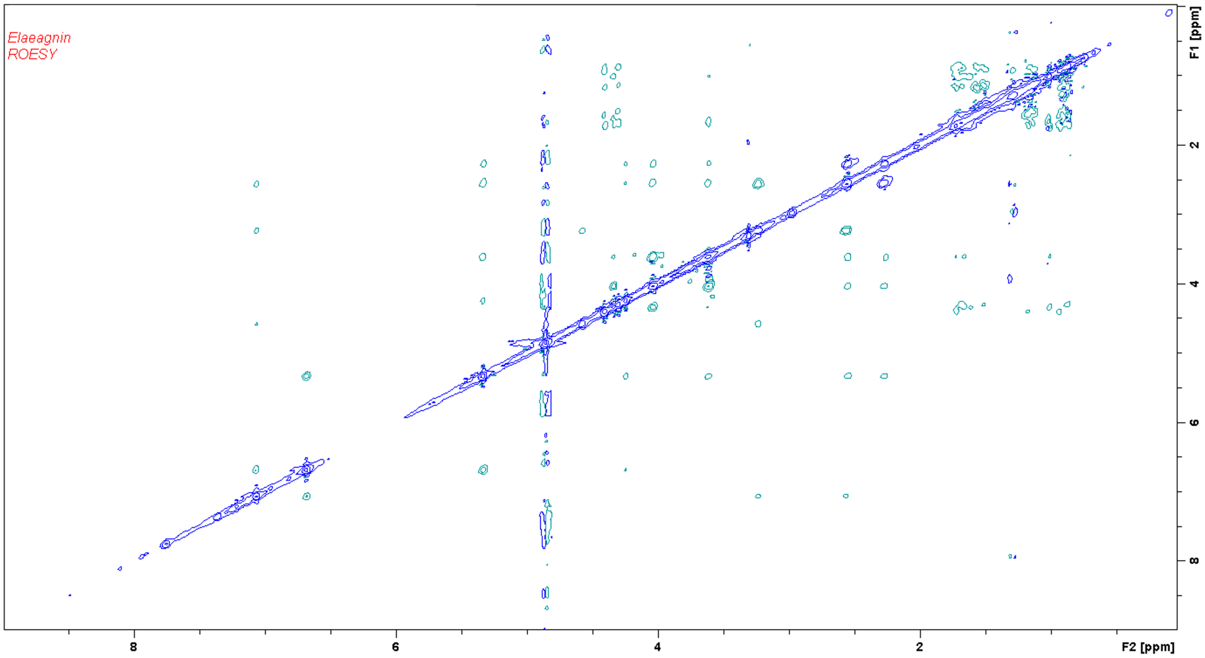
Elaeagnin
ROESY
F1 [ppm]
2
4
6
8
8
6
4
2
F2 [ppm]

(g)

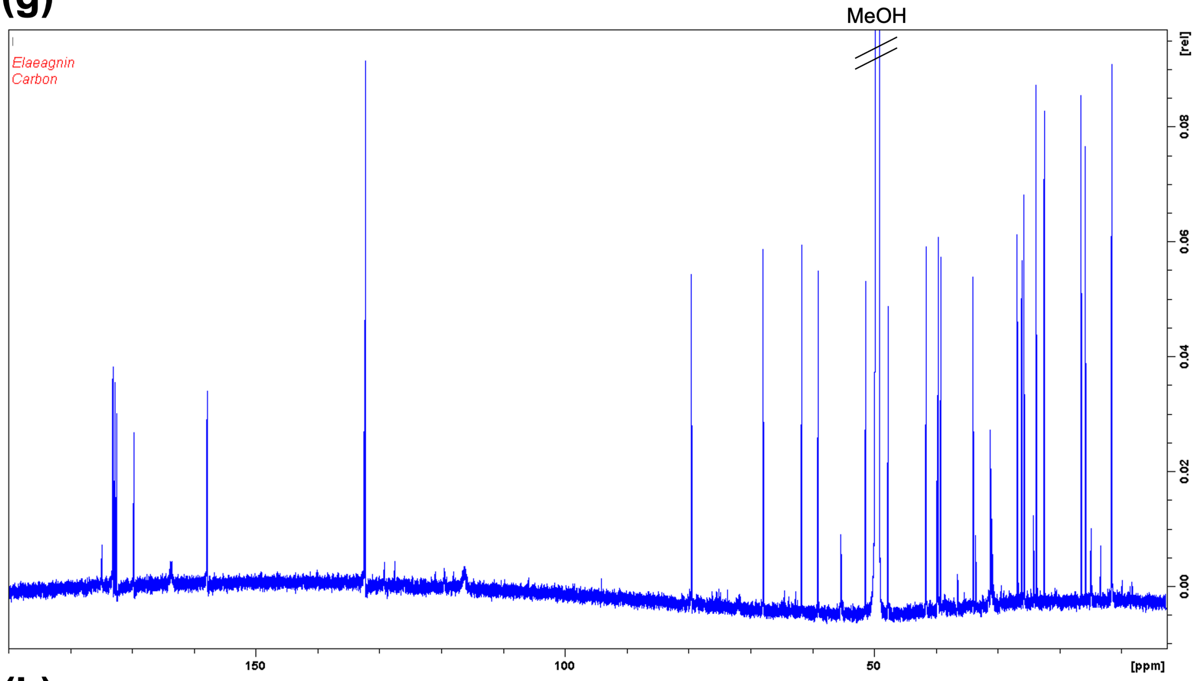

(h)

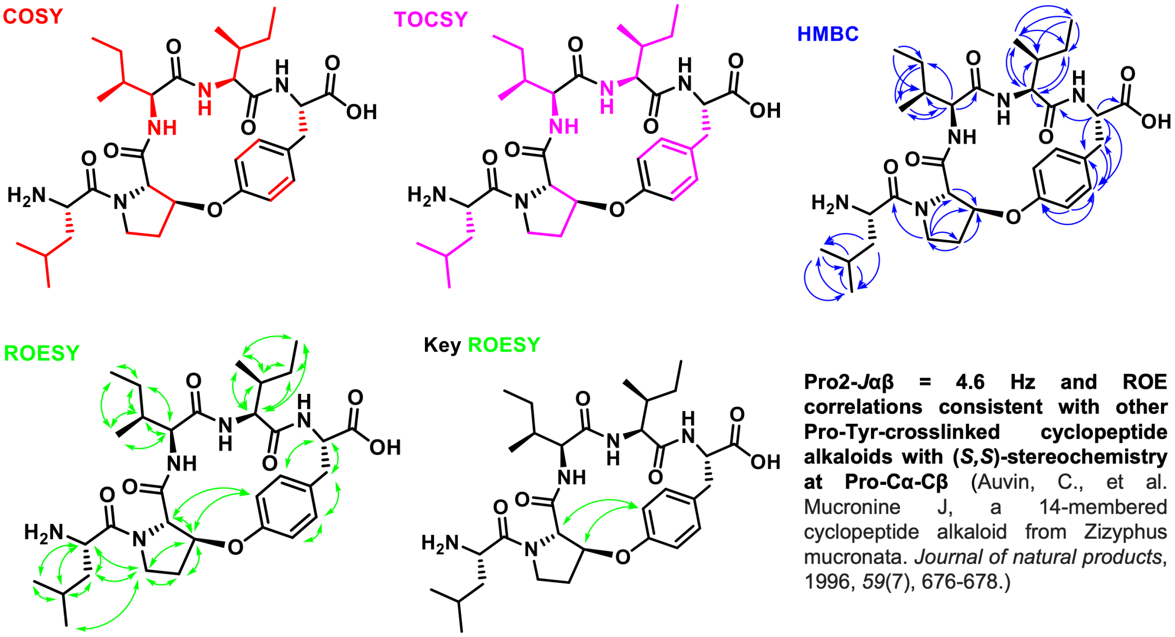

Supplementary Figure 20: Structure elucidation of elaeagnin. (a) ^{1}H NMR spectrum of elaeagnin in MeOD-d4 (800 MHz, 300 K). (b) COSY NMR spectrum of elaeagnin in MeOD-d4 (800 MHz, 300 K). (c) TOCSY NMR spectrum of elaeagnin in MeOD-d4 (800 MHz, 300 K, 120 ms mixing time). (d) HSQC NMR spectrum of elaeagnin in MeOD-d4 (300 K). (e) HMBC NMR spectrum of elaeagnin in MeOD-d4 (300 K). (f) ROESY NMR spectrum of elaeagnin in MeOD-d4 (800 MHz, 300 K). (g) ^{13}C NMR spectrum of elaeagnin in MeOD-d4 (125 MHz, 300 K). (h) NMR correlations of elaeagnin.

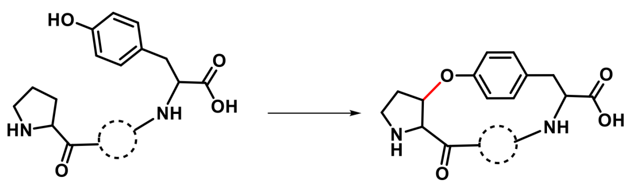

Supplementary Figure 21: A novel post-translational modification with a crosslink between the hydroxyl group of the tyrosine and the β-carbon of the proline.

(a)

>EpuBURP (*Elaeagnus pungens*, ERR2040413, SPAdes 3.13)
MELRLLFLFFLVILHLAGVSSQFDDSHNLHNEGEEAFPNVDKLPTQPAGGVPEKFGKDLLNHMAQSGLFSGGVSEKFGKNLLNHMAQPDIL
PIIYGGGAHENFGKDLLNHMAQPDLLPIIYGGGAPEKIEKDLLNHKAQSDLFSGGASEKFGKDLLNHMAQPDMLPIIYGGGAHENFGKDLL
NHMAQPDLLPIIYGGGAPEKIEKDLLNHKAQSDLFSGGASEKFGKNLLNHMAQPNMLPIIYGGGAHENFGKNLLNHMAQPDMLPIIYGGGA
PMKDSKLMKGNVASFFLENDLLLGKTMKLHFTNPITGAKFLPLDIAKSIPFASNKLPEILNRFNIEPKSSDVEIVKQTISLCEGQKSIETE
HKYCATSLESLIDFSRSKLGEDIKIYSTEVDEEIKQDYKIIKESIVKLGDKSVVCHKLNYMYAVYYCHHVHATKVYMATLEGENGVKENAI
AACHADTKGWNPKHLAFQLLNIKPGTDTICHFLSSDTLVLVPQKDKDVSY

>EpuBURP
ATGGAATTACGTCTCCTTTTCCTTTTCTTTCTCGTCATTCTGCATCTTGCAGGGGTGAGCAGCCAGTTTGATGACAGTCACAACCTTCACAACGAAGGAGAGG
AAGCCTTTCCAAATGTTGACAAGCTCCCAACACAACCAGCTGGTGGTGTCCCTGAAAAATTTGGAAAAGATCTTTTGAATCACATGGCTCAATCTGGCTTATT
TAGTGGTGGTGTCTCTGAAAAATTTGGAAAAAAATCTTTTGAATCATATGGCTCAACCTGACATACTCCCTATAATTTATGGTGGAGGTGCCCATGAAAATTTT
GGAAAAGATCTTTTGAATCACATGGCTCAACCTGACTTATTGCCTATAATTTATGGTGGTGGTGCCCCTGAAAAAATTGAAAAAGATCTTTTGAATCACAAGG
CTCAATCTGACTTATTTAGTGGTGGTGCCTCTGAAAAATTTGGAAAAGATCTTTTGAATCATATGGCTCAACCTGACATGCTCCCTATAATTTATGGTGGAGG
TGCCCATGAAAATTTTGGAAAAGATCTTTTGAATCACATGGCTCAACCTGACTTATTGCCTATAATTTATGGTGGTGGTGCCCCTGAAAAAATTGAAAAAGAT
CTTTTGAACCACAAGGCTCAATCTGACTTATTTAGTGGTGGTGCCTCTGAAAAATTTGGAAAAAAATCTTTTGAATCATATGGCTCAACCTAACATGCTCCCTA
TAATTTATGGTGGAGGTGCCCATGAAAATTTTGGAAAAAAATCTTTTGAATCACATGGCTCAACCTGACATGCTCCCTATAATTTATGGTGGAGGTGCCCCAAT
GAAAGATTCAAAATTAATGAAGGGTAATGTTGCCTCTTTCTTCTTAGAGAACGATCTACTACTAGGTAAAACAATGAAGTTGCATTTTACAAATCCCATAACT
GGTGCTAAGTTCTTACCTCTTGATATTGCAAAATCCATACCTTTTGCTAGTAACAAGTTACCAGAAATTTTAAACCGTTTCAATATTGAGCCTAAATCATCTG
ATGTAGAAATTGTAAAACAAACTATTAGTTTGTGTGAGGGACAAAAAAGCATTGAAACTGAACATAAGTATTGTGCAACATCACTCGAGTCATTGATCGATTT
TAGTAGGTCAAAACTTGGAGAAGACATAAAGATCTACTCAACAGAGGTGGATGAAGAAATCAAACAAGATTACAAAATCATTAAGGAATCAATTGTTAAGTTA
GGAGACAAATCTGTGGTTTGCCATAAGCTAAATTATATGTACGCTGTATATTATTGTCATCATGTTCATGCTACAAAGGTGTATATGGCTACATTAGAAGGTG
AAAATGGGGTAAAAGAAAATGCAATAGCTGCTTGTCATGCTGATACTAAAGGTTGGAATCCTAAGCATTTAGCTTTTCAACTCCTCAATATCAAGCCTGGAAC
AGATACTATTTGCCACTTCCTTTCAAGTGACACCCTTGTTTTGGTTCCTCAAAAGGACAAGGATGTTAGTTATTGA

(b)

>EpuBURP-truncated (5xLPIIY)
MELRLLFLFFLVILHLAGVSSQFDDSHNLHNEGEEAFPNVDKLPTQPAGGVPEKFGKDLLNHMAQSGLFSGGVSEKFGKNLLNHMAQPDIL
PIIYGGGAHENFGKDLLNHMAQPDLLPIIYGGGAPEKIEKDLLNHKAQSDLFSGGASEKFGKDLLNHMAQPDMLPIIYGGGAHENFGKDLL
NHMAQPDLLPIIYGGGAPMKDSKLMKGNVASFFLENDLLLGKTMKLHFTNPITGAKFLPLDIAKSIPFASNKLPEILNRFNIEPKSSDVEI
VKQTISLCEGQKSIETEHKYCATSLESLIDFSRSKLGEDIKIYSTEVDEEIKQDYKIIKESIVKLGDKSVVCHKLNYMYAVYYCHHVHATK
VYMATLEGENGVKENAIAACHADTKGWNPKHLAFQLLNIKPGTDTICHFLSSDTLVLVPQKDKDVSY

(c)

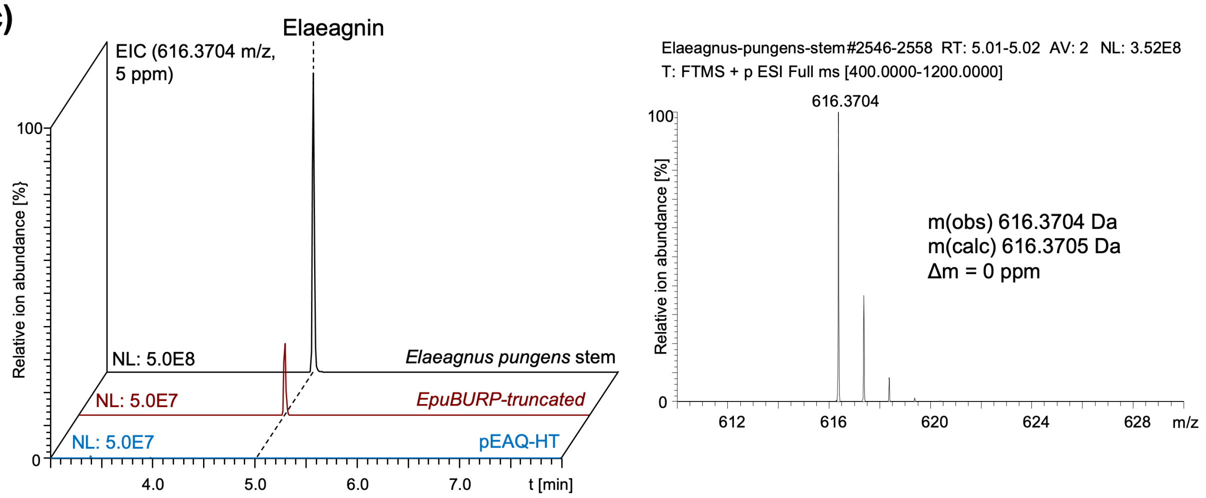

Supplementary Figure 22: Ribosomal biosynthesis of elaeagnin. (a) Candidate elaeagnin precursor peptide EpuBURP derived from SPAdes (v3.13)-assembled transcriptome of *Elaeagnus pungens* (ERR2040413). Grey highlighted text indicates sequence of designed truncated EpuBURP for transient gene expression experiments. (b) Truncated elaeagnin precursor peptide EpuBURP for transient expression in *Nicotiana benthamiana*. (c) LCMS detection of elaeagnin in methanolic extracts of *Elaeagnus pungens* stem and in *N. benthamiana* leaves 6 days after transient expression of truncated EpuBURP via *Agrobacterium tumefaciens* LBA4404-mediated infiltration and pEAQ-HT expression system. Transient expression of EpuBURP and elaeagnin production in *N. benthamiana* were reproducible in triplicate.

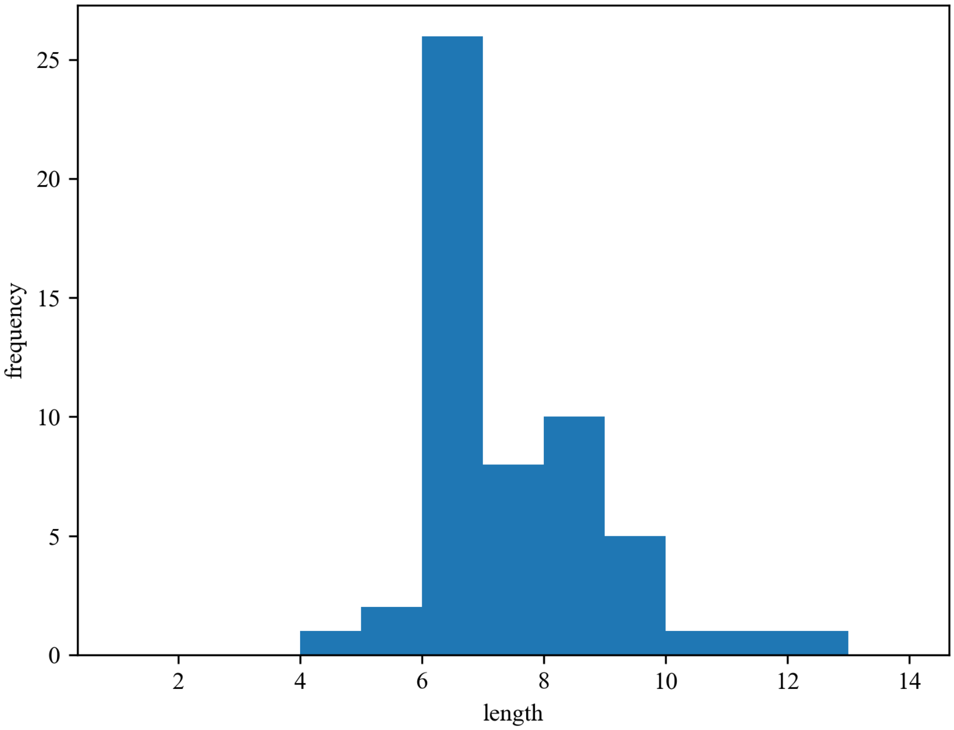

Supplementary Figure 23: Length of cyanobactin cores from in-house cyanobactin database collected from Sivonen et al [8]. The maximum and the minimum length of a core are 4 and 12, respectively. This information is used to set thresholds in the repeat-finder module.

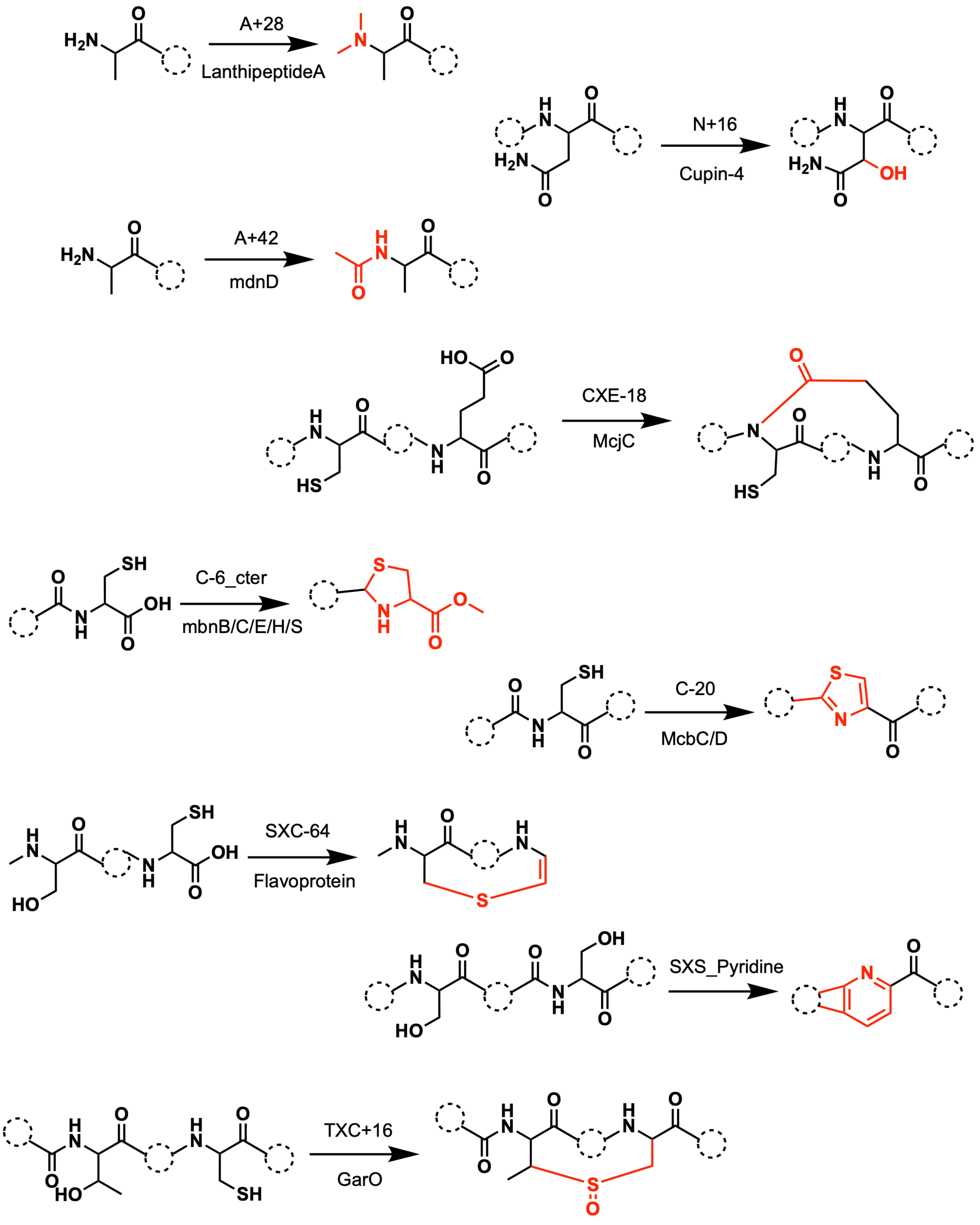
A+28
LanthipeptideA
N+16
Cupin-4
A+42
mdnD
CXE-18
McjC
C-6_cter
mbnB/C/E/H/S
C-20
McbC/D
SXC-64
Flavoprotein
SXS_Pyridine
TXC+16
GarO

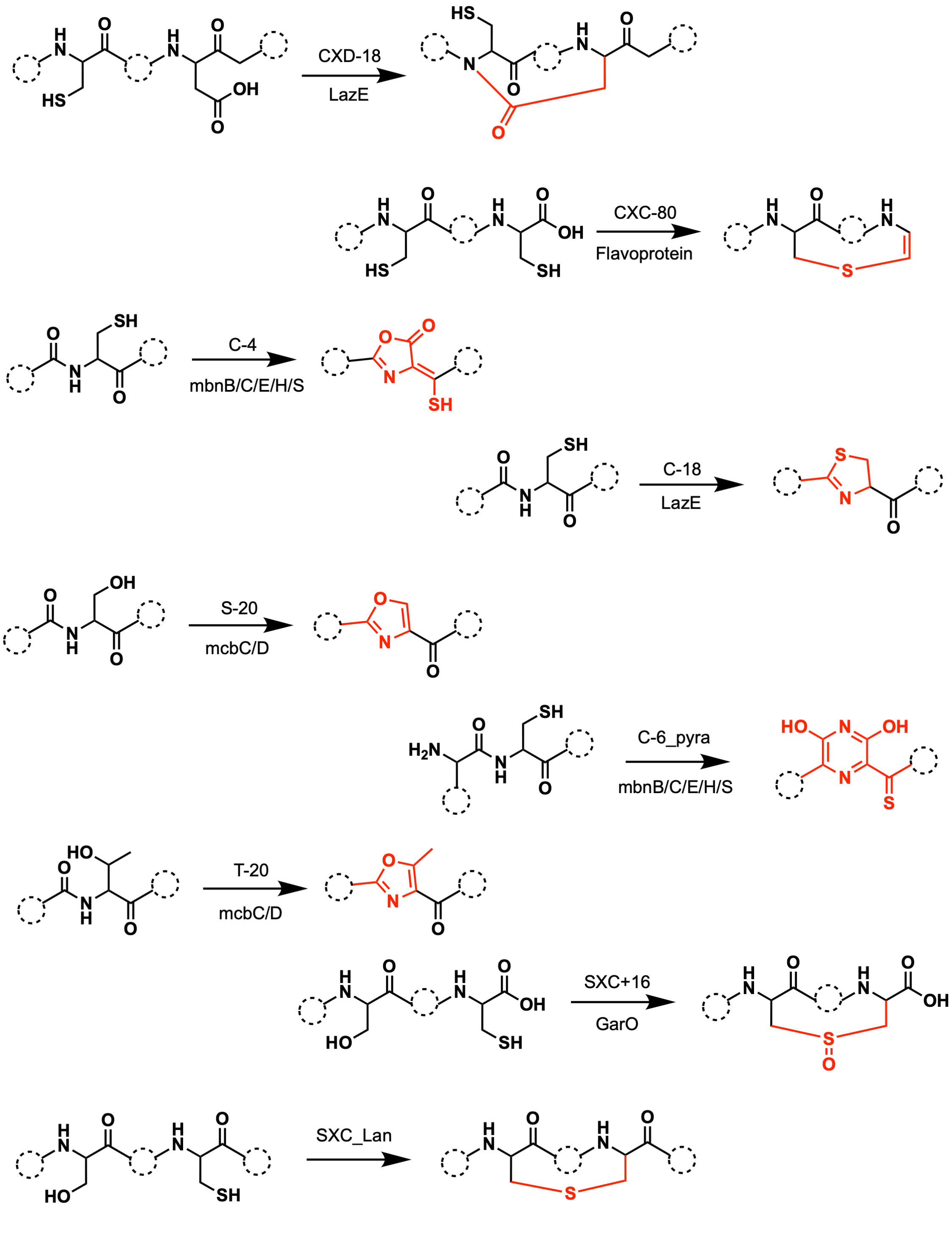
CXD-18
LazE
CXC-80
Flavoprotein
C-4
mbnB/C/E/H/S
C-18
LazE
S-20
mcbC/D
C-6_pyra
mbnB/C/E/H/S
T-20
mcbC/D
SXC+16
GarO
SXC_Lan

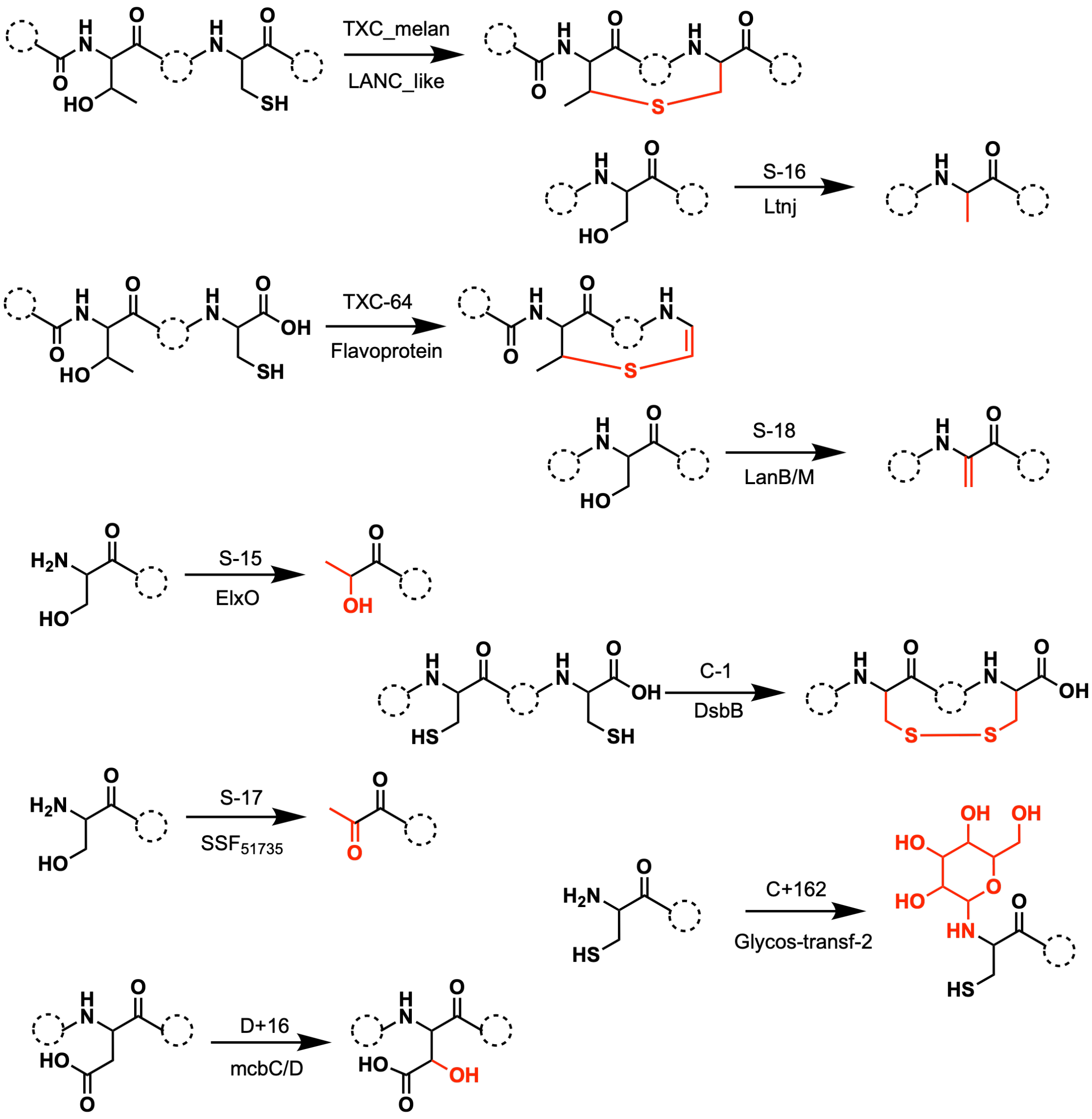
TXC_melan
LANC_like
S-16
Ltnj
TXC-64
Flavoprotein
S-18
LanB/M
S-15
ElxO
C-1
DsbB
S-17
SSF51735
C+162
Glycos-transf-2
D+16
mcbC/D

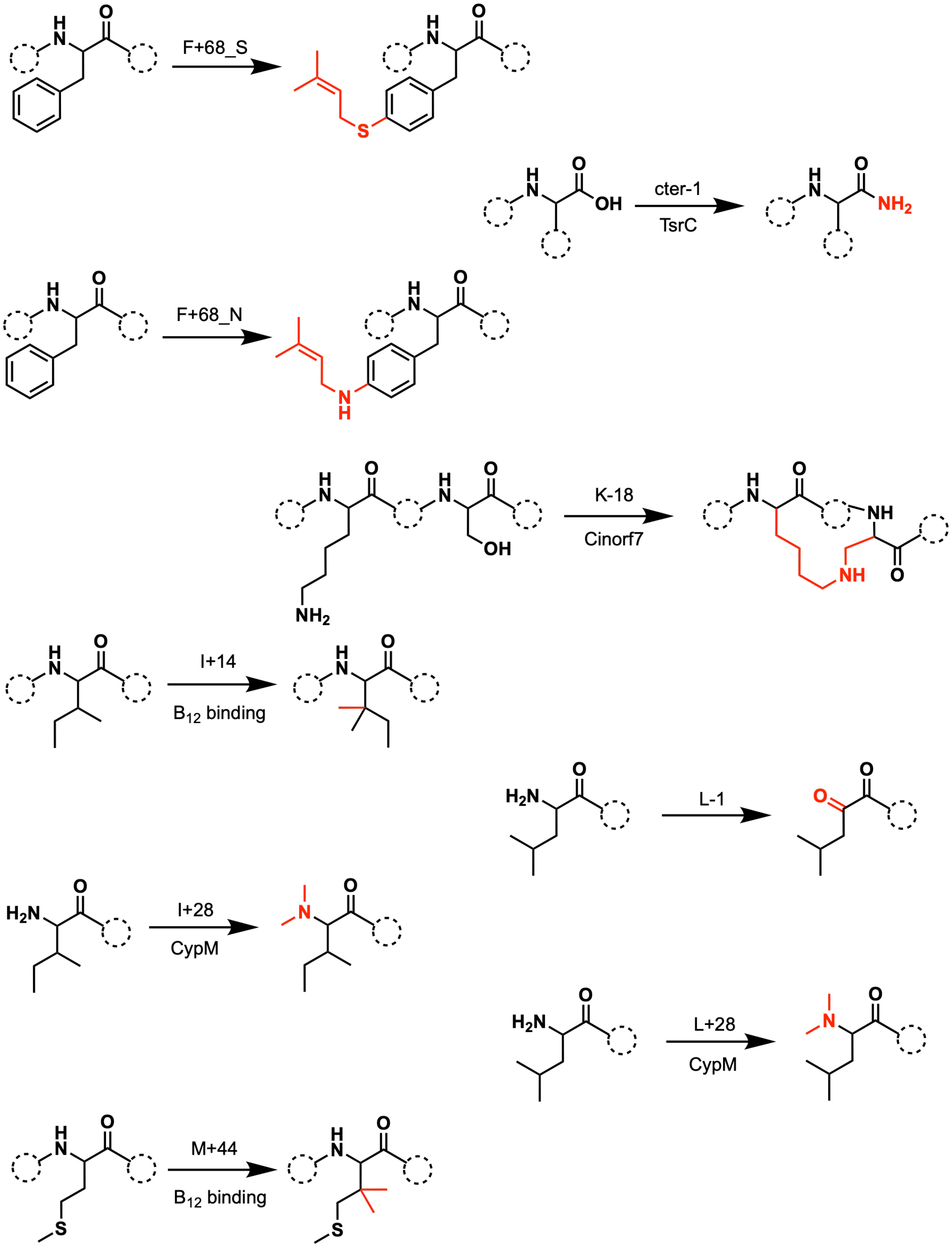
F+68_S
cter-1
TsrC
F+68_N
K-18
Cinorf7
I+14
B12 binding
L-1
I+28
CypM
L+28
CypM
M+44
B12 binding

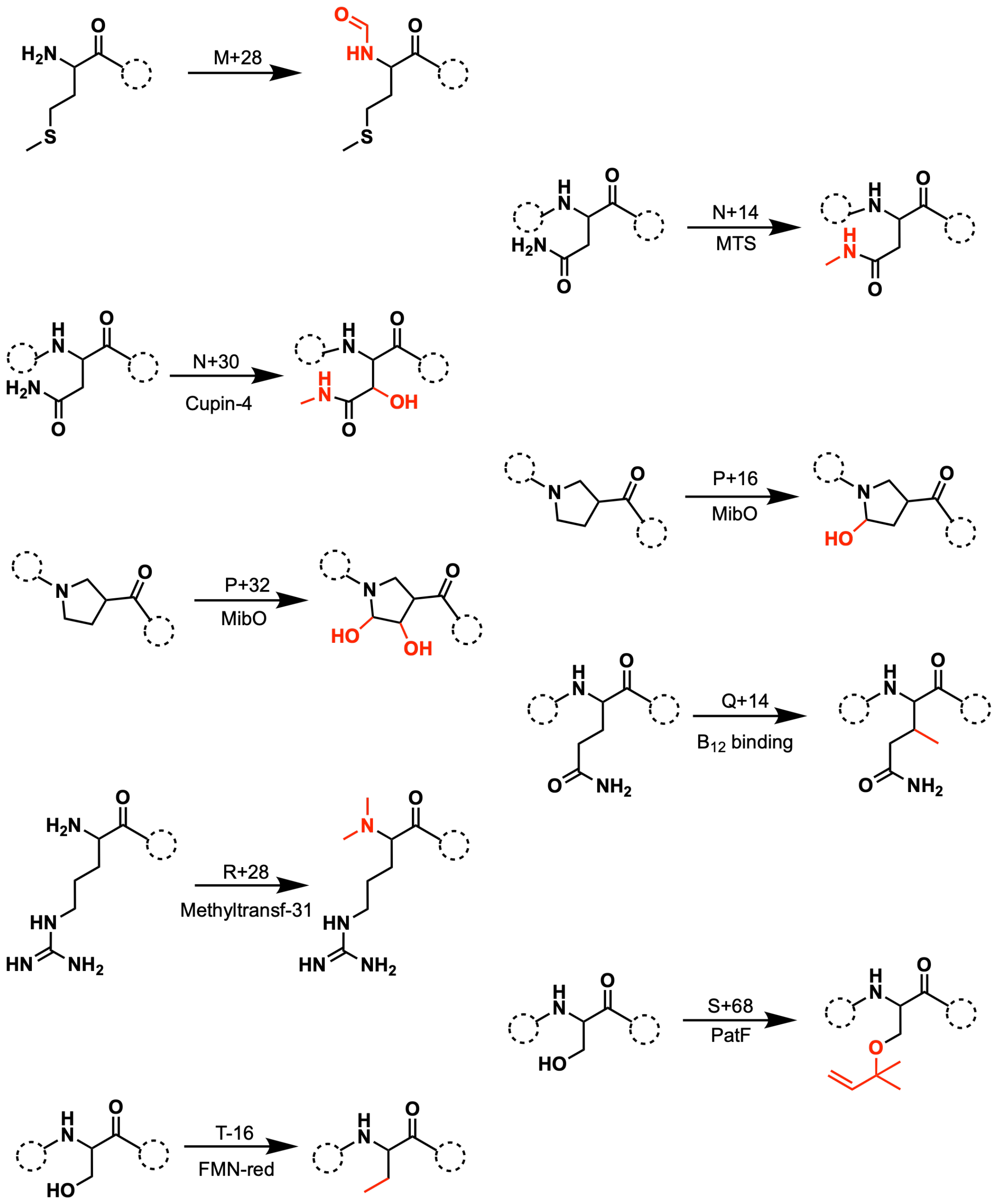
M+28
N+14
MTS
N+30
Cupin-4
P+16
MibO
P+32
MibO
Q+14
B12 binding
R+28
Methyltransf-31
S+68
PatF
T-16
FMN-red

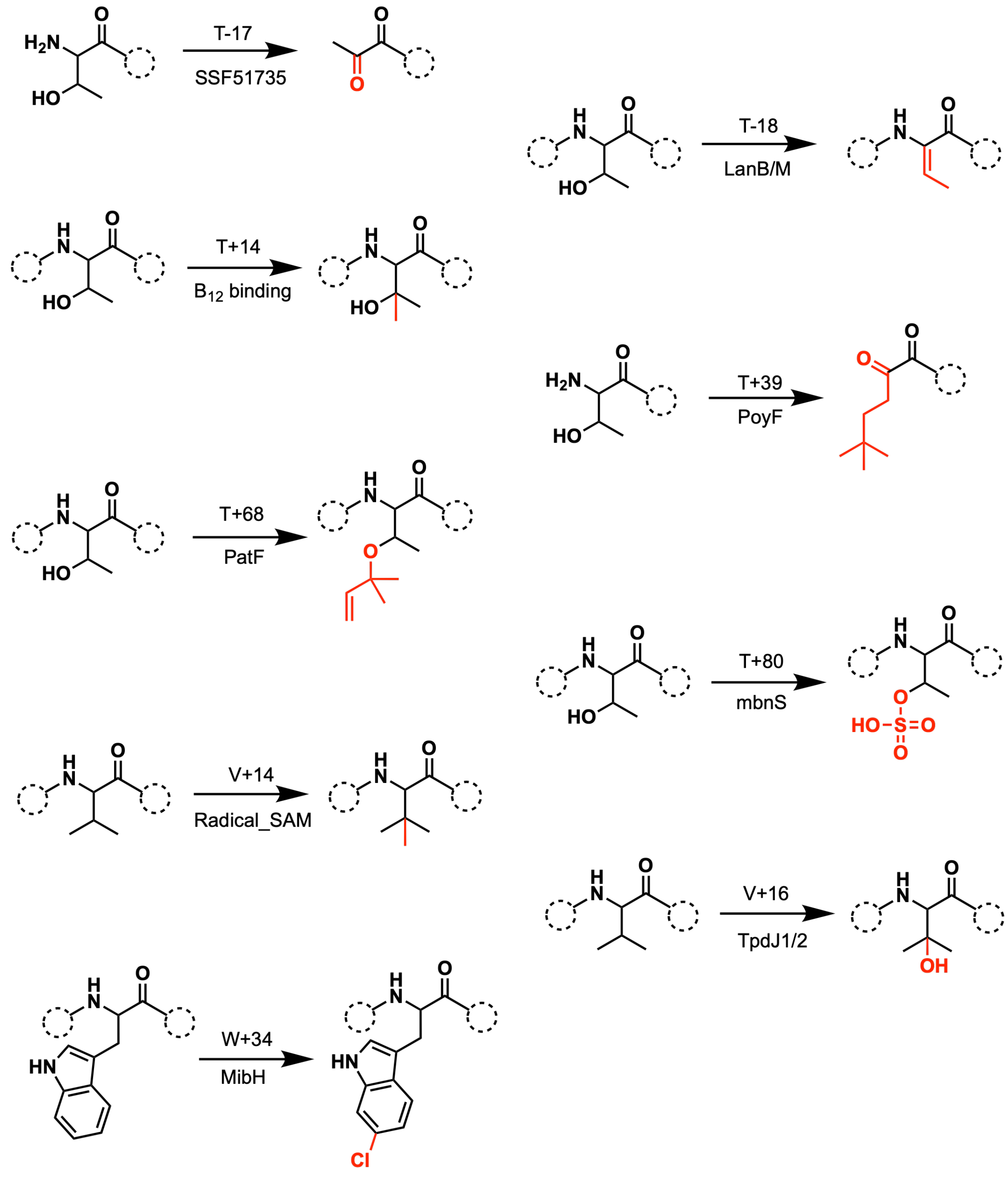
T-17
SSF51735
T-18
LanB/M
T+14
B12 binding
T+39
PoyF
T+68
PatF
T+80
mbnS
V+14
Radical_SAM
V+16
TpdJ1/2
W+34
MibH

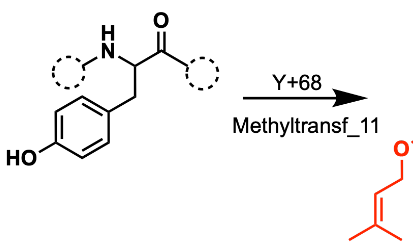
O
H
N
HO
Y+68
Methyltransf_11
O
H
N
O

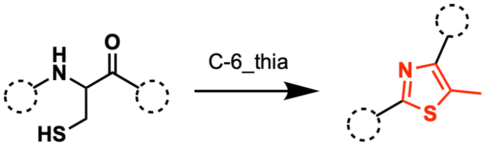
O
H
N
HS
C-6_thia
N
S

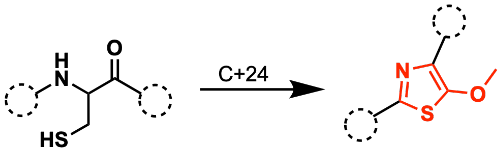
O
H
N
HS
C+24
N
S
O

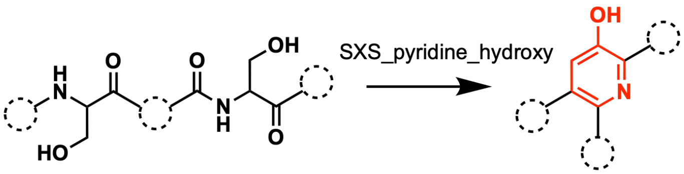
OH
H
N
O
O
N
H
O
HO
SXS_pyridine_hydroxy
OH
N

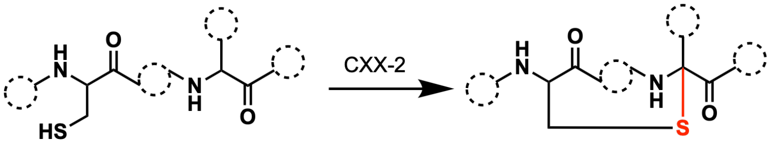
O
H
N
HS
N
H
O
CXX-2
O
H
N
N
H
O
S

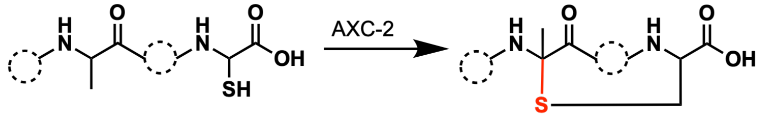
H
N
O
H
N
O
OH
SH
AXC-2
H
N
O
H
N
O
OH
S

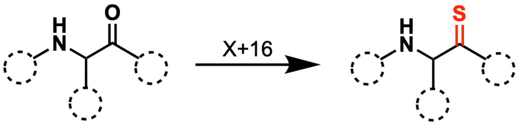
O
H
N
X+16
S
H
N

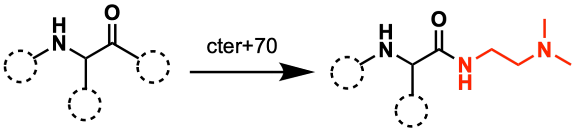
O
H
N
cter+70
H
N
O
N
H
N

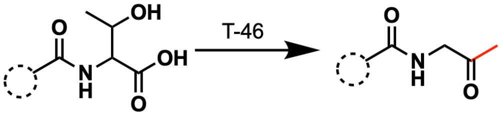
OH
O
N
H
OH
O
T-46
O
N
H
O

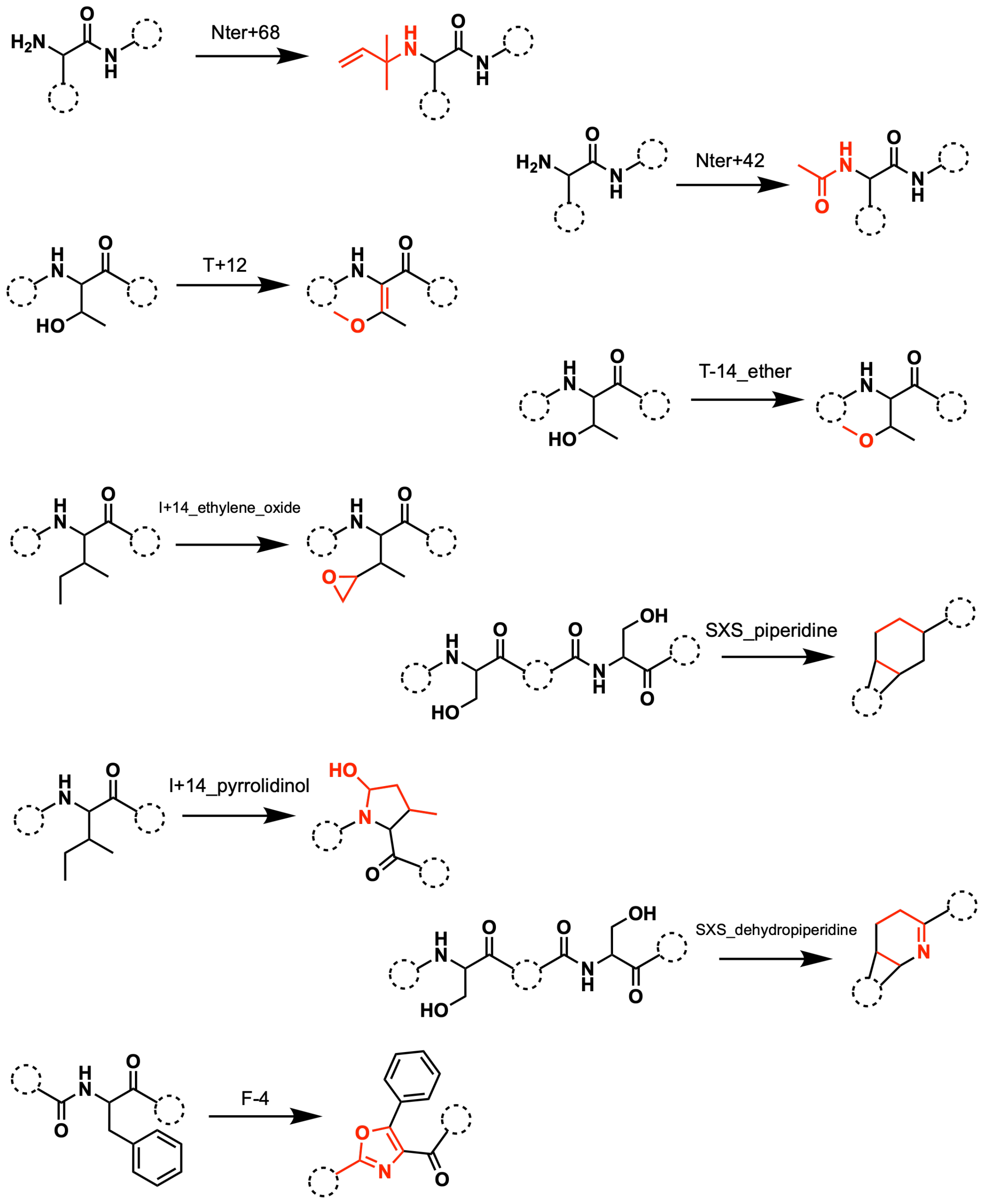
Nter+68
Nter+42
T+12
T-14_ether
I+14_ethylene_oxide
SXS_piperidine
I+14_pyrrolidinol
SXS_dehydropiperidine
F-4

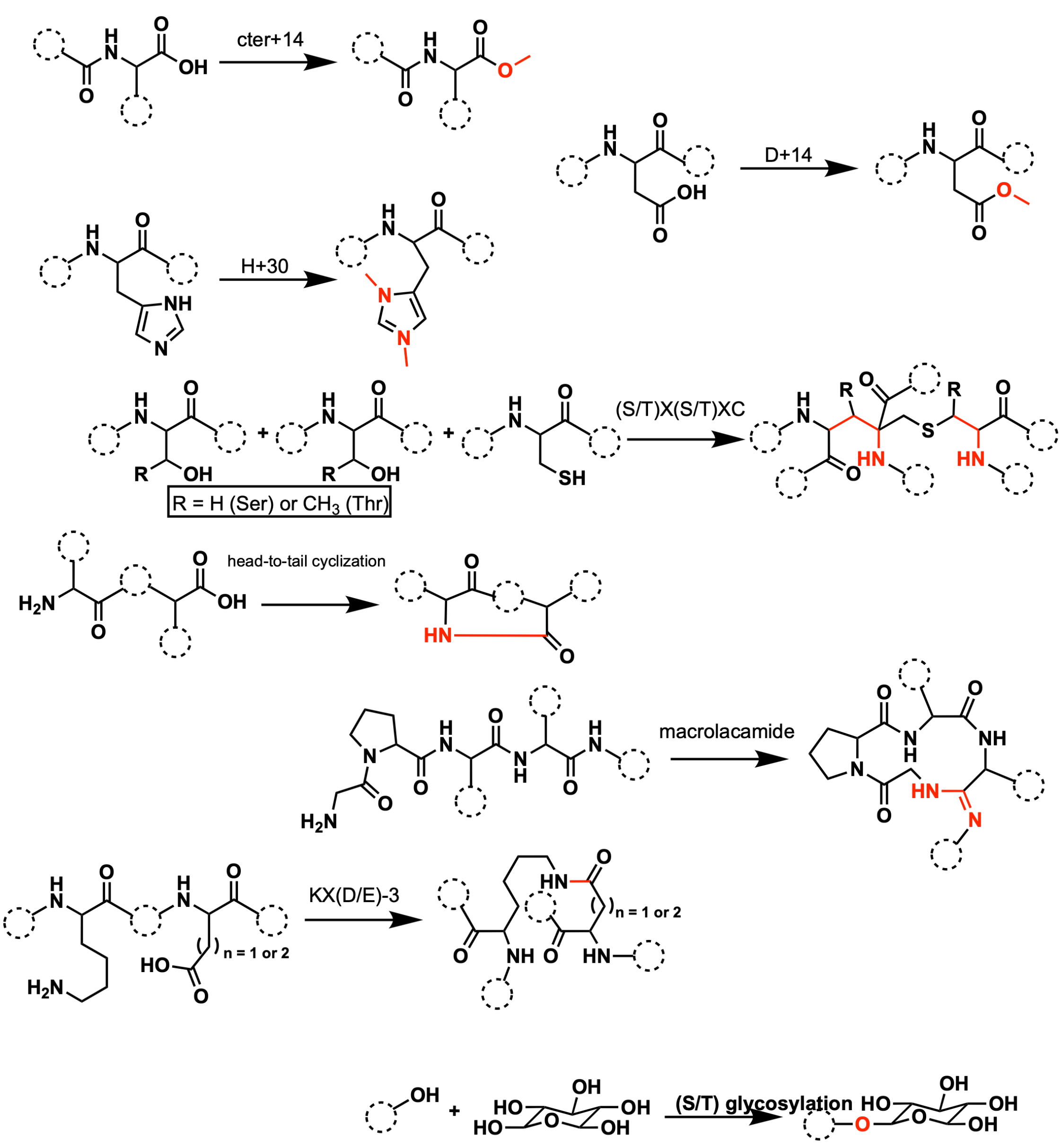
cter+14
D+14
H+30
(S/T)X(S/T)XC
R = H (Ser) or CH3 (Thr)
head-to-tail cyclization
macrolacamide
KX(D/E)-3
n = 1 or 2
(S/T) glycosylation

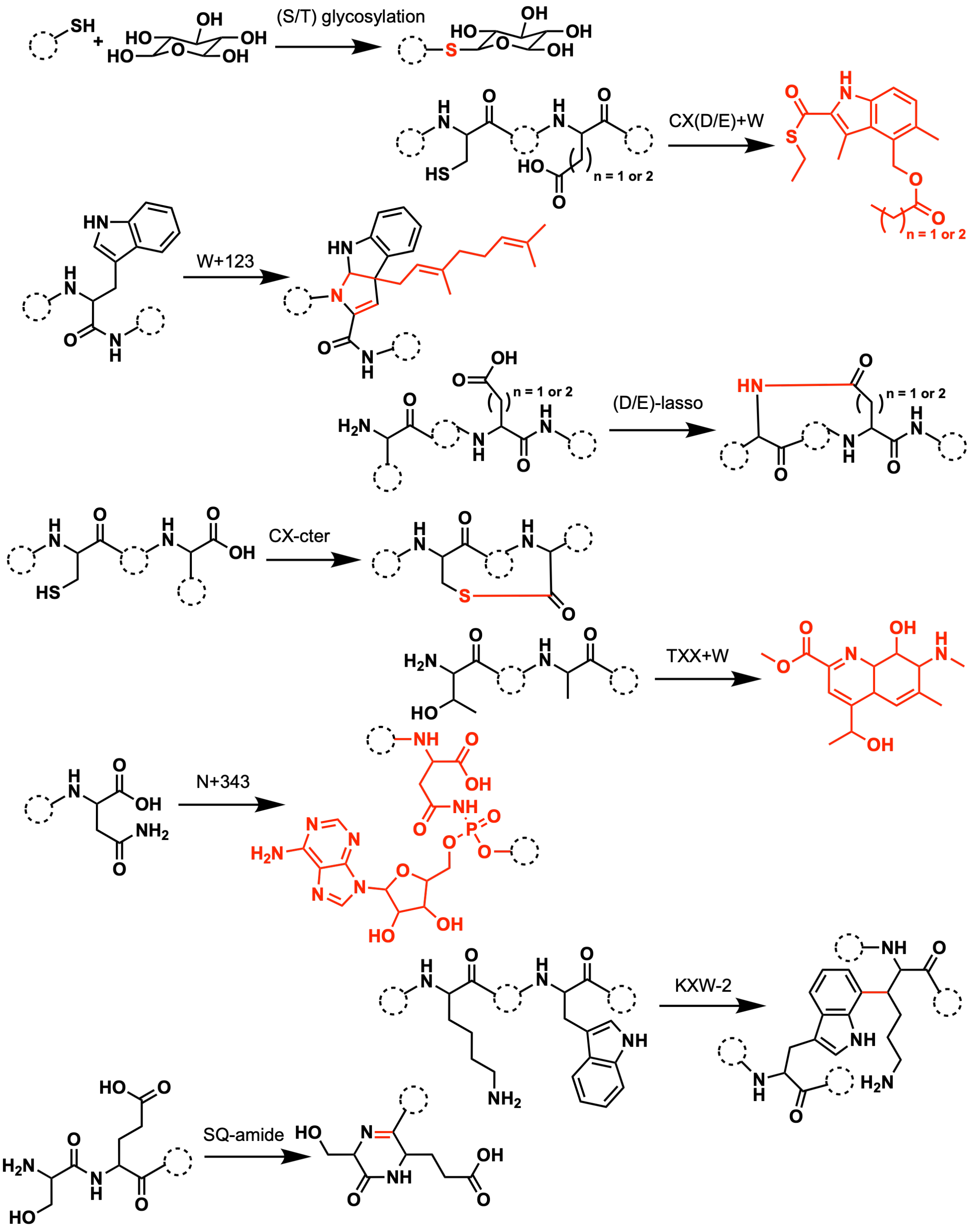
(S/T) glycosylation
CX(D/E)+W
n = 1 or 2
W+123
(D/E)-lasso
CX-cter
TXX+W
N+343
KXW-2
SQ-amide

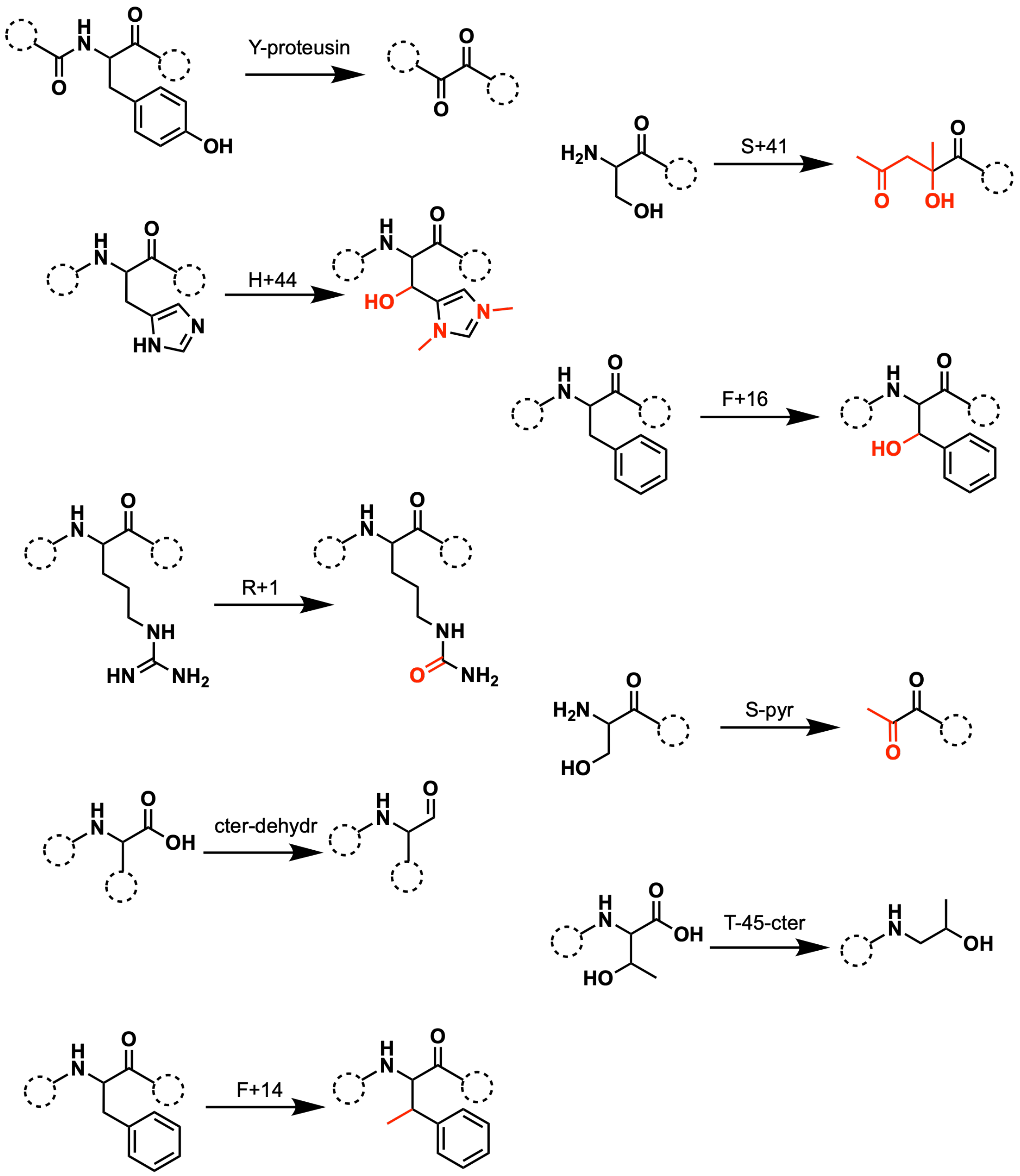
Y-proteusin
S+41
H+44
F+16
R+1
S-pyr
cter-dehydr
T-45-cter
F+14

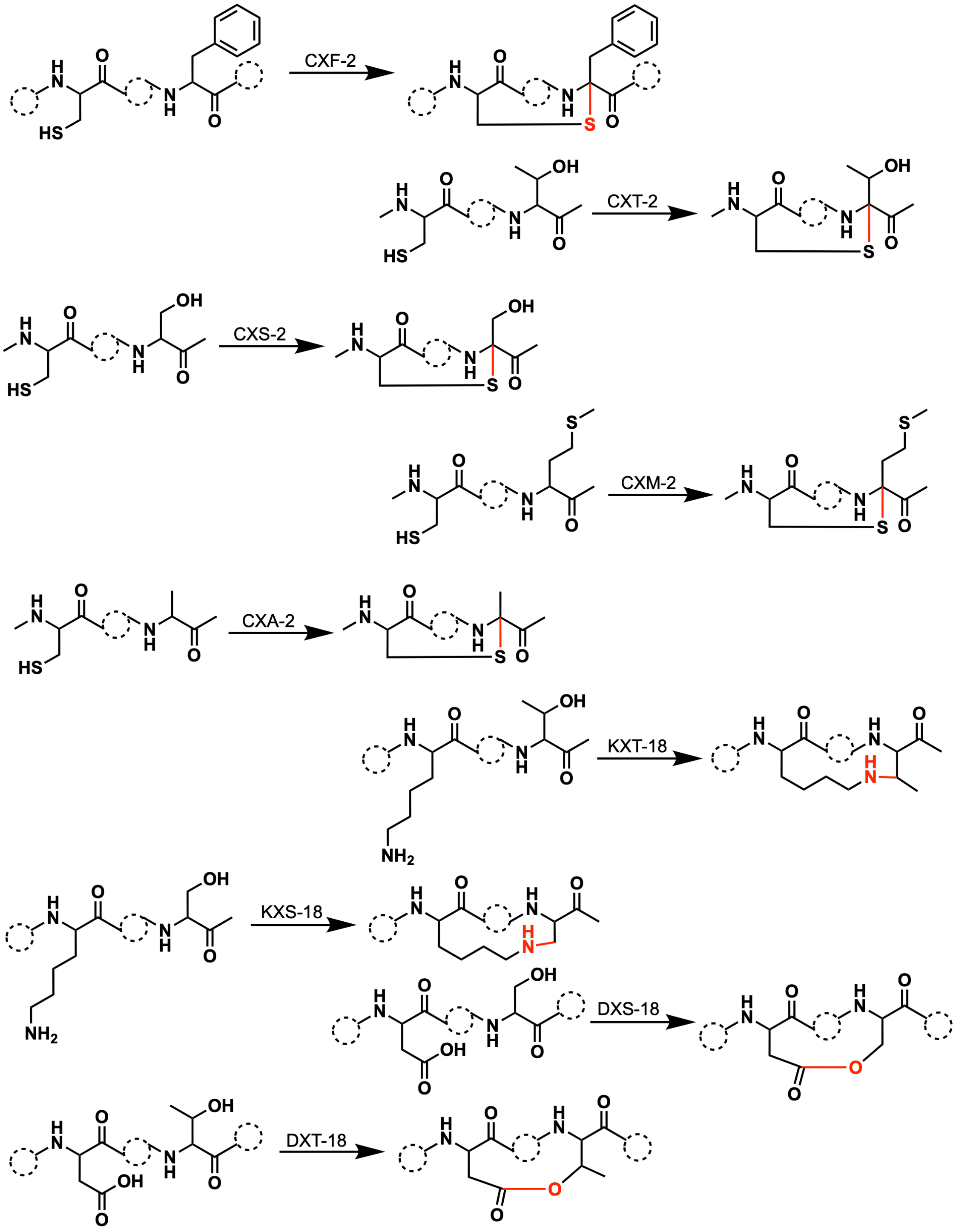
CXF-2
CXT-2
CXS-2
CXM-2
CXA-2
KXT-18
KXS-18
DXS-18
DXT-18

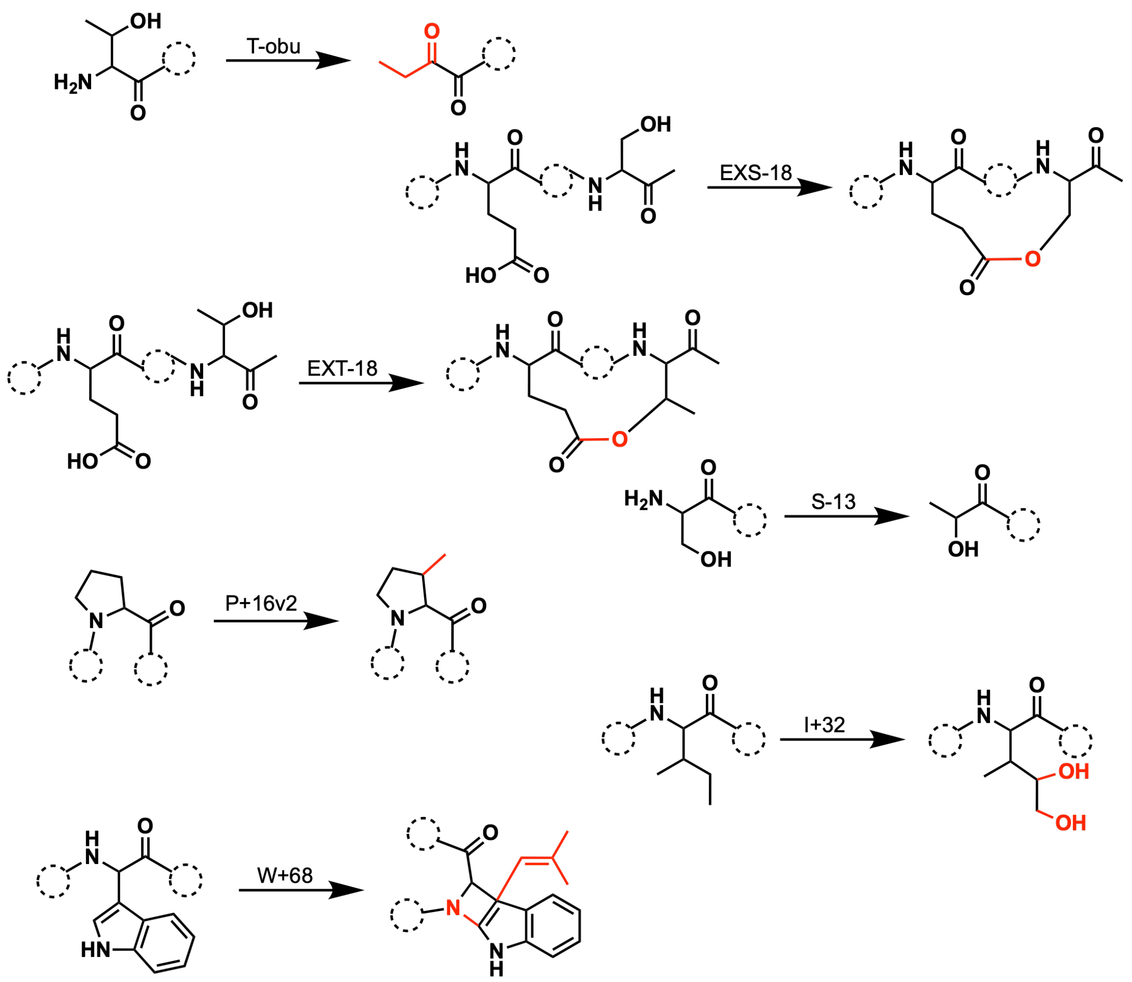

Supplementary Figure 24: The list of RiPP modifications used in this study, compiled from Arnison *et al.* (2013)˜, Montalbán-López *et al.* (2021), and our in-house database. The modification sites are highlighted in red. In each case, the enzyme responsible for the modification is also shown. Table 2 shows the details of these modifications.

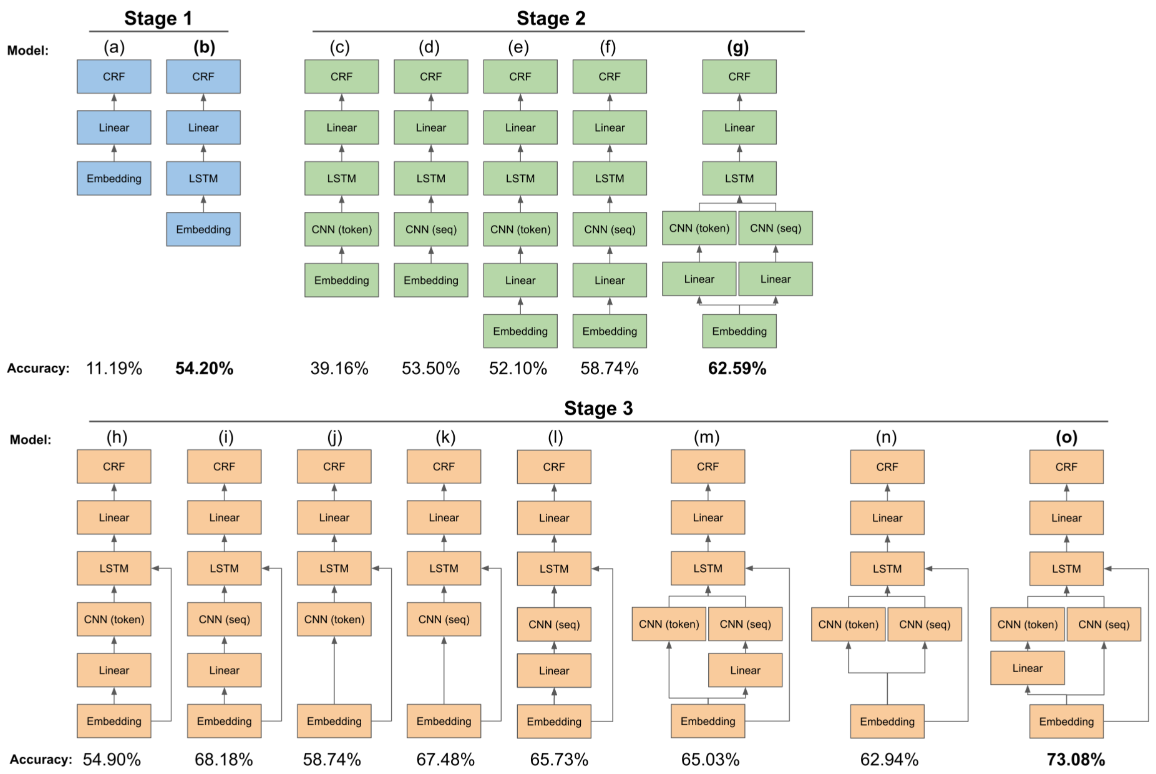

Supplementary Figure 25: Orf2core architecture design. Stage 1 shows improvement of the model accuracy with a simple long short-term memory (LSTM) model to incorporate sequential data, while stage 2 and 3 illustrate addition of a CNN layer to incorporate the information from surrounding amino acids, and addition of dropout and ReLU to stabilize the method. All accuracy scores are calculated with a set of validation data that the model has not accessed during the training process. The best performing model is selected from each stage and carried on to the next stage. In stage 1, a baseline model is built to capture sequential information from the ORF. The starting model (a) contains three essential parts, an embedding layer, which converts a string of amino acids to a matrix of numerical values, a linear layer, which transforms the dimension of the embedded matrix into the dimension that is consistent with conditional random field (CRF), and a CRF layer which predicts the label of each amino acid as core versus non-core (embedding size of 100). In model (b) a bi-directional LSTM layer is incorporated, which learns the sequential information of the ORF sequence (accuracy 54.20%). In stage 2, various ways for incorporating a convolutional neural network (CNN) are experimented to capture patterns of cleavage sites and class-specific patterns (accuracy 62.59%, stride of 1, padding of 2, and kernel size of 5). In stage 3, skip connection is implemented to facilitate information flow [9]. This method was shown to be very accurate in the context of image recognition. A skip connection connects the matrix before CNN layer to the matrix after CNN layer. Then the concatenated matrix is fed into the LSTM layer (accuracy 73.08%). Supplementary Table 7 explores various parameters for embedding size, stride and kernel size, and skip connection settings.

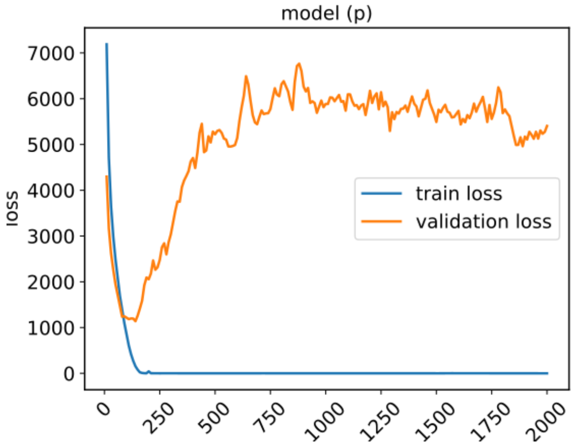

(a)

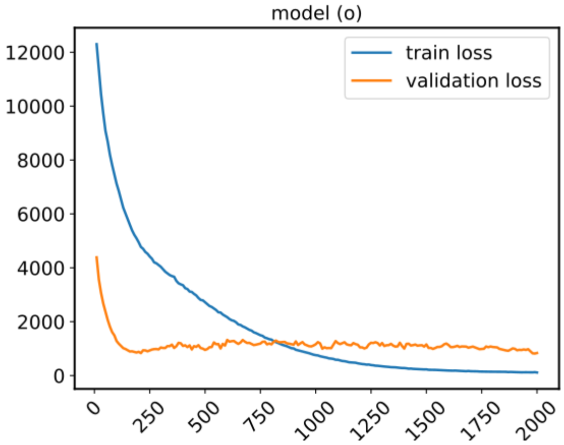

(b)

Supplementary Figure 26: Training and validation loss for orf2core. Overfitting of the model is assessed by plotting the training and validation loss of models (p) and (o). Model (p) suffers from large validation loss, an evidence for overfitting. Addition of regularization in model (o) results in training without validation loss. Models refer to Supplementary Figure 25.

Supplementary Tables

BGC ID	Core	NCBI Taxonomy ID	Locus Accession	Strain Accession	GCA Accession
BGC0000472	TSQIWGSPVP	46234	FJ461733.2	CP003284.1	GCA_000312705.1
BGC0000570	GLSQGVEPDIGQTYFEESRINQD	573065	CP002395.1	CP002395.1	GCA_000175215.2
BGC0000502	TTPLCVGVIIGLTASIKICK	1396	KJ000001.1	CP000227.1	GCA_000013065.1
BGC0000502	TTPLCVGVIIGITTSIKICK	1396	KJ000001.1	CP000227.1	GCA_000013065.1
BGC0000612	SCTTCVCTCSCCTT	226900	AE016877.1	AE016877.1	GCA_000007825.1
BGC0000517	CAWYNISCRLGNKGAYCTLTVECMPSCN	272558	BA000004.3	BA000004.3	GCA_000011145.1
BGC0000517	TTWPCATVGVSVALCPTTKCTSQC	272558	BA000004.3	BA000004.3	GCA_000011145.1
BGC0001173	RCTCTTIISSSSTF	536229	NZ_ABRX01000001.1	CP085037.1	GCA_020535425.1
BGC0000560	TWATIGKTIVQSVKKCRTFTCGCSLGSCSNCN	1423	JX912247.1	CP017072.1	GCA_001719225.1
BGC0000616	LASTLGISTAAAKKAIDIIDAASTIASIISLIGIVTGAGAISYAIVATAKTMIKKYGKKYAAAW	326423	CP000560.1	CP000560.1	GCA_000015785.2
BGC0000569	RCTCTTIISSSSTF	326423	FN668567.1	CP054714.1	GCA_013348705.1
BGC0000572	GTPGFQTPDARVISRFGFN	1249662	CP004117.1	CP004117.1	GCA_000567945.1
BGC0000501	GHGGGGDSGLSVTGCNGHSGISLLCDL	479433	CP001700.1	CP001700.1	GCA_000024025.1
BGC0000574	GAFVGQPEAVNPLGREIQG	509190	CP002008.1	CP002008.1	GCA_000092285.1
BGC0000574	GTLTPGLPEDFLPGHYMPG	509190	CP002008.1	CP002008.1	GCA_000092285.1
BGC0000574	GALVGLLLEDITVARYDPM	509190	CP002008.1	CP002008.1	GCA_000092285.1
BGC0001174	RCSCTTIPCCCCCGG	1267754	CP004085.1	CP004085.1	GCA_000338095.1
BGC0001291	LTANLGISSYAAKKVIDIINTGSAVATIIALVTAVVGGGLITAGIVATAKSLIKKYGAKYAAAW	1352	LC068607.1	CP023074.1	GCA_002290025.1
BGC0000618	TTHSGKYYGN	333849	CP003583.1	CP003583.1	GCA_000174395.2
BGC0000581	GGAGHVPEYFVGIGTPISFYG	562	AF061787.1	CP047577.1	GCA_009905135.1
BGC0000568	VGIGGGGGGGGGGSCGGQGGGCGGCSNGCSGGNGGSGGSGSHI	562	FM877811.1	CP048856.1	GCA_014131515.1
BGC0000516	STIVCVSLRICNWSLRFCPSFKVRCPM	33940	ABVH01000020.1	CP017690.1	GCA_002706565.1
BGC0000515	VTSKSLCTPGCITGVLMCLTQNSCVSCNSCIRC	420246	CP000557.1	CP000557.1	GCA_000015745.1
BGC0001638	SQSPGNCASCSNSASANCTGGLG	574	LN681175.1	LN681175.1	GCA_000826585.2
BGC0001640	STNCFCYPCCSCSAPSSS	1275	MF620092.1	LR134391.1	GCA_900637715.1
BGC0000490	LVATGMAAGVAKTIVNAVSAGMDIATALSLFSGAFTAAGGIMALIKKYAQKKLWKQLIAA	1231377	AMQS01000001.1	AMQS01000001.1	GCA_000300795.2
BGC0000521	KGGSGVIHTISHECNMNSWQFVFTCCS	1360	U91581.1	CP053672.2	GCA_013395015.3
BGC0000607	SCTTCVCTCSCCTT	69966	KM613043.1	CP080014.1	GCA_019378895.1
BGC0000592	FGTTLKYPSDWEEY	449468	AM943877.1	CP046058.1	GCA_010196425.1
BGC0000473	HCATIC	449468	AM774406.1	CP046058.1	GCA_010196425.1
BGC0000474	HCATIC	267872	AM931579.1	CP020771.1	GCA_002095975.1
BGC0001655	GRPNWGFENDWSCVRVC	1245473	NZ_ANAC01000010.1	NZ_ANAC01000010.1	GCA_000341225.1
BGC0000615	SCTTTGCACSSSSSST	756929	HM467197.1	CP003788.1	GCA_000294515.1
BGC0000480	ATGCMC	503013	EU290741.1	CP040094.1	GCA_013343235.1
BGC0000480	ATACAC	503013	EU290741.1	CP040094.1	GCA_013343235.1
BGC0001786	GPPGDRIEFGVLAQLPG	48936	JRVC01000028.1	JRVC01000028.1	GCA_000807925.1
BGC0000471	FIC	179408	CP003614.1	CP003614.1	GCA_000317475.1
BGC0000541	VLSIVACSSGCGSGKTAASCVETCGNRCFTNVGSLC	1406	JX679672.1	CP097771.1	GCA_023586645.1
BGC0000540	ASIIKTTIKVSKAVCKTLTCICTGSCSNCK	1156938	JQ728481.1	CP097771.1	GCA_023586645.1
BGC0000542	VLSIVACSSGCGSGKTAASCVATCGNKCFTNVGSLC	159743	KF111343.1	CP013203.1	GCA_001447315.1
BGC0000571	GGAGQYKEVEAGRWSDR	882378	FR687359.1	FR687359.1	GCA_000198775.1
BGC0001781	GLPIGWWIERPSGWYFPI	161355	BDCX01000012.1	BDCX01000012.1	GCA_001653075.1
BGC0000504	TTPACFTIGLGVGALFSAKFC	2520	L37110.1	CP091905.1	GCA_022213005.1
BGC0000504	TTPVCAVAATAAASSAACGWVGGGIFTGVTVVVSLKHC	2520	L37110.1	CP091905.1	GCA_022213005.1
BGC0000575	GSQLVYREWVGHSNVIKP	132919	AB593691.1	CP038030.2	GCA_006351985.2
BGC0000575	GSQLVYREWVGHSNVIKGP	132919	AB593691.1	CP038030.2	GCA_006351985.2
BGC0000576	GAPSLINSEDNPAFPQRV	983917	AP012320.1	AP012320.1	GCA_000284255.1

Continued on next page

BGC ID	Core	NCBI Taxonomy ID	Locus Accession	Strain Accession	GCA Accession
BGC0000577	GMGSGSTDQNGQPKNLIGGISDD	452662	AP010805.1	AP010805.1	GCA_000091125.1
BGC0000507	SLGPAIKATRQVCPKATRFVTVSCKKSDCQ	1282	Y14023.1	CP097514.1	GCA_023547105.1
BGC0000539	KKKSGVIPTVSHDCHMNSFQFVFTCCS	1292	AB121757.3	CP051645.1	GCA_012851115.1
BGC0000526	GKNGVFKTISHECHLNTWAFLATCCS	59310	DQ835394.1	HE613569.1	GCA_000283635.1
BGC0000552	IGTTVVNSTFSIVLGNKGYICTVTVECMRNCSK	1309	AB179778.1	CP023477.1	GCA_002995555.1
BGC0000531	FSSLSLCSLGCTGVKNPSFNSYCC	1309	AF238860.1	CP044495.1	GCA_008831365.1
BGC0000552	GSTPACAIGVVGITVAVTGISTACTSRCINK	1309	AB179778.1	CP023477.1	GCA_002995555.1
BGC0000557	GKNGVFKTISHECHLNTWAFLATCCS	1314	AF026542.1	LS483338.1	GCA_900475035.1
BGC0000556	VGSRYLCTPGSCWKLVCFTTTVK	1314	AB030831.1	LR590483.1	GCA_901482645.1
BGC0000548	KRGSGWIATITDDCPNSVFVCC	1304	AY005472.1	CP018188.1	GCA_009738225.1
BGC0000547	GNGVVLTLTHECNLATWTKKLKCC	1304	GQ857551.1	FR873481.1	GCA_000253335.1
BGC0000549	FTSHSLCTPGCITGVLMGCHIQSIGCNVHIHISK	1304	JN564797.1	LR134274.1	GCA_900636435.1
BGC0001209	AKGDGWKVM	322159	CP000419.1	CP000419.1	GCA_000014485.1
BGC0000494	LAGYTGIASGTAKKVVDAIDKGAAAFVIISIISTVISAGALGAVSASADFIILTVKNYISRNLKAQAVIW	1349	DQ650653.1	LS483397.1	GCA_900475595.1
BGC0001176	SLGSSPYNDILGYPALIVIYP	183763	JROO01000009.1	JROO01000009.1	GCA_000826685.1
BGC0000546	TGSQISLLICEYSSLSVTLCTP	1886	NZ_DS999645.1	NZ_DS999645.1	GCA_000156475.1
BGC0001548	LLGLAGNDRLVLSKN	68570	LWBU01000091.1	LWBU01000091.1	GCA_001646665.1
BGC0001673	SYHWGDYHDWHHGWYGWWDD	1003195	NC_017586.1	CP003219.1	GCA_000240165.1
BGC0000551	TGSRASLLLCGDSSLSITTCN	100226	AL645882.2	AL645882.2	GCA_000203835.1
BGC0000578	GYFVGSYKEYWSRRII	457431	NZ_DS999644.1	NZ_DS999644.1	GCA_000156455.1
BGC0001753	SCVGTACACSSTSSSS	66858	NZ_JNZK01000003.1	NZ_JNZK01000003.1	GCA_000718455.1
BGC0000496	GSQVSLLVCEYSSLSVVLCTP	455632	AP009493.1	AP009493.1	GCA_000010605.1
BGC0000583	ATPAVAQFVIQGSTICLVC	455632	NC_010572.1	AP009493.1	GCA_000010605.1
BGC0001779	CLGVGSCNDFAGCGYAIVCFW	40318	CP009313.1	CP009313.1	GCA_000819545.1
BGC0001157	GPVVVFDC	680198	FN554889.1	FN554889.1	GCA_000091305.1
BGC0000605	STNCFCYICCSCSS	1073998	JN052143.1	CP041650.2	GCA_007113405.2
BGC0000469	GPVVVFDC	1109705	JX235926.1	CP071123.1	GCA_017163995.1
BGC0001188	ITPLATLATPEATPVGFAATSATAAAVNMITHDVTRH	1553907	NZ_KN708638.1	NZ_KN708638.1	GCA_000805335.1
BGC0001645	LAGQGSPDLLGGHSLL	1463881	NZ_KL591029.1	NZ_KL591029.1	GCA_000716335.1
BGC0001506	GFIGWGNDIFGHYSGGF	1463884	NZ_JOAW01000056.1	NZ_JOAW01000056.1	GCA_000720505.1
BGC0001506	GFIGWGNDIFGHYSGG	1463884	NZ_JOAW01000056.1	NZ_JOAW01000056.1	GCA_000720505.1
BGC0000579	CVWGGDCTDFLGCGTAWICV	463191	NZ_CM000951.1	NZ_CM000951.1	GCA_000154965.1
BGC0000563	ATCECVGLLTLLNTVCIGISCA	953739	HQ328852.1	CP029197.1	GCA_008639165.1
BGC0000518	TDGGGASTVSLLSCISAASVLLCL	591159	GG657757.1	GG657757.1	GCA_000158955.1
BGC0000580	GGPLAGEEMGGITT	925777	AEQX01000392.1	AEQX01000392.1	GCA_000192065.2
BGC0000580	GGPLAGEEIGGFNVPG	925777	AEQX01000392.1	AEQX01000392.1	GCA_000192065.2
BGC0000545	GNGVLKTISHECNMNTWQFLFTCC	935582	AJ276653.2	CP084014.1	GCA_020181455.1

Supplementary Table 1: RiPP BGCs used in tool comparison. For each tested RiPP we include its MIBiG identifier and core sequence. Additionally shown are NCBI accessions for the organism, locus, strain, and genome assembly for each RiPP.

Species	LCMS dataset(s) [.mzML]	Transcriptome dataset(s) [.fasta]	SRA accession
Actaea racemosa	Actaea-racemosa-flower-nBuOH	Actaea_racemosa_rnaSPADES	ERR2040183
Actaea racemosa	Actaea-racemosa-leaf-nBuOH	Actaea_racemosa_rnaSPADES	ERR2040183
Actaea racemosa	Actaea-racemosa-stem-nBuOH	Actaea_racemosa_rnaSPADES	ERR2040183
Allium sativum	Allium-sativum-flower-nBuOH	Allium_sativum_SRR5889575_rnaSPAdes	SRR5889575
Allium sativum	Allium-sativum-leaf-nBuOH	Allium_sativum_SRR5889575_rnaSPAdes	SRR5889575
Allium sativum	Allium-sativum-stem-nBuOH	Allium_sativum_SRR5889575_rnaSPAdes	SRR5889575
Amaranthus hypochondriacus	Amaranthus-hypochondriacus-seed	Amaranthus_hypochondriacus_root_SRR1598913_rnaSPADES	SRR1598913
Amorpha fruticosa	Amorpha-fruticosa-Herbarium-nBuOH	Amorpha_fruticosa_SRR3452781_rnaSPADES	SRR3452781
Ananas comosus	Ananas-comosus-leaf-nBuOH	Ananas_comosus_ERR3412989_rnaSPAdes	ERR3412989
Ananas comosus	Ananas-comosus-leaf-nBuOH	Ananas_comosus_ERR3412992_rnaSPAdes	ERR3412992
Ananas comosus	Ananas-comosus-leaf-nBuOH	Ananas_comosus_ERR3412995_rnaSPAdes	ERR3412995
Anthurium andraeanum	Anthurium-andraeanum-flower-nBuOH	Anthurium_andraeanum_SRR7250906_rnaSPAdes	SRR7250906
Anthurium andraeanum	Anthurium-andraeanum-flower-nBuOH	Anthurium_andraeanum_SRR7497551_rnaSPAdes	SRR7497551
Anthurium andraeanum	Anthurium-andraeanum-leaf-nBuOH	Anthurium_andraeanum_SRR7250906_rnaSPAdes	SRR7250906
Anthurium andraeanum	Anthurium-andraeanum-leaf-nBuOH	Anthurium_andraeanum_SRR7497551_rnaSPAdes	SRR7497551
Apios americana	Apios-americana-Herbarium-nBuOH	Apios_americana_rnaSPADES	ERR706837
Arachis hypogaea	Arachis-hypogaea-Herbarium-nBuOH	Arachis_hypogaea_SRR5867389_rnaSPADES	SRR5867389
Arachis hypogaea	Arachis-hypogaea-nut-and-skin	Arachis_hypogaea_SRR5867389_rnaSPADES	SRR5867389
Arachis hypogaea	Arachis-hypogaea-shell	Arachis_hypogaea_SRR5867389_rnaSPADES	SRR5867389
Areca catechu	Areca-catechu-leaf-nBuOH	Areca_catechu_SRR2071967_rnaSPAdes	SRR2071967
Beta vulgaris	Beta-vulgaris-leaf-nBuOH	Beta_vulgaris_subsp_maritima_rnaSPADES	ERR2040223
Beta vulgaris	Beta-vulgaris-leaf-nBuOH	Beta_vulgaris_taproot_SRR4294172_rnaSPADES	SRR4294172
Beta vulgaris	Beta-vulgaris-root	Beta_vulgaris_subsp_maritima_rnaSPADES	ERR2040223
Beta vulgaris	Beta-vulgaris-root	Beta_vulgaris_taproot_SRR4294172_rnaSPADES	SRR4294172
Beta vulgaris	Beta-vulgaris-stem-nBuOH	Beta_vulgaris_subsp_maritima_rnaSPADES	ERR2040223
Beta vulgaris	Beta-vulgaris-stem-nBuOH	Beta_vulgaris_taproot_SRR4294172_rnaSPADES	SRR4294172
Catharanthus roseus	Catharanthus-roseus-flower-nBuOH	Catharanthus roseus_ERR2112587_rnaSPADES	ERR2112587
Catharanthus roseus	Catharanthus-roseus-flower-nBuOH	Catharanthus_roseus_rnaSPADES	ERR2112587
Catharanthus roseus	Catharanthus-roseus-leaf-nBuOH	Catharanthus roseus_ERR2112587_rnaSPADES	ERR2112587
Catharanthus roseus	Catharanthus-roseus-leaf-nBuOH	Catharanthus_roseus_rnaSPADES	ERR2112587
Catharanthus roseus	Catharanthus-roseus-stem-nBuOH	Catharanthus roseus_ERR2112587_rnaSPADES	ERR2112587
Catharanthus roseus	Catharanthus-roseus-stem-nBuOH	Catharanthus_roseus_rnaSPADES	ERR2112587
Celtis occidentalis	Celtis-occidentalis-leaf-nBuOH	Celtis_occidentalis_rnaSPADES	ERR2040412
Celtis occidentalis	Celtis-occidentalis-stem-nBuOH	Celtis_occidentalis_rnaSPADES	ERR2040412
Chamaemelum nobile	Chamaemelum-nobile-flower-nBuOH	Chamaemelum_nobile_SRR4021831_rnaSPAdes	SRR4021831
Chamaemelum nobile	Chamaemelum-nobile-leaf-nBuOH	Chamaemelum_nobile_SRR4021831_rnaSPAdes	SRR4021831
Chamaemelum nobile	Chamaemelum-nobile-stem-nBuOH	Chamaemelum_nobile_SRR4021831_rnaSPAdes	SRR4021831
Chenopodium quinoa	Chenopodium-quinoa-flower	Chenopodium_quinoa_ERR2040214_rnaSPADES_run2	ERR2040214
Clivia miniata	Clivia-miniata-leaf-nBuOH	Clivia_miniata_SRR7503212_rnaSPAdes	SRR7503212
Clusia rosea	Clusia-rosea-leaf-nBuOH	Clusia_rosea_SRR7412628_rnaSPAdes	SRR7412628
Coffea arabica	Coffea-arabica-leaf-nBuOH	Coffea_arabica_SRR1777904_rnaSPAdes	SRR1777904
Coffea arabica	Coffea-arabica-leaf-nBuOH	Coffea_arabica_SRR1812895_rnaSPAdes	SRR1812895
Coffea arabica	Coffea-arabica-leaf-nBuOH	Coffea_arabica_SRR5417840_rnaSPAdes	SRR5417840
Coffea arabica	Coffea-arabica-leaf-nBuOH	Coffea_arabica_x_Coffea canephora_SRR5417823_rnaSPAdes	SRR5417823
Cunonia capensis	Cunonia-capensis-leaf-nBuOH	Cunonia_capensis_rnaSPADES	ERR2040405
Dicksonia antarctica	Dicksonia-antarctica-leaf-nBuOH	Dicksonia_antarctica_SRR6920681_rnaSPAdes	SRR6920681
Elettaria cardamomum	Elettaria-cardamom-leaf-nBuOH	Elettaria_cardamomum_SRR7280099_rnaSPAdes	SRR7280099
Elaeagnus pungens	Elaeagnus_pungens_root	Elaeagnus_pungens_rnaSPADES	ERR2040413
Gardenia jasminoides	Gardenia-jasminoides-flower-nBuOH	Gardenia_jasminoides_SRR3499217_rnaSPAdes	SRR3499217
Gardenia jasminoides	Gardenia-jasminoides-leaf-nBuOH	Gardenia_jasminoides_SRR3499217_rnaSPAdes	SRR3499217
Ginkgo biloba	Ginkgo-biloba-leaf-nBuOH	Ginkgo_biloba_SRR8599677_rnaSPAdes	SRR8599677
Ginkgo biloba	Ginkgo-biloba-stem-nBuOH	Ginkgo_biloba_SRR8599677_rnaSPAdes	SRR8599677
Gleditsia triacanthos	Gleditsia-triacanthos-Herbarium-nBuOH	Gleditsia_triacanthos_rnaSPADES	ERR706811
Glycine max	Glycine-max-bean	Glycine_max_combined_NCBI_annotated	SRR5487710, SRR5487707, SRR5487702, SRR5487701, SRR5487699, SRR5487698, SRR5487694, SRR5487692, SRR5487691
Glycine max	Glycine-max-embryo	Glycine_max_combined_NCBI_annotated	SRR5487710, SRR5487707, SRR5487702, SRR5487701, SRR5487699, SRR5487698, SRR5487694, SRR5487692, SRR5487691
Glycine max	Glycine-max-Herbarium-nBuOH	Glycine_max_combined_NCBI_annotated	SRR5487710, SRR5487707, SRR5487702, SRR5487701, SRR5487699, SRR5487698, SRR5487694, SRR5487692, SRR5487691
Glycine max	Glycine-max-leaf	Glycine_max_combined_NCBI_annotated	SRR5487710, SRR5487707, SRR5487702, SRR5487701, SRR5487699, SRR5487698, SRR5487694, SRR5487692, SRR5487691
Glycine max	Glycine-max-nodule	Glycine_max_combined_NCBI_annotated	SRR5487710, SRR5487707, SRR5487702, SRR5487701, SRR5487699, SRR5487698, SRR5487694, SRR5487692, SRR5487691

Continued on next page

Species	LCMS dataset(s) [.mzML]	Transcriptome dataset(s) [.fasta]	SRA accession
Glycine max	Glycine-max-pod	Glycine_max_combined_NCBI_annotated	SRR5487710, SRR5487707, SRR5487702, SRR5487701, SRR5487699, SRR5487698, SRR5487694, SRR5487692, SRR5487691
Glycine max	Glycine-max-root	Glycine_max_combined_NCBI_annotated	SRR5487710, SRR5487707, SRR5487702, SRR5487701, SRR5487699, SRR5487698, SRR5487694, SRR5487692, SRR5487691
Glycine max	Glycine-max-stem	Glycine_max_combined_NCBI_annotated	SRR5487710, SRR5487707, SRR5487702, SRR5487701, SRR5487699, SRR5487698, SRR5487694, SRR5487692, SRR5487691
Glycyrrhiza glabra	Glycyrrhiza-glabra-leaf-nBuOH	Glycyrrhiza_glabra_rnaSPADES	ERR706841
Glycyrrhiza glabra	Glycyrrhiza-glabra-stem-nBuOH	Glycyrrhiza_glabra_rnaSPADES	ERR706841
Glycyrrhiza lepidota	Glycyrrhiza-lepidota-Herbarium-nBuOH	Glycyrrhiza_lepidota_ERR2040333_rnaSPADES	ERR2040333
Gymnocladus dioicus	Gymnocladus-dioicus-Herbarium-nBuOH	Gymnocladus_dioicus_rnaSPADES	ERR706843
Hypericum perforatum	Hypericum-perforatum-flower-nBuOH	Hypericum_perforatum_rnaSPADES	ERR2040370
Hypericum perforatum	Hypericum-perforatum-leaf-nBuOH	Hypericum_perforatum_rnaSPADES	ERR2040370
Hypericum perforatum	Hypericum-perforatum-stem-nBuOH	Hypericum_perforatum_rnaSPADES	ERR2040370
Jeffersonia diphylla	Jeffersonia-diphylla-leaf-nBuOH	Jeffersonia_diphylla_SRR675160_rnaSPAdes	SRR675160
Jeffersonia diphylla	Jeffersonia-diphylla-stem-nBuOH	Jeffersonia_diphylla_SRR675160_rnaSPAdes	SRR675160
Lathyrus sativus	Lathyrus-sativus-Herbarium-nBuOH	Lathyrus_sativus_rnaSPADES	ERR706828
Liatris spicata	Liatris-spicata-flower-nBuOH	Liatris_spicata_SRR5237272_rnaSPAdes	SRR5237272
Liatris spicata	Liatris-spicata-leaf-nBuOH	Liatris_spicata_SRR5237272_rnaSPAdes	SRR5237272
Liatris spicata	Liatris-spicata-stem-nBuOH	Liatris_spicata_SRR5237272_rnaSPAdes	SRR5237272
Lycium barbarum	Lycium-barbarum-root	Lycium_barbarum_root_rnaSPADES	SRR6896657
Malus domestica	Malus-domestica-leaf-nBuOH	Malus_domestica_SRR9202286_rnaSPAdes	SRR9202286
Malus domestica	Malus-domestica-stem-nBuOH	Malus_domestica_SRR9202286_rnaSPAdes	SRR9202286
Mangifera indica	Mangifera-indica-leaf-nBuOH	Mangifera_indica_SRR892027_rnaSPAdes	SRR892027
Mangifera indica	Mangifera-indica-leaf-nBuOH	Mangifera_indica_SRR8926025_rnaSPAdes	SRR8926025
Manihot esculenta	Manihot-esculenta-leaf-nBuOH	Manihot_esculenta_SRR5725622_rnaSPAdes	SRR5725622
Medicago truncatula	Medicago-truncatula-leaf	Medicago_truncatula_root_SRR5732302_rnaSPADES	SRR5732302
Medicago truncatula	Medicago-truncatula-root	Medicago_truncatula_root_SRR5732302_rnaSPADES	SRR5732302
Medicago truncatula	Medicago-truncatula-seed	Medicago_truncatula_root_SRR5732302_rnaSPADES	SRR5732302
Medicago truncatula	Medicago-truncatula-seedpod	Medicago_truncatula_root_SRR5732302_rnaSPADES	SRR5732302
Medicago truncatula	Medicago-truncatula-stem	Medicago_truncatula_root_SRR5732302_rnaSPADES	SRR5732302
Medinilla magnifica	Medinilla-magnifica-leaf-nBuOH	Medinilla_magnifica_rnaSPADES	ERR2040321
Melissa officinalis	Melissa-officinalis-leaf-nBuOH	Melissa_officinalis_rnaSPADES	ERR2040574
Melissa officinalis	Melissa-officinalis-stem-nBuOH	Melissa_officinalis_rnaSPADES	ERR2040574
Morinda citrifolia	Morinda-citrifolia-flower-nBuOH	Morinda_citrifolia_rnaSPADES	ERR2040547
Morinda citrifolia	Morinda-citrifolia-leaf-nBuOH	Morinda_citrifolia_rnaSPADES	ERR2040547
Neoregelia carolinae	Neoregelia-carolinae-leaf-nBuOH	Neoregelia_carolinae_SRR3233334_rnaSPAdes	SRR3233334
Oenothera biennis	Oenothera-biennis-flower-nBuOH	Oenothera_biennis_ERR706799_rnaSPADES	ERR706799
Oenothera biennis	Oenothera-biennis-flower-nBuOH	Oenothera_biennis_ERR706800_rnaSPADES	ERR706800
Oenothera biennis	Oenothera-biennis-flower-nBuOH	Oenothera_biennis_ERR706806_rnaSPADES	ERR706806
Oenothera biennis	Oenothera-biennis-flower-nBuOH	Oenothera_biennis_ERR706835_rnaSPADES	ERR706835
Oenothera biennis	Oenothera-biennis-leaf-nBuOH	Oenothera_biennis_ERR706799_rnaSPADES	ERR706799
Oenothera biennis	Oenothera-biennis-leaf-nBuOH	Oenothera_biennis_ERR706800_rnaSPADES	ERR706800
Oenothera biennis	Oenothera-biennis-leaf-nBuOH	Oenothera_biennis_ERR706806_rnaSPADES	ERR706806
Oenothera biennis	Oenothera-biennis-leaf-nBuOH	Oenothera_biennis_ERR706835_rnaSPADES	ERR706835
Oenothera biennis	Oenothera-biennis-stem-nBuOH	Oenothera_biennis_ERR706799_rnaSPADES	ERR706799
Oenothera biennis	Oenothera-biennis-stem-nBuOH	Oenothera_biennis_ERR706800_rnaSPADES	ERR706800
Oenothera biennis	Oenothera-biennis-stem-nBuOH	Oenothera_biennis_ERR706806_rnaSPADES	ERR706806
Oenothera biennis	Oenothera-biennis-stem-nBuOH	Oenothera_biennis_ERR706835_rnaSPADES	ERR706835
Olea europaea	Olea-europaea-leaf-nBuOH	Olea_europaea_rnaSPADES	ERR2040581
Osmanthus fragrans	Osmanthus-fragrans-flower-nBuOH	Osmanthus_fragrans_SRR8790652_rnaSPAdes	SRR8790652
Osmanthus fragrans	Osmanthus-fragrans-flower-nBuOH	Osmanthus_fragrans_SRR8790654_rnaSPAdes	SRR8790654
Osmanthus fragrans	Osmanthus-fragrans-flower-nBuOH	Osmanthus_fragrans_SRR8790657_rnaSPAdes	SRR8790657
Osmanthus fragrans	Osmanthus-fragrans-leaf-nBuOH	Osmanthus_fragrans_SRR8790652_rnaSPAdes	SRR8790652
Osmanthus fragrans	Osmanthus-fragrans-leaf-nBuOH	Osmanthus_fragrans_SRR8790654_rnaSPAdes	SRR8790654
Osmanthus fragrans	Osmanthus-fragrans-leaf-nBuOH	Osmanthus_fragrans_SRR8790657_rnaSPAdes	SRR8790657
Persea americana	Persea-americana-leaf-nBuOH	Persea_americana_SRR8926023_rnaSPAdes	SRR8926023
Petasites hybridus	Petasites-hybridus-leaf-nBuOH	Petasites_hybridus_SRR343129_rnaSPAdes	SRR343129
Petasites hybridus	Petasites-hybridus-stem-nBuOH	Petasites_hybridus_SRR343129_rnaSPAdes	SRR343129
Phaseolus vulgaris	Phaseolus-vulgaris-Herbarium-nBuOH	Phaseolus_vulgaris_ERR2168719_rnaSPADES	ERR2168719
Platycodon grandiflorus	Platycodon-grandiflorus-flower-nBuOH	Platycodon_grandiflorus_rnaSPADES	ERR2040692
Platycodon grandiflorus	Platycodon-grandiflorus-leaf-nBuOH	Platycodon_grandiflorus_rnaSPADES	ERR2040692
Platycodon grandiflorus	Platycodon-grandiflorus-stem-nBuOH	Platycodon_grandiflorus_rnaSPADES	ERR2040692
Podophyllum peltatum	Podophyllum-peltatum-leaf-nBuOH	Podophyllum_peltatum_rnaSPADES	ERR364384
Podophyllum peltatum	Podophyllum-peltatum-stem-nBuOH	Podophyllum_peltatum_rnaSPADES	ERR364384
Pycnanthemum tenuifolium	Pycnanthemum-tenuifolium-flower-nBuOH	Pycnanthemum_tenuifolium_rnaSPADES	ERR2040564
Pycnanthemum tenuifolium	Pycnanthemum-tenuifolium-leaf-nBuOH	Pycnanthemum_tenuifolium_rnaSPADES	ERR2040564
Pycnanthemum tenuifolium	Pycnanthemum-tenuifolium-stem-nBuOH	Pycnanthemum_tenuifolium_rnaSPADES	ERR2040564
Ricinus communis	Ricinus-communis-flower-nBuOH	Ricinus_communis_rnaSPADES	ERR2040364
Ricinus communis	Ricinus-communis-leaf-nBuOH	Ricinus_communis_rnaSPADES	ERR2040364
Ricinus communis	Ricinus-communis-stem-nBuOH	Ricinus_communis_rnaSPADES	ERR2040364
Selaginella kraussiana	Selaginella-kraussiana-aerial	Selaginella_kraussiana_SPAdes_3_14	ERR2040879
Senna hebecarpa	Senna-hebecarpa-Herbarium-nBuOH	Senna_hebecarpa_rnaSPADES	ERR706829
Silybum marianum	Silybum-marianum-flower-nBuOH	Silybum_marianum_rnaSPADES	ERR2040658
Silybum marianum	Silybum-marianum-leaf-nBuOH	Silybum_marianum_rnaSPADES	ERR2040658
Silybum marianum	Silybum-marianum-leaf-nBuOH	Silybum_marianum_rnaSPADES	ERR2040658
Solanum melongena	Solanum-melongena-fruit	Solanum_melongena_SRR1104129_rnaSPADES_55	SRR1104129
Solanum melongena	Solanum-melongena-leaf	Solanum_melongena_SRR1104129_rnaSPADES_55	SRR1104129

Continued on next page

Species	LCMS dataset(s) [.mzML]	Transcriptome dataset(s) [.fasta]	SRA accession
Solanum melongena	Solanum-melongena-root	Solanum_melongena_SRR1104129_rnaSPADES_55	SRR1104129
Solanum melongena	Solanum-melongena-stem	Solanum_melongena_SRR1104129_rnaSPADES_55	SRR1104129
Solanum melongena	Solanum-melongena-seed	Solanum_melongena_SRR1104129_rnaSPADES_55	SRR1104129
Solanum melongena	Solanum-melongena-sprout	Solanum_melongena_SRR1104129_rnaSPADES_55	SRR1104129
Solanum tuberosum	Solanum-tuberosum-sprout	Solanum_tuberosum_combined_annotated	ERR029924, ERR029923, ERR029922, ERR029921, ERR029920, ERR029919, ERR029918, ERR029917, ERR029916, ERR029915, ERR029914, ERR029913, ERR029912, ERR029911, ERR029910, ERR029909
Tanacetum parthenium	Tanacetum-parthenium-flower-nBuOH	Tanacetum_parthenium_rnaSPADES	ERR364394
Tanacetum parthenium	Tanacetum-parthenium-leaf-nBuOH	Tanacetum_parthenium_rnaSPADES	ERR364394
Tanacetum parthenium	Tanacetum-parthenium-stem-nBuOH	Tanacetum_parthenium_rnaSPADES	ERR364394
Tilia americana	Tilia-americana-flower-nBuOH	Tilia_americana_SRR6134087_rnaSPAdes	SRR6134087
Tilia americana	Tilia-americana-leaf-nBuOH	Tilia_americana_SRR6134087_rnaSPAdes	SRR6134087
Tilia americana	Tilia-americana-stem-nBuOH	Tilia_americana_SRR6134087_rnaSPAdes	SRR6134087
Trachycarpus fortunei	Trachycarpus-fortunei-leaf-nBuOH	Trachycarpus_fortunei_SRR6374712_rnaSPAdes	SRR6374712
Vanilla planifolia	Vanilla-planifolia-leaf-nBuOH	Vanilla_planifolia_SRR1509356_rnaSPAdes	SRR1509356
Vanilla planifolia	Vanilla-planifolia-leaf-nBuOH	Vanilla_planifolia_SRR1509370_rnaSPAdes	SRR1509370
Vitex agnus-castus	Vitex-agnus-castus-flower-nBuOH	Vitex_agnus-castus_rnaSPADES	ERR2040567
Vitex agnus-castus	Vitex-agnus-castus-stem-nBuOH	Vitex_agnus-castus_rnaSPADES	ERR2040567
Vitex agnus-castus	Vitex-agnus-castus-leaf-nBuOH	Vitex_agnus-castus_rnaSPADES	ERR2040567
Welwitschia mirabilis	Welwitschia-mirabilis-leaf-nBuOH	Welwitschia_mirabilis_rnaSPADES	ERR364404

Supplementary Table 2: Plant RiPP datasets. This table summarizes the species of plants analyzed using seq2ripp. The table further splits up mass spectra from MassIVE dataset MSV000088918 based on the species. It also includes assembled transcriptome file names and their SRA accession numbers.

Compound name	Sequence	Species	Tissue	Dereplicaotr+ score	PTM Type	PTMs	BURP domain	Reference	SMILES	Peptide Mass	Spectrum Mass	Charge
Legumenin	QPYGVYTW	Arachis hypogaea	stem	31	known	(1), (2)	AhyBURP	Chigumba et al. 2022	CC(C)C1NC(=O)C(NC(=O)C2Cc3ccc(cc3)OC3CC(NC3=O)C(=O)N3CCCC3C(=O)N2)n2cc(c3ccccc32)CC(C(=O)O)NC(=O)C(C(C)O)NC(=O)C(Cc2ccc(O)cc2)NC1=O	991.408	992.415	1
Lyciumin B	QPWGVGSW	Lycium barbarum	root	42	known	(6), (7)	LbaLyca	Kersten, Weng, PNAS (2018)	CC(C)C1NC(=O)C(NC(=O)C(Cc2c[nH]c3ccccc23)NC(=O)C2CCCN2C(=O)C2CCC(=O)N2)n2cc(c3ccccc32)CC(C(=O)O)NC(=O)C(CO)NC(=O)CNC1=O	896.382	897.39	1
Lyciumin D	QPYGVGIW	Lycium barbarum	root	33	known	(6), (7)	LbaLyca	Kersten, Weng, PNAS (2018)	CCC(C)C1NC(=O)C(NC(=O)C(NC(=O)CNC(=O)C(Cc2ccc(O)cc2)NC(=O)C2CCCN2C(=O)C2CCC(=O)N2)C(C)C)n2cc(c3ccccc32)CC(C(=O)O)NC1=O	899.418	900.427	1
[Gln1]-Lyciumin D	QPYGVGIW	Lycium barbarum	root	27	known	(6)	LbaLyca	Kersten, Weng, PNAS (2018)	CCC(C)C1NC(=O)CNC(=O)C(C(C)C)NC(=O)C(NC(=O)C(Cc2ccc(O)cc2)NC(=O)C2CCCN2C(=O)C(N)CCC(N)=O)n2cc(c3ccccc32)CC(C(=O)O)NC1=O	916.444	917.451	1
Lyciumin D	QPYGVGIW	Lycium barbarum	root	25	known	(6), (7)	LbaLyca	Kersten, Weng, PNAS (2018)	CCC(C)C1NC(=O)CNC(=O)C(C(C)C)NC(=O)C(NC(=O)C(Cc2ccc(O)cc2)NC(=O)C2CCCN2C(=O)C2CCC(=O)N2)n2cc(c3ccccc32)CC(C(=O)O)NC1=O	899.418	900.425	1
Lyciumin I	QPYGVYTW	Glycine max	nodule	41	known	(6), (7)	Sali3-2	Kersten, Weng, PNAS (2018)	CC(C)C1NC(=O)C(NC(=O)C(Cc2ccc(O)cc2)NC(=O)C2CCCN2C(=O)C2CCC(=O)N2)n2cc(c3ccccc32)CC(C(=O)O)NC(=O)C(C(C)O)NC(=O)C(Cc2ccc(O)cc2)NC1=O	993.423	994.431	1
Legumenin	QPYGVYTW	Glycine max	nodule	32	known	(2), (6)	Sali3-2	Chigumba et al. 2022	CC(C)C1NC(=O)C(NC(=O)C2Cc3ccc(cc3)OC3CC(NC3=O)C(=O)N3CCCC3C(=O)N2)n2cc(c3ccccc32)CC(C(=O)O)NC(=O)C(C(C)O)NC(=O)C(Cc2ccc(O)cc2)NC1=O	991.408	992.415	1
Selanine A	VLFYPSY	Selaginella kraussiana	aerial	38	known	(5), (9)	SkrBURP	Chigumba et al. 2022	CC(C)C1Oc2ccc(cc2)C2Oc3ccc(cc3)CC(C(=O)O)NC(=O)C(CO)NC(=O)C3CCCN3C(=O)C2NC(=O)C(Cc2ccccc2)NC(=O)C1NC(=O)C(C(C)C)N(C)C	911.443	456.728	2
Cercic acid	QILFW	Cercis canadensis	stem	21	Known	(4), (7)	CcaBURP1	Chigumba et al. 2022	CCC1(C)C(NC(=O)C2CCC(=O)N2)C(=O)NC(CC(C)C)C(=O)NC(Cc2ccccc2)C(=O)NC(C(=O)O)Cc2cn1c1ccccc21	686.343	687.350	1
Stephanotic acid-[LV]	QLLVW	Cercis canadensis	pod	16	Known	(4), (7)	CcaBURP2	Chigumba et al. 2022	CC(C)CC1NC(=O)C(NC(=O)C2CCC(=O)N2)C(C(C)C)c2ccc3c(c[nH]c3c2)CC(C(=O)O)NC(=O)C(C(C)C)NC1=O	638.343	639.350	1
Selanine B	VLFY	Selaginella kraussiana	aerial	6	Known	(8)	SkrBURP	Chigumba et al. 2022	CC(C)C1Oc2ccc(cc2)CC(C(=O)O)NC(=O)C(Cc2ccccc2)NC(=O)C1NC(=O)C(C(C)C)N(C)C	566.310	567.317	1
Elaeagnin	LPIIY	Elaeagnus pungens		15	Novel	(13)	EpuBURP	This Study	CCC(C)C1NC(=O)C(C(C)CC)NC(=O)C2C(CCN2C(=O)C(N)CC(C)C)Oc2ccc(cc2)CC(C(=O)O)NC1=O	615.363	616.371	1
Jdi-947	QPFGVFSW	Jeffersonia diphylla	stem	32	Known	(12)	JdiBURP	This Study	CC(C)C1NC(=O)C(NC(=O)C(Cc2ccccc2)NC(=O)C2CCCN2C(=O)C2CCC(=O)N2)n2cc(c3ccccc32)CC(C(=O)O)NC(=O)C(CO)NC(=O)C(Cc2ccccc2)NC1=O	947.418	948.426	1
Mtr-630	QYGTH	Medicago truncatula	seed	35	Novel	(5), (10)	MtrBRUP_3	This Study	CC(O)C1NC(=O)CNC(=O)C(Cc2ccc(O)cc2)NC(=O)C(N(C)C)C(CC(N)=O)n2cnc(c2)CC(C(=O)O)NC1=O	630.276	631.277	1
Skr-652	GEVTSY	Selaginella kraussiana	aerial	29	Novel	(13)	SkrBURP_2	This Study	CC(C)C1NC(=O)C(NC(=O)CN)C(CC(=O)O)Oc2ccc(cc2)CC(C(=O)O)NC(=O)C(CO)NC(=O)C(C(C)O)NC1=O	652.27	653.281	1

Compound name	Sequence	Species	Tissue	Dereplicaotr+ score	PTM Type	PTMs	BURP domain	Reference	SMILES	Peptide Mass	Spectrum Mass	Charge
Skr-618	TFYY	Selaginella kraussiana	aerial	26	Known + Novel	(5), (14)	SkrBURP_5	This Study	CN(C)C1C(=O)NC(Cc2ccccc2)C(=O)NC(Cc2ccc(O)cc2)C(=O)NC(C(=O)O)Cc2ccc(cc2)OCC1O	618.269	619.276	1
Jdi-931	QPFGVFAW	Jeffersonia diphylla	stem	16	Known	(12)	JdiBURP	This Study	CC1NC(=O)C(Cc2ccccc2)NC(=O)C(C(C)C)NC(=O)C(NC(=O)C(Cc2ccccc2)NC(=O)C2CCCN2C(=O)C2CCC(=O)N2)n2cc(c3ccccc32)CC(C(=O)O)NC1=O	931.423	932.431	1
Sali-635	VPIFY	Glycine max	nodule	25	Novel	(14)	GLYMA_04G180400	This Study	CCC(C)C1NC(=O)C2CC(CN2C(=O)C(N)C(C)C)Oc2ccc(cc2)CC(C(=O)O)NC(=O)C(Cc2ccccc2)NC1=O	635.332	636.339	1
Cca-653	QLKVW	Cercis canadensis	pod	17	Known	(4), (7)	CcaBURP2	This Study	CC(C)C1NC(=O)C(CCCCN)NC(=O)C(NC(=O)C2CCC(=O)N2)C(C(C)C)c2ccc3c(c[nH]c3c2)CC(C(=O)O)NC1=O	653.354	654.361	1
Sali-746	QVPIFY	Glycine max	nodule	20	Known + Novel	(7), (13)	GLYMA_04G180400	This Study	CCC(C)C1NC(=O)C2C(CCN2C(=O)C(NC(=O)C2CCC(=O)N2)C(C)C)Oc2ccc(cc2)CC(C(=O)O)NC(=O)C(Cc2ccccc2)NC1=O	746.364	747.371	1
Skr-552	FLLY	Selaginella kraussiana	aerial	14	Known	(14)	SkrBURP_4	This Study	CC(C)CC1NC(=O)C(NC(=O)C(N)Cc2ccccc2)CC(C)(C)Oc2ccc(cc2)CC(C(=O)O)NC1=O	552.295	553.301	1
Skr-838	FLLYPY	Selaginella kraussiana	aerial	10	known	(5), (9)	SkrBURP	This Study	CC(C)CC1NC(=O)C(NC(=O)C(Cc2ccccc2)N(C)C)C(C(C)C)Oc2ccc(cc2)C2Oc3ccc(cc3)CC(C(=O)O)NC(=O)C3CCCN3C(=O)C2NC1=O	838.427	839.432	1
Skr-546	ILLY	Selaginella kraussiana	aerial	9	known	(5), (8)	SkrBURP	This Study	CCC(C)C(C(=O)NC1C(=O)NC(CC(C)C)C(=O)NC(C(=O)O)Cc2ccc(cc2)OC1C(C)C) N(C)C	546.342	547.348	1
Skr-863	ILLYPSY	Selaginella kraussiana	aerial	31	Known	(5), (9)	SkrBURP	This Study	CCC(C)C(C(=O)NC1C(=O)NC(CC(C)C)C(=O)NC2C(=O)N3CCCC3C(=O)NC(CO)C(=O)NC(C(=O)O)Cc3ccc(cc3)OC2c2ccc(cc2)OC1C(C)C) N(C)C	863.443	432.729	2

Supplementary Table 3: Novel plant RiPPs.

Residue	C	δ(^{13}C) [ppm] [a]	H	δ(^{1}H) (int, m, *J*) [ppm] [b]	COSY [c]	TOCSY [c]	HMBC [d]	ROESY [e]
Leu^1	α	51.2	α	4.34 (1H, m, 5.1, 8.1)	Leu^1: Hβ, Hγ	Leu^1: Hβ, Hγ	n/d	Leu^1: Hβ1, Hδ, Pro^2: Hδ
	β	41.3	β1	1.63 (1H, m)	Leu^1: Hα, Hβ2, Hγ	Leu^1: Hα, Hβ2, Hγ, Hδ	Leu^1: Cδ	Leu^1: Hα, Hδ
			β2	1.66 (1H, m)	Leu^1: Hα, Hβ1, Hγ	Leu^1: Hα, Hβ1, Hγ, Hδ	Leu^1: Cδ	Leu^1: Hα, Hδ, Pro^2: Hδ2
	γ	25.3	γ	1.72 (1H, m)	Leu^1: Hβ1, Hγ, Hδ	Leu^1: Hα, Hβ, Hδ	n/d	Leu^1: Hα, Hβ, Hδ
	δ1	22.2	δ1	1.01 (6H, d, 6.5)	Leu^1: Hγ	Leu^1: Hα, Hβ, Hγ	Leu^1: Cβ, Cγ, Cδ2	Leu^1: Hα, Hγ, Pro^2: Hδ2
	δ2	23.5	δ2	1.01 (6H, d, 6.5)	Leu^1: Hγ	Leu^1: Hα, Hβ, Hγ	Leu^1: Cβ, Cγ, Cδ1	Leu^1: Hα, Hγ, Pro^2: Hδ2
	C=O	169.4	NH	n/d	n/d	n/d	n/d	n/d
Pro^2	α	67.7	α	4.25 (1H, d, 4.6)	Pro^2: Hβ	Pro^2: Hβ, Hγ, Hδ	Pro^2: Cβ	Pro^2: Hβ, Hγ, Tyr^5: H3/5
	β	79.2	β	5.34 (1H, m)	Pro^2: Hα, Hγ, Hδ1	Pro^2: Hα, Hγ, Hδ, Tyr5: H2/6, H3/5	n/d	Pro^2: Hα, Hγ, Hδ2, Tyr^5: H3/5
	γ	33.5	γ1	2.28 (1H, m)	Pro^2: Hβ, Hγ2, Hδ	Pro^2: Hα, Hβ, Hγ2, Hδ	Pro^2: Cβ, Cδ	Pro^2: Hβ, Hγ1, Hδ
			γ2	2.55 (1H, m)	Pro^2: Hβ, Hγ1, Hδ	Pro^2: Hα, Hβ, Hγ1, Hδ	n/d	Pro^2: Hβ, Hγ1, Hδ
	δ	47.4	δ1	4.03 (1H, dd, 9)	Pro^2: Hγ1, Hδ2	Pro^2: Hα, Hβ, Hγ1, Hδ2	Leu^1: C=O, Pro^2: Cα, Cβ, Cγ	Leu^1: Hδ, Pro^2: Hγ, Hδ2
	C=O	173.1	δ2	3.61 (1H, dd, 5.5, 10)	Pro^2: Hγ, Hδ1	Pro^2: Hα, Hβ, Hγ1, Hδ1	Pro^2: Cγ	Leu^1: Hδ, Hβ1, Pro^2: Hβ, Hγ, Hδ
Ile^3	α	61.4	α	4.41 (1H, d, 9)	Ile^3: Hβ, NH	Ile^3: Hβ, Hγ1/2, Hδ, NH	Ile^3: Cβ, Cγ1/2, C=O Ile4: Cα	Ile^3: Hβ, Hγ1, Hγ2
	β	39.3	β	1.75 (1H, m)	Ile^3: Hα, Hγ1	Ile^3: Hα, Hγ1/2, Hδ	n/d	Ile^3: Hα, Hγ1, Hγ2A
	γ1	16.1	γ1	0.94 (3H, d, 6.6)	Ile^3: Hβ	Ile^3: Hα, Hγ1/2, Hδ	Ile^3: Cα, Cβ, Cγ2	Ile^3: Hα, Hβ
	γ2	25.6	γ2A	1.19 (1H, m)	Ile^3: Hγ2B, Hδ	Ile^3: Hα, Hβ, Hγ1/2B, Hδ	Ile^3: Cγ1	Ile^3: Hα, Hβ, Hγ2B, Hδ
			γ2B	1.58 (1H, m)	Ile^3: Hγ2A, Hδ	Ile^3: Hα, Hβ, Hγ1/2A, Hδ	n/d	Ile^3: Hδ, Hγ2A
	δ	11.4	δ	0.9 (3H, d, 7.6)	Ile^3: Hβ, Hγ2	Ile^3: Hα, Hβ, Hγ1/2	Ile^3: Cβ, Cγ2	Ile^3: Hα, Hβ, Hγ2A
	C=O	172.5	NH	8.5 (d, 10.3)	Ile^3: Hα	Ile^3: Hα	n/d	n/d
Ile^4	α	58.7	α	4.32 (1H, d, 8.3)	Ile^4: Hβ, NH	Ile^4: Hβ, Hγ1/2, Hδ, NH	Ile^4: Cβ, Cγ1/2, C=O	Ile^4: Hβ, Hγ1/2, Hδ
	β	38.8	β	1.69 (1H, m)	Ile^4: Hα	Ile^4: Hα, Hγ1/2, Hδ	n/d	Ile^4: Hα, Hγ1, Hγ2A/B, Hδ
	γ1	15.6	γ1	0.87 (3H, d, 6.4)	Ile^4: Hβ, Hγ2	Ile^4: Hα, Hβ, Hγ2B	Ile^4: Cα, Cβ, Cγ2	Ile^4: Hα, Hβ, Hγ2A
	γ2	26.4	γ2A	1.14 (1H, m)	Ile^4: Hβ, Hγ1/2B, Hδ	Ile^4: Hα, Hβ, Hγ1, Hγ2B, Hδ	n/d	Ile^4: Hγ2B, Hδ
			γ2B	1.52 (1H, m)	Ile^4: Hβ, Hγ1/2A, Hδ	Ile^4: Hα, Hβ, Hγ1, Hγ2A, Hδ	n/d	Ile^4: Hγ2A, Hδ
	δ	11.3	δ	0.87 (3H, d, 6.4)	Ile^4: Hβ, Hγ2	Ile^4: Hα, Hβ, Hγ1, Hγ2A	Ile^4: Cα, Cβ, Cγ1/2	Ile^4: Hα, Hβ, Hγ2A
	C=O	172.3	NH	7.2 (1H, d, 8.3)	Ile^4: Hα	Ile^4: Hα	n/d	n/d
Tyr^5	α	55.1	α	4.59 (1H, d, 12)	Tyr^5: Hβ	Tyr^5: Hβ	Ile^4: C=O, Tyr^5: Cβ, C1/2/6, C=O	Tyr^5: Hβ2, H2/6
	β	39.5	β1	2.57 (1H, m)	Tyr^5: Hα, Hβ2	Tyr^5: Hα, Hβ2	Tyr^5: Cα, C1	Tyr^5: Hβ2, H2/6
			β2	3.25 (1H, dd, 1.6, 12)	Tyr^5: Hα, Hβ1	Tyr^5: Hα, Hβ1	Tyr^5: C1	Tyr^5: Hβ1, H2/6
	C1	132	H2	7.08 (2H, d, 7.5)	Tyr^5: H3/5	Pro^2: Hβ, Tyr^5: H3/5	Tyr^5: Cβ, C1, C4, C6	Tyr^5: Hα, Hβ, H3/5
	C2	132.1	H3	6.69 (2H, d, 7.5)	Tyr^5: H2/6	Pro^2: Hβ, Tyr^5: H2/6	Tyr^5: C2, C3, C4, C5, C6	Pro^2: Hβ, Tyr^5: H2/6
	C3	115.7	H5	6.69 (2H, d, 7.5)	Tyr^5: H2/6	Pro^2: Hβ, Tyr^5: H2/6	Tyr^5: C2, C3, C4, C5, C6	Pro^2: Hβ, Tyr^5: H2/6
	C4	157.9	H6	7.08 (2H, d, 7.5)	Tyr^5: H3/5	Pro^2: Hβ, Tyr^5: H3/5	Tyr^5: Cβ, C1, C4, C6	Tyr^5: Hα, Hβ, H3/5
	C5	115.7	NH	n/d	n/d	n/d	n/d	n/d
	C6	132.1						
	C=O	174.4						

Supplementary Table 4: NMR data for Elaeagnin (MeOD-d4, 300 K). [a] ^{13}C NMR data of isolated elaeagnin in MeOD-d4 (200 MHz). Chemical shift values were derived from ^{13}C NMR analysis, HSQC analysis and HMBC analysis (Supplementary Figure 18). [b] ^{1}H NMR data of isolated elaeagnin in MeOD-d4 (800 MHz). Multiplicity m (s=singlet, d=doublet, t=triplet, dd=double doublet, m=multiplet, br=broad signal), intensity int, coupling constants J in Hertz. Chemical shift values in ppm were derived from ^{1}H NMR analysis, COSY analysis and TOCSY analysis (Supplementary Figure 18). [c] COSY and TOCSY correlations of isolated elaeagnin in MeOD-d4 (Supplementary Figure 18). [d] HMBC correlations elaeagnin in MeOD-d4 in DMSO-d6 (Supplementary Figure 18). [e] ROESY correlations of elaeagnin in MeOD-d4 (Supplementary Figure 18).

ABC-membrane	ABC-tran	acyG	ADH-N
adh-short	ADH-zinc-N	AED99784-AA	ageF1
ageF2	ageG	Aldedh	Amidohydro-2
Antibiotic-NAT	Antimicr18	Asn-synthase	B12-binding
Bac-luciferase	BacteriocIIc-cy	Bacteriocin-IIc	Bacteriocin-IId
Bacteriocin-II	berD	berI	BRN51-AA
btmB	btmC	btmG	btmK
BURP	CclB-AA	Cinorf7	CinX
ClassIIILanti	ClassIILanti	ClassILanti	ClassIVLanti
crnJ	Cupin-4	Cupin-8	CypL
CypM	divMT-AA	divX-AA	DsbA
DsbB	DUF4135	durN	eciO
elxO	EntA-Immun	Enterotoxin-ST	FAD-binding-1
Flavodoxin-1	Flavoprotein	FMN-red	Fungal-trans-2
Gallidermin	garM	GarO	GATase-7
Glycos-transf-2	Hydrolase-4	JmjC	kgpF-AA
kgpF	kocD	kocH	labKC
lacticin-l	lacticin-mat	LANC-like	Lant-dehydr-C
Lant-dehydr-N	L-biotic-A	leader-abc	leader-d
leader-eh	LMOSLCC2540-AA	LnqD-AA	LtnJ
mature-ab	mature-a	mature-b	mature-d
mature-ha	mbnB-AA	mbnB	mbnC-AA
mbnC	mcbB	mccE-AA	mclI-AA
McmA-AA	mdnB	mdnC	Methyltransf-11
Methyltransf-25	Methyltransf-31	MibHS	MibO
MreB-Mbl	mslE	mslF	MTS
mvdC	mvdD	NAD-binding-1	Nif11
Nitroreductase	NocC-AA	nocC	NocE-AA
NO-synthase	orf3z-AA	p450	paeN
pcpX	Peptidase-C1	Peptidase-C39	Peptidase-C50
PhyH	PqqD-AA	Pro-isomerase	Radical-SAM
rgg	RSAM-AA	sphB-AA	sphC-AA
sphC	strB	strC	T4SS-CagC
tclM	tclP	Thioredoxin-8	ThnB-AA
ThnP-AA	TIGR03731	triH	triK
Trp-halogenase	truF1	truF2	tvaG
U-box	Ufd2P-core	UstYa	virF1
virF2	virG	YcaO	Zn-clus

Supplementary Table 5: 152 HMM profiles used to identify RiPP BGCs in genome2bgc.

mod	an example peptide name	peptide sequence	name of enzyme	class
F+68	Aeruginosamide C	FFPVC	PF08241 Methyltrans_11	Cyanobactin
Y+68	Aesturamides7-12	ACMPCYP	PF08241 Methyltrans_11	Cyanobactin
S+68	Trunkamide	TSIAPFC	PatF	Cyanobactin
T+68	Trunkamide	TSIAPFC	PatF	Cyanobactin
C-6_cter	Aeruginosamide C	FFPVC	TsrB	Cyanobactin
C-18	Aerucyclamide A	ITGCIC	LazE (PF02624)	Cyanobactin
T-20	Radamycin	SCVGSACACSSSSSS	McbC/D (PF02624)	Thiopeptide
S-20	plantazolicin A	RCTCTTIISSSSTF	McbC/D (PF02624)	LAP
M+28	venepeptide	MNVITNLLAGVVHFLGWLV	Non-enzymatic	N-formylated
C+162	Sublancin 168	GLGKAQCAALWLQCASGGTIGCGGGAVA CQNYRQFCR	Glycos-transf-2 (PF00535)	glycocin
S-18	Radamycin	SCVGTACACSSTSSSS	LanB/M (PF05147)	Thiopeptide
S-17	pinensin A	SHPTHTVATDDQGHLCTTIICA	SSF51735	Lanthipeptide
T-17	Pep5	TAGPAIRASVKQCQKTLKATRLFTVSCKG KNGCK	SSF51735	Lanthipeptide
S-15	Epilancin 15X	SASIVKTTIKASKKLCRGFTLTCGCHFTG KK	ElxO (SSF51735)	Lanthipeptide
CXC-80	cypemycin	ATPATPTVAQFVIQGSTICLVC	Flavoprotein (PF02441)	Linaridin
SXC-64	epidermin	IASKFICTPGCAKTGSFNSYCC	Flavoprotein (PF02441)	Lanthipeptide
TXC-64	epidermin	IASKFICTPGCAKTGSFNSYCC	Flavoprotein (PF02441)	Lanthipeptide
S-16	Lacticin 3147 A1	CSTNTFSLSDYWGNNGAWCTLTHECMAW CK	LtnJ	Lanthipeptide
D+16	cinnamycin	CRQSCSFGPFTFVCDGNTK	McbC/D (PF02624)	Lanthipeptide
C-1	methanobactin	LCGSCYPCSCM	DsbB (PF02600)	Methanobactin
W+34	microbisporicin A1	VTSWSLCTPGCTSPGGGSNCSFCC	MibH (PF04820)	Lanthipeptide
P+16	phalloidin	AWLATCP	MibO (PF00067)	Phallotoxins
P+32	microbisporicin A1	VTSWSLCTPGCTSPGGGSNCSFCC	MibO (PF00067)	Lanthipeptide
SXC+16	Actagardine	SSGWVCTLTIECGTVICAC	GarO (PF00296)	Lanthipeptide
TXC+16	Actagardine	SSGWVCTLTIECGTVICAC	GarO (PF00296)	Lanthipeptide
A+42	microviridin J	ISTRKYPSDWEEW	mdnD	Microviridin
T-18	Lacticin 3147 A1	CSTNTFSLSDYWGNNGAWCTLTHECMAW CK	LanB/M (PF05147)	Lanthipeptide
T-16	Nisin A	ITSISLCTPGCKTGALMGCNMKTATCHCS IHVSK	FMN-red (PF03358)	Lanthipeptide
C-20	Radamycin	SCVGTACACSSTSSSS	McbC/D (PF02624)	Thiopeptide
R+28	plantazolicin A	RCTCTTIISSSSTF	Methyltransf-31 (PF13847)	LAP
CXD-18	Siamycin I	CLGVGSCNDFAGCGYAIVCFW	McjC	Lasso peptide
CXE-18	Siamycin I	CLGVGSCNDFAGCGYAIVCFW	McjC	Lasso peptide
GXD-18	RES-701-1	GNWHGTAPDWFFNYYW	McjC	Lasso peptide
GXE-18	Lariatin A	GSQLVYREWVGHSNVIKP	McjC	Lasso peptide
A+28	Cypemycin	IASKFICTPGCAKTGSFNSYCC	CypM (PF13649)	Linaridin
I+28	Cypemycin	IASKFICTPGCAKTGSFNSYCC	CypM (PF13649)	Linaridin
L+28	Cypemycin	IASKFICTPGCAKTGSFNSYCC	CypM (PF13649)	Linaridin
C-6_pyrazinedione	methanobactin	LCGSCYPCSCM	mbnB/C/E/H/S	Methanobactin
C-4	methanobactin	LCGSCYPCSCM	mbnB/C/E/H/S	Methanobactin
L-1	methanobactin	LCGSCYPCSCM	unknown enzyme	Methanobactin
T+80	methanobactin	RCASTCAATNG	mbnS	Methanobactin
T+39	Polytheonamides	TGIGVVVAVVAGAVANTGAGVNQVAGG NINVVGNINVNANVSVNMNQTT	PoyF (PF13575)	Proteusin
M+44	Polytheonamides	TGIGVVVAVVAGAVANTGAGVNQVAGG NINVVGNINVNANVSVNMNQTT	B12-binding (PF01497)	Proteusin
I+14	Polytheonamides	TGIGVVVAVVAGAVANTGAGVNQVAGG NINVVGNINVNANVSVNMNQTT	B12-binding (PF01497)	Proteusin
Q+14	Polytheonamides	TGIGVVVAVVAGAVANTGAGVNQVAGG NINVVGNINVNANVSVNMNQTT	B12-binding (PF01497)	Proteusin
V+14	Polytheonamides	TGIGVVVAVVAGAVANTGAGVNQVAGG NINVVGNINVNANVSVNMNQTT	Radical_SAM (PF04055)	Proteusin
T+14	Polytheonamides	TGIGVVVAVVAGAVANTGAGVNQVAGG NINVVGNINVNANVSVNMNQTT	B12-binding (PF01497)	Proteusin
N+14	Polytheonamides	TGIGVVVAVVAGAVANTGAGVNQVAGG NINVVGNINVNANVSVNMNQTT	MTS (PF05175)	Proteusin
N+16	Polytheonamides	TGIGVVVAVVAGAVANTGAGVNQVAGG NINVVGNINVNANVSVNMNQTT	Cupin-4 (PF08007)	Proteusin
N+30	Polytheonamides	TGIGVVVAVVAGAVANTGAGVNQVAGG NINVVGNINVNANVSVNMNQTT	Cupin-4 (PF08007)	Proteusin
V+16	Polytheonamides	TGIGVVVAVVAGAVANTGAGVNQVAGG NINVVGNINVNANVSVNMNQTT	TpdJ1/2 (PF00067)	Proteusin
SXC-Lan/TXC-MeLan	Nisin A	ITSISLCTPGCKTGALMGCNMKTATCH CSIHVSK	LANC_like (PF05147)	Lanthipeptide
SXS_pyridine	Radamycin	SCVGTACACSSTSSSS	LazC (PF14028)	Thiopeptide
N-homolog	Radamycin	SCVGTACACSSTSSSS	kocH	Thiopeptide
Cter-1	Siomycin A	VSSASCTTCICTCSCSS	TsrC (PF00733)	Thiopeptide
S-20-zoline	goadsporin	ATVSTILCSGGTLSSAGCV	YcaO (PF02624)	LAP
T-20-zoline	goadsporin	ATVSTILCSGGTLSSAGCV	YcaO (PF02624)	LAP
C-20-zoline	patellamide A	ITVCISVC	YcaO (PF02624)	Cyanobactin
F-4	YM 216391	FIVGSSSC	Maf (PF02545)	YM-216391 family peptides
C-6_thia	thiomuracin A	SCNCFCYICCSCSSA	Radical_SAM (PF04055)	Thiopeptide
C+24	GE2270	SCNCVCGFCCSCSPSA	Methyltransf 2 (PF00891)	Thiopeptide

mod	example peptide name	peptide sequence	name of enzyme	class
(S/T)X(S/T)XC	catenulipeptin	GHGGGGDSGLSVTGCNGHSGISLLCDL	Pkinase (PF00069)	Lanthipeptide
SXS_pyridine_hydroxy	nocathiacin	SCTTCECSCSCSS	Cytochrome_CBB3 (PF13442)	Thiopeptide
SXS_dehydropiperidine	siomycin A	VSSASCTTCICTCSCSS	Adh_short (PF00106)	Thiopeptide
SXS_piperidine	thiopeptin	VASASCTTCICTCSCSS	NadA (PF02445)	Thiopeptide
macrolacamide	bottromycin A2	GPVVVFDC	Peptidase_M27 (PF01742)	Bottromycin
CX(D/E)+W	nosiheptide	SCTTCECCCSCSS	Abhydrolase6 (PF12697)	Thiopeptide
TXX+W	Thiostrepton	IASASCTTCICTCSCSS	Aminotran_1_2 (PF00155)	Thiopeptide
(S/T)_glycosylation	glycocin F	KPAWCWYTLAMCGAGYDSGTCDYMYSHCFGIKHHSSGSSSYHC	Endonuclease_NS (PF01223)	Sactipeptides
C_glycosylation	Sublancin 168	GLGKAQCAALWLQCASGGTIGCGGGAVACQNYRQFCR	Glycos_transf_2 (PF00535)	glycocin
CXX-2	subtilosin A	NKGCATCSIGAACLVDGPIPDFEIAGATGLFGLWG	Alba (PF01918)	sactipeptide
(D/E)_lasso	capistruin	GTPGFQTPDARVISRFGFN	Asn_synthase (PF00733)	Lassopeptide
AXC-2	cyclothiazomycin	SNCTSTGTPASCCSCCCC	CltM	Thiopeptide
N+343	microcin C7	MRTGNAD	ThiF (PF00899)	Microcin
X+16	thioviridamide	SVMAAAASIALHC	YcaO (PF02624)	Thioamitide
W+123	comX	ADPITRQWGD	polyprenyl_synt (PF00348)	ComX
Cter+14	Aeruginosamide C	FFPVC	T4SS_CagC (PF16943)	Cyanobactin
CXcter	Agr autoinducing peptide	YSTCDFIM	AgrB (PF04647)	Autoinducing peptide
Cter+70	hominicin	ITPATPFTPAITEITAAVIAX	Unknown	Lanthipeptide
T-46	TP-1161	SCTTTGCACSSSSSST	Fe-ADH (PF00465)	Thiopeptide
Nter+68	Aeruginosamide C	FFPVC	DHBP_synthase (PF00926)	Cyanobactin
Nter+42	goadsporin	ATVSTILCSGGTLSSAGCV	Acetyltransf_3 (PF13302)	LAP
H+30	thioviridamide	SVMAAAASIALHC	SAM-dependent_Mtases (IPR029063)	Thioamitide
T+12	nocathiacin	SCTTCECSCSCSS	BPD_transp_1 (PF00528)	Thiopeptide
D-14	bottromycin A2	GPVVVFDC	LCM (PF04072)	Bottromycin
T-14_ether	thiocillin	SCTTCVCTCSCCTT	Methyltransf_11 (PF08241)	Thiopeptide
I+14_ethylene_oxide	thiomuracin A	SCNCFCYICCSCSSA	p450 (PF00067)	Thiopeptide
I+14_pyrrolidinol	GE 37468	STNCXCYICCSCSSN	p450 (PF00067)	Thiopeptide
KXW-2	streptide	AKGDGWKVM	Carb_kinase (PF01256)	Streptide
KX(D/E)-3	microviridin B	FGTTLKYPSDWEEY	RimK (PF08443)	Graspetide
head-to-tail cyclization	patellamide A	ITVCISVC	PGM_PMM_I (PF02878)	Cyanobactin
I+32	siomycin A	VSSASCTTCICTCSCSS	p450 (PF00067)	Thiopeptide
6m-nitrogen-heterocycle	siomycin A	VSSASCTTCICTCSCSS	adh_short (PF00106)	Thiopeptide
P+16v2	Bottromycin A2	GPVVVFDC	btmC	Bottromycin
F+14	Bottromycin A2	GPVVVFDC	btmC	Bottromycin
S-13	Epicidin 280	SLGPAIKATRQVCPKATRFVTVSCKKSDCQ	eciO	Lanthipeptide
T-obu	pep5	TAGPAIRASVKQCQKTLKATRLFTVSCKGKNGCK	L_biotic_typeA (PF04604)	Lanthipeptide
S-pry	plantaricin W beta	SGIPCTIGAAVAASIAVCPTTKCSKRCGKRKK	L_biotic_typeA (PF04604)	Lanthipeptide
CXF-2	subtilosin A	NKGCATCSIGAACLVDGPIPDFEIAGATGLFGLWG	PqqD (PF05402)	sactipeptide
CXT-2	subtilosin A	NKGCATCSIGAACLVDGPIPDFEIAGATGLFGLWG	PqqD (PF05402)	sactipeptide
CXS-2	subtilosin A	NKGCATCSIGAACLVDGPIPDFEIAGATGLFGLWG	PqqD (PF05402)	sactipeptide
CXA-2	subtilosin A	NKGCATCSIGAACLVDGPIPDFEIAGATGLFGLWG	PqqD (PF05402)	sactipeptide
CXM-2	subtilosin A	NKGCATCSIGAACLVDGPIPDFEIAGATGLFGLWG	PqqD (PF05402)	sactipeptide
DXS-18	microviridin B	FGTTLKYPSDWEEY	MvdD (IPR026439)	Graspetide
DXT-18	microviridin B	FGTTLKYPSDWEEY	MvdD (IPR026439)	Graspetide
ExS-18	microviridin B	FGTTLKYPSDWEEY	MvdD (IPR026439)	Graspetide
EXT-18	microviridin B	FGTTLKYPSDWEEY	MvdD (IPR026439)	Graspetide
T-45-cter	micrococcin P1	SCTTCVCTCSCCTT	adh_short (PF00106)	Thiopeptide
Cter_dehydr	enterocin A	MAKEFGIPAAVAGTVLNVVEAGGWVTTIVSILTAVGSGGLSLLAAAGRE-SIKAYLKKEIKKKGKRAVIAW	Unknown	Head-to-Tail Cyclized
R+1	citrulassin	LLGLAGNDRLVLSKN	Unknown	Lassopeptide
KXS-18	cinnamycin	CRQSCSFGPFTFVCDGNTK	durN	Lanthipeptide
KXT-18	cinnamycin	CRQSCSFGPFTFVCDGNTK	durN	Lanthipeptide
W+68	kawaguchipeptin A	WLNGDNNWSTP	kgpF	Cyanobactin
SQ-amide	klebsazolicin	SQSPGNCASCSNSASANCTGGLG	mcbB	LAP
Y-proteusin	pcpA	VTAVGGVTGGSGIYGPIQAMYGAVVGDPKPGKDWGWRFPSPLPKPSPIPSP-WKPPV DVQPMYGVVVSNDS	pcpX	Proteusin
S+41	thioviridamide	SVMAAAASIALHC	L_biotic_typeA (PF04604)	Thioamitide
H+44	thioviridamide	SVMAAAASIALHC	tvaG	Thioamitide
H+14	microcyclamide	AFDGDEAS	Unknown	Cyanobactin
F+16	thiomuracin A	SCNCFCYICCSCSSA	MibO (PF00067)	Thiopeptide

Supplementary Table 6: The list of RiPP modifications considered in this study, along with a peptide example, the genes responsible for each modification, and their class. The chemical modifications are visualized in Supplementary Figure 22. The RiPP classes listed in the table are derived from the findings of [10] and [11]. (X = any amino acid)

Model Name	Embedding Size	Accuracy
(p)	100	**69.93**%
(q)	25	52.45%
(r)	50	61.75%
(s)	150	67.13%
(t)	200	out of memory

(A) Effect of Embedding Size on Accuracy

Model Name	Stride Size	Kernel Size	Accuracy
(p)	1	5	**69.93**%
(u)	1	3	63.99%
(v)	3	5	66.78%
(w)	1	6	69.58%

(B) Effect of CNN Kernel Settings on Accuracy

Model Name	ReLU before CRF	Dropout after Embedding	Dropout before CRF	Accuracy
(p)	no	no	no	69.93%
(x)	no	no	yes	61.19%
(y)	no	yes	no	68.53%
(z)	no	yes	yes	72.73%
(za)	yes	no	no	58.02%
(zb)	yes	no	yes	68.53%
(zc)	yes	yes	no	66.78%
(o)	yes	yes	yes	**73.08**%

(C) Effect of Regularization on Accuracy

Supplementary Table 7: Hyperparameter selection for orf2core. Various settings of embedding size, stride and kernel size, and skip connection settings are explored in parts (A), (B) and (C). All the models are designed based on the architecture in model (o). Models (p), (q), (r), (s) and (t) are identical to model (o), with the distinction that no ReLU or dropouts are applied, and embedding size changes between 25 to 200. Models (p), (u), (v), and (w) are identical to model (o), with the distinction that no ReLU or dropouts are applied, and various stride and kernel sizes are explored. The models were trained on a single RTX 2080 super GPU with 8G memory. The embedding size of 100, and stride and kernel size of 2 and 5 resulted in the highest accuracy. Models (p), (x), (y), (z), (za), (zb) and (zc) are identical to model (o), where embedding size is fixed to 100, stride and kernel sizes are fixed to 1 and 5, and various settings for ReLU and dropout regularizations are explored. Dropout of 0.5 after the embedding layer, and dropout of 0.2 plus a ReLU layer before the CRF layer resulted in highest accuracy of 73.08%.

MassiveID	NumSpectra	MassiveID	NumSpectra
MSV000078556	61,970	MSV000084117	12,287
MSV000078836	481,548	MSV000084475	638,641
MSV000078839	403,604	MSV000084674	28,854
MSV000078847	28,615	MSV000084723	136,043
MSV000078850	59,175	MSV000084771	442
MSV000078891	207,413	MSV000084884	85,130
MSV000078995	818	MSV000084945	1,550,594
MSV000079015	6,085	MSV000084954	15,513
MSV000079139	4,435	MSV000084989	808
MSV000079284	27,818	MSV000085003	8,526
MSV000079519	49,352	MSV000085018	44,223
MSV000080251	1,462,003	MSV000085023	2,372
MSV000080427	12,102	MSV000085026	1,872
MSV000081063	28,563	MSV000085027	426
MSV000081318	6,376	MSV000085032	2,660
MSV000081504	607	MSV000085123	769
MSV000082045	1,665,897	MSV000085158	25,606
MSV000082285	943	MSV000085159	188,737
MSV000082831	36,566	MSV000085179	7,853
MSV000083081	1,389	MSV000085180	9,095
MSV000083295	3,850	MSV000085192	1,634
MSV000083648	7	MSV000085214	3,284
MSV000083734	289,693	MSV000083738	409,245

Supplementary Table 8: 46 GNPS spectral datasets (8,013,443 spectra) analyzed in the paper. In each dataset we only analyze the mass spectral files that have corresponding genomes.

Supplementary References

1. Chigumba, D. N. *et al.* Discovery and biosynthesis of cyclic plant peptides via autocatalytic cyclases. *Nat Chem Biol* **18,** 18–28 (2022).
2. Bankevich, A. *et al.* SPAdes: a new genome assembly algorithm and its applications to single-cell sequencing. *J Comput Biol* **19,** 455–477 (2012).
3. Gibson, D. G. *et al.* Enzymatic assembly of DNA molecules up to several hundred kilobases. *Nature Methods* **6,** 343–45 (2009).
4. Sainsbury, F., Thuenemann, E. & Lomonossoff, G. pEAQ: versatile expression vectors for easy and quick transient expression of heterologous proteins in plants. *Plant biotechnology journal* **7,** 682–693 (2009).
5. Kersten, R. D. & Weng, J.-K. Gene-guided discovery and engineering of branched cyclic peptides in plants. *PNAS* **115,** E10961–E10969 (2018).
6. Jiao, W., Wu, Z., Chen, X., Lu, R. & Shao, H. Rhopeptin A: First Cyclopeptide Isolated from Rhodobryum giganteum. *Helvetica Chimica Acta* **96,** 114–18 (2013).
7. Auvin, C. *et al.* Mucronine J, a 14-Membered Cyclopeptide Alkaloid from Zizyphus mucronata. *Journal of Natural Products* **59,** 676–78 (1996).
8. Sivonen, K., Leikoski, N., Fewer, D. P. & Jokela, J. Cyanobactins-ribosomal cyclic peptides produced by cyanobacteria. *Applied microbiology and biotechnology* **86,** 1213–1225 (May 2010).
9. He, K., Zhang, X., Ren, S. & Sun, J. *Deep residual learning for image recognition* in *Proceedings of the IEEE conference on computer vision and pattern recognition* (2016), 770–778.
10. Agrawal, P., Khater, S., Gupta, M., Sain, N. & Mohanty, D. RiPPMiner: a bioinformatics resource for deciphering chemical structures of RiPPs based on prediction of cleavage and cross-links. *Nucleic acids research* **45,** W80–W88 (2017).
11. Montalbán-López, M. *et al.* New developments in RiPP discovery, enzymology and engineering. *Natural Product Reports* **38,** 130–239 (2021).
